# Supplementary material for: TAME trial: a multi-arm phase II randomised trial of four novel interventions for malnutrition enteropathy in Zambia and Zimbabwe - a study protocol
Source: BMJ Open. 2019 Nov 14;9(11):e027548. doi: 10.1136/bmjopen-2018-027548 (PMC6887014; doi:10.1136/bmjopen-2018-027548)
Supplement: Supplementary data [file bmjopen-2018-027548supp001.pdf]

Therapeutic approaches to malnutrition enteropathy (TAME): phase II trials of four novel interventions in children in Zambia and Zimbabwe

Zambia

Principal Investigator for Zambia: Dr Beatrice Amadi
Chief Investigator: Professor Paul Kelly

Participant Information Sheet
We are researchers from the University of Zambia School of Medicine and Lusaka Children's Hospital. We would like to invite you and your child to be part of this research project. You should only agree that your child could take part if you want to; it is entirely up to you. If you choose your child not to take part, there will be no disadvantages. Choosing not to take part will not affect your child's treatment in any way, now or in the future. Please read or listen to the following information carefully before you decide that your child can take part. If you decide to take part, you will be asked to sign a consent form to record your agreement. You are free to withdraw at any time during your child's participation without giving a reason.

Why we are doing this trial
The purpose of this trial is to develop extra treatments to help in the full recovery of children with Severe Acute Malnutrition (SAM). We know that children with SAM are at increased risk of dying. We know that malnutrition causes damage to the gut (intestine), which can allow it to become 'leaky'. We believe that if this does not heal properly, children do not recover from malnutrition. We want to try giving new medicines to heal the damaged intestine.

Why has my child been chosen?
We have identified that your child has severe acute malnutrition with complications. We want to include your child in a trial of some new treatments.

What the study involves
There are four new treatments which we want to compare against the treatments we currently use. Current treatments closely follow the guidelines of the World Health Organisation, including special feeds and fluids, and antibiotics. We do not know if these new treatments are better than the treatments we already have so we need to compare them in a randomised clinical trial. This means that the choice of treatments is made randomly, by chance (like the flip of a coin). For one out of five children, the treatment will remain exactly the same care as we give now. For four out of five children, we will give all the current treatments plus a new drug. A set of envelopes has been prepared with numbers on. If you agree that your child is to be included in the trial, a study number will be given. Once that number has been given we will open the envelope with that number and see which new treatment should be given, or if your child should not be given a new treatment. It is very important that you understand these two principles of a randomised trial:

1. We genuinely do not know which treatment is best, or if they are better or worse than what we do already.

Kelly P, *et al. BMJ Open* 2019; 9:e027548. doi: 10.1136/bmjopen-2018-027548

2. Children will be allocated a treatment by chance.

Once the envelope has been opened, we will follow the treatment course indicated on the contents of the envelope. We will not choose the treatment for your child.

The treatments

There are four possible treatments, but your child will only receive one of these at most. We do not know which one until the envelope is opened. All of these medicines need to be given for 2 weeks in hospital, not at home:

1. **Colostrum**. When mothers give birth, the first type of milk that comes from the breast is colostrum, which is a yellowy colour. This is full of protective substances for the baby and we think it also may help to heal the gut. We have obtained colostrum from cows as one of the new treatments. It is as safe as the cow’s milk we all drink in our tea, but there is the small possibility that it may be too rich for babies with severe acute malnutrition and may cause or worsen diarrhoea. We will watch out for such effects. It is given by mouth or down the gastric tube.
2. **N-acetyl glucosamine (GlucNac)**. This is a sort of starch which we hope will build up the protective mucus which lines our intestines. There is the small possibility that it may be too rich for children with severe acute malnutrition and may cause or worsen diarrhoea. We will watch out for such effects. It is given by mouth or down the gastric tube.
3. **Teduglutide (TDG)**. This is a modified version of a natural hormone the body produces, which helps the gut to repair itself after injury. It has been used in children with very severe gut failure, and has helped them to absorb more nutrients from the milk they get. A recent trial in the USA and the UK shows that it is safe, but it can cause vomiting and fever in about one in ten children who receive it for 3 months. We think it is unlikely that this will occur when the treatment is given for 14 days, but we will watch out for that. It is given as an injection under the skin, once every day.
4. **Budesonide**. This is a steroid, used to help heal the gut in children with other sorts of gut disease. Steroids can have side effects, including fluid accumulation and high levels of sugar in the blood and weakened resistance to infection, but usually only when they are given for longer periods. Budesonide is designed to treat gut inflammation without being absorbed, but we will watch out for any effects. It is given by mouth or down the gastric tube.

The investigations your child will have

When your child enters the study, he/she will have a full medical assessment and examination. All these tests are completely safe. We will also ask for an additional blood sample, a stool sample, and a saliva sample, then we do a simple test which involves drinking a small amount of a sugary liquid and collecting two urine samples. To reduce the pain of taking blood, we can use a jelly on your child’s skin which very much reduces the discomfort. We will then take another set of blood samples after one week; and blood, urine, saliva and stool samples after two weeks. The total volume of blood we will collect for research is limited to a safe quantity (2ml/kg body weight in total during the trial). We will carry out HIV testing as part of the tests on blood samples; the results will be completely confidential even if it is negative. All children will be monitored daily by our team of doctors and nurses for the full two weeks, and then we will ask you to come back

on the 28th day so that we can give your child a final growth check and ask a few questions.
In addition, between days 14 and 18 we will carry out an endoscopy. This means examining the inside of the stomach and intestine with a small flexible tube, through which we can collect tiny pieces of the lining of the intestine, each about the size of the head of a match. Although this sounds rather worrying, in fact this is quite safe because we have good facilities and a great deal of experience. The endoscopy requires giving anaesthetic injections; this is done by an anaesthetist who monitors the children very carefully.

If you would like, we would be very happy to show you the endoscopy unit and equipment at UTH so you have a better idea of what is involved. We can also show you the laboratories so you can see what we do with the samples we take, and how we analyse them.

How many children will be in the study?
The study is being conducted in Zambia and Zimbabwe. There will be 225 children in total in the study.

What we do with your child's samples
We only use the samples you provide for research. Any samples that we do not need for research will be destroyed. We have no need to use the samples you provide for any other purpose, including giving them to people who are not involved in the research. It is also against the law to do so.

We do nearly all of the research analysis in the laboratories at UTH. Some of the tests will need to be done by our collaborators in the UK or USA, so some of your child's samples may be sent there. These collaborating laboratories are not allowed to do anything with the samples that isn't to do with research, including selling them or giving them to people who aren't involved in the research. We are very strict on this and Prof Kelly can give you absolute reassurance that the samples are only used for this research. Once the research is finished they will be destroyed.

We do many different measurements on the samples you provide, and if you are interested we can demonstrate and explain some of these to you, including how the samples are stored.

What we do with the information you provide
During the research we collect information about you and your child. We store the information on computers and remove your name and your child's name and any other ways of being identified, so that there is no way of telling who the information refers to. Any and all information you give us is confidential. This means that we are not allowed to tell anyone about you or your child, even if they are friends or family.

How this study might benefit you

Kelly P, et al. BMJ Open 2019; 9:e027548. doi: 10.1136/bmjopen-2018-027548

We hope that the new treatment will speed up your child's recovery from SAM. However, because these treatments are new, we do not know at this stage whether they will work and we cannot therefore guarantee any benefits for your child. Your child will continue to receive the best possible care for malnutrition as well as any new treatments we give.

We hope that our learning from this trial will benefit children with SAM in the future.

<u>Are there any risks to participating in this study?</u>
This is a research project, and these treatments have never before been used in children in Zambia with severe acute malnutrition. We have already explained the possible risks of these treatments, and that we have designed the doses used to minimise these risks. We have considered this carefully and done our best, but the possibility remains that some children might experience adverse reactions to these treatments. We can promise you that we will watch out carefully for such reactions and that we will provide the best possible care in the event that a reaction does occur.

<u>What if I don't want my child to take part in the study or I want to withdraw later?</u>
Participation in this study is voluntary. It is completely up to you whether or not your child participates, or if you join the trial but decide to withdraw later. If you decide for your child not to participate, it will not affect the treatment your child receives now or in the future. Whatever your decision, it will not affect your relationship with the staff caring for you.

<u>Insurance and what to do if you have questions or concerns</u>
In the unlikely event that your child is harmed as a result of this trial, Queen Mary University of London has agreed that you will be compensated, provided that, on the balance of probabilities, an injury was caused as a direct result of the intervention or procedures your child received during the course of the study. These special compensation arrangements apply where an injury is caused to your child that would not have occurred if s/he were not in the study. These arrangements do not affect your right to pursue a claim through legal action.

It is up to you to decide whether or not to take part. If you do decide to take part you will be given this information sheet to keep and be asked to sign a consent record form.

If you have any questions or concerns about the study or how it is conducted, you can contact Professor Kelly's and Dr Amadi's team in the hospital at any point

Contact details are:

Professor Paul Kelly
Endoscopy Unit, Clinic 5,
University Teaching Hospital
Lusaka
Tel 0211 252269/0955 295493
or Dr Beatrice Amadi
Ward A07
Lusaka Children's Hospital

Kelly P, *et al. BMJ Open* 2019; 9:e027548. doi: 10.1136/bmjopen-2018-027548

Lusaka
tel: 0966 752739
If you are not happy to do this, or are not happy with the advice you get from Professor Kelly's and Dr Amadi's team, you can contact:

The University of Zambia Biomedical Research Ethics Committee,
University of Zambia School of Medicine,
Ridgeway Campus,
PO Box 50110,
Lusaka.
tel: 0211 250753 and speak to Lorna

A COPY OF THE INFORMATION SHEET AND CONSENT RECORD WILL BE GIVEN TO YOU FOR SAFE KEEPING, AND A COPY WILL BE RETAINED BY THE STUDY TEAM

Kelly P, et al. BMJ Open 2019; 9:e027548. doi: 10.1136/bmjopen-2018-027548

Please complete this form after you have read or listened to the Information Sheet and/or listened to an explanation about the research.

Therapeutic approaches to malnutrition enteropathy (TAME)
UNZABREC reference: tbc

1. I have read or listened to the Participant Information Sheet for this study (version 0.7, dated 28th March, 2018).
2. The research trial has been explained to me. I understand what the research involves and what is required of me.
3. I understand that my child will have a blood test for HIV.
4. I understand that samples obtained from my child may be sent abroad for research purposes only, and that after use the samples will either be returned to Zambia for storage or destroyed.
5. I understand that there is no obligation for me to allow my child to participate in the research study, and that I do not have to give a reason for not participating.
6. I understand that if I decide at any time during the research that I no longer wish my child to participate, I can notify the researchers involved and be withdrawn from it immediately, without giving a reason.
7. I consent to the processing of our personal information for the purposes of this research study. I understand that such information will be treated as strictly confidential at all times and that any identifiable information about me or my child will be available only to members of the study team.

Participant's statement:
I ____ have read or listened to statements 1-7 above and I agree to allow my child ______________________________ to take part in the study.

Signed: Date:

Witnessed: Date:

Investigator's Statement:
I ____ confirm that I have carefully explained the nature, demands and any foreseeable risks of the proposed research to the family.

Signed: Date:

Kelly P, *et al*. *BMJ Open* 2019; 9:e027548. doi: 10.1136/bmjopen-2018-027548
