# Supplementary material for: TAME trial: a multi-arm phase II randomised trial of four novel interventions for malnutrition enteropathy in Zambia and Zimbabwe - a study protocol
Source: BMJ Open. 2019 Nov 14;9(11):e027548. doi: 10.1136/bmjopen-2018-027548 (PMC6887014; doi:10.1136/bmjopen-2018-027548)
Supplement: Author's manuscript [file bmjopen-2018-027548.draft_revisions.pdf]

BMJ Open

BMJ Open is committed to open peer review. As part of this commitment we make the peer review history of every article we publish publicly available.

When an article is published we post the peer reviewers' comments and the authors' responses online. We also post the versions of the paper that were used during peer review. These are the versions that the peer review comments apply to.

The versions of the paper that follow are the versions that were submitted during the peer review process. They are not the versions of record or the final published versions. They should not be cited or distributed as the published version of this manuscript.

BMJ Open is an open access journal and the full, final, typeset and author-corrected version of record of the manuscript is available on our site with no access controls, subscription charges or pay-per-view fees (http://bmjopen.bmj.com).

If you have any questions on BMJ Open's open peer review process please email info.bmjopen@bmj.com

BMJ Open

The TAME trial: a multi-arm phase 2 randomised trial of four novel interventions for malnutrition enteropathy in Zambia and Zimbabwe

Journal:	*BMJ Open*
Manuscript ID	bmjopen-2018-027548
Article Type:	Protocol
Date Submitted by the Author:	27-Oct-2018

Complete List of Authors:	Kelly, Paul; Barts and The London School of Medicine<br>Bell, Lauren; London School of Hygiene and Tropical Medicine<br>Amadi, Beatrice; 2Tropical Gastroenterology and Nutrition Group, University of Zambia, Lusaka, Zambia<br>Bwakura, Mutsa<br>VanBuskirk, Kelley<br>Chandwe, Kanta; Tropical Gastroenterology and Nutrition Group, University of Zambia, Lusaka, Zambia<br>Chipunza, Miyoba; Tropical Gastroenterology & Nutrition Group<br>Ngosa, Deophine; Tropical Gastroenterology and Nutrition Group, University of Zambia, Lusaka, Zambia<br>Chulu, Nivea; 2Tropical Gastroenterology and Nutrition Group, University of Zambia, Lusaka, Zambia<br>Hill, Susan; Great Ormond Street Hospital For Children NHS Trust,<br>Murch, Simon ; Warwick Medical School, University of Warwick, Division of Metabolic and Vascular Health<br>Playford, Raymond; University of Plymouth School of Biological Sciences<br>Prendergast, Andrew ; Queen Mary University of London,

Keywords:	Paediatric gastroenterology < GASTROENTEROLOGY, Tropical medicine < INFECTIOUS DISEASES, NUTRITION & DIETETICS, Paediatric gastroenterology < PAEDIATRICS, Clinical trials < THERAPEUTICS

SCHOLARONE™
Manuscripts

The TAME trial: a multi-arm phase 2 randomised trial of four novel interventions for malnutrition enteropathy in Zambia and Zimbabwe

Paul Kelly[1,2,3], Lauren Bell[3], Beatrice Amadi[2], Mutsa Bwakura-Dangarembizi[4,5], Kelley VanBuskirk [2], Kanta Chandwe[2], Miyoba Chipunza[2], Deophine Ngosa[2], Nivea Chulu[2], Susan Hill[6], Simon Murch[7], Raymond Playford[1], Andrew J Prendergast[1,5]

[1]Barts & The London School of Medicine, Queen Mary University of London, London, UK; [2]University of Zambia School of Medicine, Lusaka, Zambia; [3]London School of Hygiene and Tropical Medicine, London, UK; [4]Department of Paediatrics and Child Health, College of Health Sciences, University of Zimbabwe, Harare, Zimbabwe; [5]Zvitambo Institute for Maternal and Child Health Research, Harare, Zimbabwe; [6]Great Ormond Street Hospital, London, UK; [7]University Hospital Coventry & Warwickshire, Coventry, UK.

Corresponding author:

Professor Paul Kelly MD FRCP

Blizard Institute

Barts & The London School of Medicine

Turner Street

London E1 2AD

UK

tel: 020 7882 2643

For peer review only

email: m.p.kelly@qmul.ac.uk

For peer review only

For peer review only - http://bmjopen.bmj.com/site/about/guidelines.xhtml

ABSTRACT

Introduction

Severe acute malnutrition (SAM) in children in many countries still carries unacceptably high mortality, especially when complicated by secondary infection or metabolic derangements. New therapies are urgently needed and we have identified mucosal healing in the intestine as a potential target for novel treatment approaches.

Methods and analysis

The TAME trial (therapeutic approaches for malnutrition enteropathy) will evaluate four novel treatments in an efficient multi-arm design. In three hospitals in Zambia and Zimbabwe, 225 children with SAM will be randomised to one of these treatments or to standard care, once their inpatient treatment has reached the point of transition from stabilisation to increased nutritional intake. The four interventions are budesonide, bovine colostrum or N-acetyl glucosamine given orally or via nasogastric tube, or teduglutide given by subcutaneous injection. The primary endpoint will be a composite score of faecal inflammatory markers, and a range of secondary endpoints include clinical and laboratory endpoints. Treatments will be given daily for 14 days, and evaluation of the major endpoints will be at 14-18 days, with a final clinical evaluation at 28 days. In a subset of children in Zambia, endoscopic biopsies will be used to evaluate the effect of interventions in detail.

Ethics and dissemination

The study has been approved by the University of Zambia Biomedical Research Ethics Committee (006-09-17, dated 9th July, 2018), and other local and international institutional review boards in Zambia, Zimbabwe and the UK. Caregivers will provide written informed consent for each participant. Findings will be disseminated through peer-reviewed journals, conference presentations and to caregivers at face-to-face meetings.

ARTICLE SUMMARY; strengths and limitations of the trial

Strengths

- Efficient multi-arm trial design
- Evaluation of potential efficacy of four novel interventions for a life-threatening disorder of great importance for global health
- Measurement of a broad range of biomarkers and measures of mucosal pathology

Potential limitations

- Only 14 days of treatment will be tested; although longer treatment may be effective, the

For peer review only

For peer review only - http://bmjopen.bmj.com/site/about/guidelines.xhtml

greatest need is for treatments which confer benefit in the period of highest mortality

- There is no consensus on optimal markers of mucosal healing, hence the need for a wide range of endpoints.

For peer review only

BACKGROUND AND RATIONALE

Nutritional disorders are glaring examples of health inequalities between high- and low-income countries, and within low-income countries. Malnutrition underlies almost half of all child deaths globally and therefore contributes enormously to the unacceptably high under-5 mortality rates in these regions[1]. Chronic undernutrition, which usually manifests as stunting (poor linear growth), affects 40% and 27% of children in Zambia and Zimbabwe, respectively[2], and is associated with increased mortality[3], reduced neurodevelopmental potential and decreased long-term economic productivity[4]. Acute malnutrition usually manifests as wasting (loss of tissue), with or without oedema, and is the most conspicuous of all nutritional disorders. Severe acute malnutrition (SAM) carries the highest mortality[5], particularly if associated with medical complications. HIV has changed the epidemiology, pathogenesis and clinical presentation of SAM, and children with both conditions have a particularly high mortality[6].

Over the last two decades, three key developments in the approach to treatment have improved the outcomes of SAM: standardised management protocols, ready to use therapeutic food (RUTF), and community management of acute malnutrition (CMAM), which enables identification and management of children without medical complications of SAM[7,8]. However, severely malnourished children with medical complications still require hospitalisation,[9] and often fail to respond to treatment[10]. Inpatient mortality among children with complicated SAM remains up to 35%[5,11]. Even after discharge, children have a poor prognosis, with up to 42% mortality documented over the subsequent year[6]. In our experience, it is a subgroup of children with SAM and acute or persistent diarrhoea who pose the most difficult management challenges, although the vast majority of children with SAM and/or stunting have a degree of enteropathy[11,12,13].

Recent studies[14,15] have taught us a great deal about the contribution of enteric infections to dysfunction of the small intestine in malnourished children. A high pathogen burden causes damage to the mucosa which exacerbates nutritional impairment and leads to further susceptibility to infection and impaired epithelial regeneration, in a cyclical process first described in the 1970s[16,17]. This mucosal damage in SAM we refer to as *malnutrition enteropathy*, which is characterised by multiple epithelial breaches, microbial translocation from the gut lumen to the systemic circulation, and systemic inflammation[18]. Epithelial breaches are present in histological sections, seen in haematoxylin/eosin-

For peer review only - http://bmjopen.bmj.com/site/about/guidelines.xhtml

stained sections and using immunofluorescence for claudin 4 and E-cadherin[18,19]. In parallel studies in adults, we demonstrated that these lesions occur *in vivo* using confocal laser endomicroscopy[19]. We have also identified a consistent pattern of blunted epithelial repair, with reduced glucagon-like peptide 2 (GLP2) in serum, reduced trefoil factor 3 in duodenal aspirates[19], and a strong transcriptomic signature of impaired mucosal defence. We also observed two further immunological abnormalities: low-level false positive coeliac-like autoantibodies[18], and upregulation of SMAD7 somewhat similar to the pattern seen in Crohn's disease[20]. We have previously reported that markers of microbial translocation and systemic inflammation were dramatically increased in children with SAM in Zambia compared to healthy controls[18]. Together, these abnormalities indicate there is substantial structural and functional damage to the small intestine[21], and that this primary gut pathology is associated with systemic sequelae. Importantly, biomarkers of these processes have been associated with mortality among children hospitalized with complicated SAM[21].

Current treatment guidelines for SAM are not well supported by an evidence base, and there is a dearth of clinical trial data; in particular, there are no specific interventions to target enteropathy in SAM[5]. In a systematic review[22], only three trials were found which inform management of SAM and persistent diarrhoea, and no trials dealing with the HIV-infected child. We therefore believe that novel therapeutic approaches are urgently needed, and that a series of small phase 2 trials will help guide development of a new generation of treatments. These trials should focus on repairing damage to the small intestinal mucosa, as we now have substantial evidence that this plays a central role in the genesis of systemic inflammation, bacterial translocation and sepsis with all its adverse nutritional consequences. We propose evaluation of four new interventions: bovine colostrum, N-acetylglucosamine, teduglutide and budesonide.

OBJECTIVES

To determine if four new interventions (bovine colostrum, N-acetylglucosamine, teduglutide and budesonide) can ameliorate malnutrition enteropathy in children with severe acute malnutrition in Zambia or Zimbabwe. It is hoped that one or more of these interventions may be sufficiently promising to take through into phase 3 trials.

METHODS AND ANALYSIS

Trial design

For peer review only - http://bmjopen.bmj.com/site/about/guidelines.xhtml

There is emerging awareness that multi-arm Phase 2 clinical trials offer a more efficient approach to identification of new therapies than parallel conventional trial designs, in which one agent is evaluated against a control. Our recent work on the pathophysiology of malnutrition enteropathy suggests that mucosal healing may be central to reducing mortality, but we do not yet know the efficacy of several plausible therapeutic approaches. In order to evaluate these four new potential therapies efficiently and quickly, they will be compared side-by-side using biomarkers of pathophysiology as endpoints. This design has the following advantages:

i. The four novel therapies will each be evaluated against standard care;

ii. Allocation to all five treatment groups will run in parallel, so that all children will be randomised to any of the five groups at any point in time to avoid biases due to seasonal variation;

iii. The trial endpoints will evaluate a range of biomarkers that capture different domains of malnutrition enteropathy, to allow a comprehensive non-invasive assessment of mucosal healing;

iv. A subgroup of children (those recruited in Lusaka) will have small intestinal endoscopy in a unit with a track record of safety in children.

v. Endoscopic biopsy will provide evaluation of healing and ensure that the selected therapy does not induce unwanted immune or other effects;

vi. The overall trial design provides an efficient way of identifying one or more candidates for a future Phase 3 trial.

For peer review only

Study setting and participant recruitment

The study will be conducted in hospitals in Lusaka, Zambia, and Harare, Zimbabwe, which have experience of several previous studies of severe acute malnutrition[11,18,23]. Children hospitalised with SAM will be eligible for inclusion if they are aged 6-59 months, of either sex, are an inpatient in one of the children's wards of the participating hospitals, are within 72 hours of initiation of transition (from F75 feed to F100 or RUTF), and clinically stable. SAM is defined using WHO definitions: weight-for-length/height Z-score of less than -3, or mid upper arm circumference of less than 11.5cm, and/or bilateral pedal oedema. During the Stabilization Phase of treatment of SAM, readiness for transition is determined by the ability to finish F75 feed by mouth and reduction in oedema (if patient was

For peer review only - http://bmjopen.bmj.com/site/about/guidelines.xhtml

admitted with oedematous SAM). Such a child is deemed to be “clinically stable” and in general is alert, not hypothermic, hypoglycaemic and is not in shock or dehydrated.

Children will be excluded if they have weight less than 5kg, a neurological disability or oro-facial abnormality which would explain or partly explain poor feeding, haemoglobin concentration 6 g/dl or less, contraindication to any of the trial treatments (e.g. allergy to cow’s milk protein), or any underlying condition, other than HIV, which in the opinion of the investigator would put the subject at undue risk of failing study completion or would interfere with analysis of study results. If the caregiver is unwilling to remain in hospital for the duration of the study treatment (14 days) they will not be recruited.

Interventions: investigational medicinal products

Colostrum is the first liquid secreted by the lactating breast, and breast-fed children take it for approximately the first three days of life. It is similar to breast milk, but with higher protein content. Bovine colostrum is available as a high-protein bovine colostrum powder (Neovite) for use as a health-promoting nutritional supplement. Colostrum contains nutrients, immunoglobulins and growth factors, including epidermal growth factor (EGF) and insulin-like growth factor 1 (IGF-1); it has been shown to reduce intestinal permeability in adults[24]. Bovine colostrum is potentially allergenic in theory, though there is no evidence that cow’s milk products are harmful in children with SAM and the standard therapeutic feeds (F75 and F100 recommended by WHO) contain milk proteins.

N-acetyl glucosamine (GlcNAc) is a natural amino-sugar present on every cell surface. All breast-fed children consume GlcNAc in human milk throughout lactation[25]. Impaired glycosylation of glycosaminoglycans has been noted for many years in oedematous malnutrition, with reduced concentrations of glycosaminoglycans in blood, urine, kidney, brain and small intestine. Specific consequences of reduced heparan sulphate expression include gut epithelial leakiness with hypoalbuminaemia[26]. GlcNAc administration has been demonstrated to restore the intestinal epithelial charged barrier in Crohn's disease[27]. As with all sugars, GlcNAc may theoretically induce osmotic diarrhoea if not absorbed in the small intestine. Although this has not been observed clinically in animals or in older children, we will use a dose escalation schedule to minimise the chance of this happening.

For peer review only - http://bmjopen.bmj.com/site/about/guidelines.xhtml

Teduglutide is a long-acting form of GLP2 which has proven efficacy in intestinal failure, improving absorption and reducing the need for parenteral support[28]. GLP2 is a hormone secreted by L cells in the terminal ileum, which drives epithelial repair and mediates intestinal adaptation by increased cellular proliferation and villus hypertrophy. Teduglutide will be given by subcutaneous injection (0.05mg/kg/day) daily for 14 days. In a recently published description of tolerability of 12 weeks of teduglutide in children in the UK and the USA[29], vomiting was attributed to teduglutide in 10% of recipients and pyrexia in 14%. No children had treatment withdrawal because of adverse events. Three adverse events were considered of special interest (intestinal obstruction, fluid overload and biliary derangements) but none were observed.

Budesonide is a corticosteroid which reduces inflammation in the gut but is then rapidly broken down in the liver which minimises systemic effects. Budesonide is standard therapy for Crohn's disease, and can be used for refractory coeliac disease. Since malnutrition enteropathy is characterized by intestinal inflammation, with infiltration of activated T cells[30], an anti-inflammatory approach is rational. A prior trial of mesalazine in Kenya confirmed an immuno-modulatory approach is safe in the setting of SAM[31], but targeting the small, rather than large, intestine with a more potent agent may be more effective. Budesonide may cause immunosuppression and other corticosteroid effects (oedema, hypertension, glucose intolerance, osteoporosis), but usually only after longer-term administration. It is the corticosteroid of choice for intestinal disorders because it causes fewer adverse events than prednisolone because of low systemic absorption and first-pass clearance by the liver. The dose will be tapered (1mg orally three times daily for 7 days, then 1mg twice daily for 4 days, then 0.5mg twice daily for 3 days) to mitigate any possible effects of adrenal suppression.

Trial procedures

The trial schema is shown in Figure 1. Children hospitalised with SAM will be enrolled from any of three hospitals (Lusaka Children's Hospital, Lusaka, Zambia; Parirenyatwa Hospital, Harare, Zimbabwe; Harare Central Hospital, Harare, Zimbabwe) once they have completed the stabilization phase of nutritional rehabilitation and are at the point of transition from F75 therapeutic milk (low-calorie feed) to ready-to-use therapeutic food (RUTF) or F100 milk (feeds richer in calories). Children will be randomised in an allocation ratio of (1:1:1:1:1) to either Colostrum, GlcNAc, Teduglutide, Budesonide or Standard Care and followed for 14 days in hospital (Table 1). All children will receive Standard Care

For peer review only - http://bmjopen.bmj.com/site/about/guidelines.xhtml
For peer review only

according to WHO guidelines[32], and treatment with trial medications will commence on day 2 of transition.

Randomisation codes will be prepared in advance by the trial statistician, and treatment allocation will be revealed after enrolment by opening sealed envelopes held by the trial coordinators in each of the three study sites. Randomisation codes will be generated in permuted blocks and stratified by study site. Varying block sizes will be used; block size and distribution will not be disclosed to the study clinical teams. There will be no attempt at blinding as the interventions are readily distinguishable (e.g. teduglutide is given as subcutaneous injection, so to mask all interventions would require administration of several placebo formulations). However, the primary endpoints are biomarkers so laboratory staff and data analysts will be blinded to randomised group.

Outcomes and endpoints

The primary endpoint for this trial will be measured on day 14 (allowable window 14 to 18 days) after initiating treatment by analysis of faecal biomarkers. Gut inflammation will be measured as a composite score of faecal myeloperoxidase, neopterin and alpha-1 antitrypsin, as described below. Secondary endpoints (Table 2) will be measured by daily clinical review (for clinical endpoints) during the intervention period and by collection of biological specimens on day 14 (allowable window 14 to 18 days) after initiating treatment. As endoscopic biopsies will be collected from trial participants in Lusaka, we anticipate that at least twenty biopsy scores will be available for each trial arm. Few trials have used histological endpoints in SAM, so this component of the trial is comparatively novel and therefore exploratory.

Sample size and data analysis

Kosek et al[33] examined established markers of intestinal inflammation which can be used to obtain population-based measures of intestinal injury and altered intestinal function. They developed an environmental enteropathy (EE) activity score which is a composite score of three stool markers for mucosal inflammation. The composite score is derived from measurements of faecal myeloperoxidase (MPO), neopterin (NEO) and alpha1-antitrypsin (AAT). These are stable compounds, relatively resistant to proteolysis and therefore suitable as biomarkers.

For peer review only - http://bmjopen.bmj.com/site/about/guidelines.xhtml

The weighted EE score is given in Equation 1:

Equation 1

EE score = 2 x (AAT category) + 2 x (MPO category) + 1 (1 x NEO category)

We will use the principle of a weighted enteropathy score as our primary outcome. We will not trichotomise the variables as Kosek et al[33] did, but keep the score as a continuous composite variable. This is because we are interested in gauging the potential magnitude of the effect these treatments have, and applying a cut-off value to a continuous composite score could mask or discard information[34,35]. The modified Enteropathy score is given in Equation 2, with modification of weighting of units to bring measurements into a comparable arithmetical range:

Equation 2

EE biomarker score = 2x(AAT in mg/g) + 0.2x(MPO in µg/ml) + 1x(neopterin in µmol/l).

We will have a control arm, so can understand the effect of the treatment against a reference standard.

There are no available data from children with SAM to draw upon for an understanding of the baseline variance of the EE biomarker score. The primary outcome response from each patient is the EE biomarker score at 14 days (window 14-18 days) after treatment initiation, adjusted for baseline values. We have assumed that the EE biomarker score is normally distributed with a common standard deviation. To determine the sample size, we drew upon two methods to establish an effect size. We assumed the difference would be larger than the likely inherent imprecision in the measurements of each outcome, and we have defined the magnitude of the effect on a standardised scale. To detect a medium/large effect of Cohen's *d* effect size of 0.6, with 90% power and 90% confidence, and a conservative correlation between baseline and follow-up estimate of 0.5, we will need a sample size of 36 per group across 5 groups to analyse with the ANCOVA method (180 in total to be treated). We expect there to be approximately 10% loss to follow up due to deaths, and 10% loss to follow up due to other reasons, such as drug intolerance, withdrawal and missing specimens. Adjusting our sample size of 180 for 20% loss to follow-up, we therefore aim to randomise 225 patients in total (45 in each group).

For peer review only - http://bmjopen.bmj.com/site/about/guidelines.xhtml

We will use a mixed effect ANCOVA model to compare the environmental enteropathy activity score and secondary endpoints in each group against the control group, adjusting for several core covariates: sex (male/female); oedema (yes/no); HIV status (yes/no); diarrhoea (yes/no); baseline WLZ scores (continuous); baseline biomarker/histology scores (continuous) and trial site. Treatment effects will be deemed statistically significant if the p-value is less than or equal to 0.10 when compared to the control arm, for all 4 treatments. This less stringent cut-off has been chosen to reduce the likelihood of rejecting a potentially valuable treatment which might show benefit in a phase 3 trial. The DMC and TSC will contribute to a discussion at the end of the trial as to which, if any, of these treatment effects are most clinically significant.

We will not undertake any adjustments of the false positive (type I) error rate, as the aim of this trial is to inform the treatment development process for this population, which would lead to a Phase III trial if a degree of efficacy is observed. The general consensus is that adjustment for the type I error rate is not required in exploratory multi-arm multi-stage trials in Phase II within the treatment development framework[36].

If patients are discharged from hospital sooner than day 14, we will measure the primary endpoints as close as the discharge date as possible, and carry this observation forward as the primary outcome.

Safety reporting

All adverse events will be reviewed for causality, expectedness and severity. Serious adverse events will be reported urgently to the Trial Management Group for evaluation, and all serious adverse reactions will be regarded as unexpected (i.e. SUSARs). There are two adverse events of special interest for teduglutide: fluid overload and intestinal obstruction, as these have been reported in adults on long-term teduglutide. Complicated SAM has an inpatient mortality rate of around 15%, so we anticipate up to 45 deaths in the trial; however, all deaths, regardless of causality, will be reported to the local ethics committees in Zimbabwe and Zambia according to local reporting policies. The Trial Management Committee (TMC), together with the DMC and trial steering committee (TSC), will review the pattern of any SAEs and SUSARs in real time, and is empowered to introduce urgent special measures if the profile of adverse events suggests this is required.

For peer review only - http://bmjopen.bmj.com/site/about/guidelines.xhtml

ETHICS AND DISSEMINATION

Consent

Ethical approval for the trial will be sought in each country. Trial nursing and medical staff will identify eligible children whose primary caregivers will then be approached to begin the process of discussing the trial and the written, translated, information sheet in the most appropriate language. The consent process will include honest discussions of risks and benefits of the interventions, the concept of randomisation, and the purpose and intended use of samples collected, which is an important issue in this trial as the primary endpoints are all laboratory biomarkers. The consent process will continue throughout the trial, but all parents/guardians will sign to record that they have given informed consent to their child's participation, before any study procedures commence. Parents of children who cannot read will have the information sheet read out to them in the presence of family members before consent is accepted and recorded using a thumbprint. We have pioneered the use of participant visits to laboratories to facilitate valid consent[37]. This opportunity will be afforded to these parents and guardians. At least 24 hours will be available for consideration of consent; in practice, 2-3 days is the norm in Zambia and Zimbabwe because mothers/carers usually need to discuss enrolment with other family members.

Dissemination of results

Dissemination of results to participants' families will take place through caregiver results meetings. We will disseminate results within the academic institutions in which the trial will be conducted and to national health research authorities. Trial results will be published following CONSORT guidelines. Improvements in care will ultimately occur through change in evidence-based practice. By collaborating with colleagues on design of a future interventional trial based on our results, and by sharing specimens with other research groups, we intend to push forward the research agenda for children with SAM[38]. We will also use this trial as an opportunity to increase awareness in the UK of the complexity of malnutrition. The Centre of the Cell (www.centreofthecell.org) at Queen Mary University of London is a unique, state-of-the-art science education centre with extensive experience of translating complex medical research into innovative science communication tools. We are currently working with CoC to develop a game to explain the causes of undernutrition, including the role of the gut in nutrition, with endoscopy footage, animations and interactive gameplay . The game

For peer review only - http://bmjopen.bmj.com/site/about/guidelines.xhtml

will be reformatted for tablets for use in Harare and Lusaka. The production of multiple formats of the tool will facilitate adults and children in the UK and Africa to access this resource and learn about current research on SAM.

Time frame and study status

The trial has been granted ethical approval by the University of Zambia Biomedical Research Ethics Committee (006-09-17, dated 9th July, 2018) and applications have been submitted for ethics and pharmaceutical regulatory approval in Zambia and Zimbabwe. Once recruitment begins, the trial is anticipated to take two years.

DISCUSSION

In the belief that reducing mortality from SAM will require novel therapeutic approaches, and recognising abundant evidence of impaired intestinal function, we have identified mucosal healing in the small intestine as a target for intervention. The TAME trial will employ a multi-arm design to identify one or more of four interventions for taking forward into phase 3 trials. The strengths of this trial include an efficient design, which will evaluate four novel interventions against standard care, and very extensive assessment of mucosal healing and biomarkers to provide the greatest chance of observing potential benefit. Limitations of the trial include the short duration of treatment, ongoing uncertainty about the optimal selection of biomarkers for accurate representation of intestinal absorptive and barrier function, and the complexity of data interpretation in this group of children with serious morbidity and high predicted mortality.

We have considered carefully the issue of duration of treatment. Inpatient treatment of SAM lasts usually between 1-2 weeks and it is in this narrow time window that most, though not all[6], of the mortality occurs. If novel therapies for reduction of mortality in SAM are to be useful at scale, they must confer benefit quickly, during the 1-2 week period immediately after admission. While it would be of great interest to evaluate more extended periods of therapy, the most urgent need is for new treatments in this short period of maximum morbidity and mortality. In future trials of any promising interventions, the timing of introduction of novel therapies would be an explicit research question, as they may also be of value earlier in treatment, or continued for a longer duration, including after discharge. However, for this phase II trial, we will only enrol children once they are clinically stable, at

For peer review only - http://bmjopen.bmj.com/site/about/guidelines.xhtml

the transition phase, and provide treatment over 14 days during the typical period of hospitalisation.

Two of these agents (bovine colostrum and N-acetyl glucosamine) are nutraceuticals, food-derived and generally regarded as safe. While this is very reassuring about the low probability of adverse events, it is hard to quantify that probability with any accuracy. Regulatory agencies are more familiar with IMPs which have well-described adverse event profiles based on careful phase 1 trials, so paradoxically the level of scrutiny of any potential adverse events in the TAME trial will be higher than if full SmPCs were publically available, despite the expected low risk of significant AEs.

In summary, we propose a phase 2 trial designed to target malnutrition enteropathy, which we believe is central to the pathogenesis of SAM, prevents recovery, and is not currently addressed by current interventions. Identification of promising novel treatment approaches would provide a strong rationale for a larger efficacy trial to reduce the unacceptably high morbidity, mortality and relapse among children with complicated SAM.

For peer review only

For peer review only - http://bmjopen.bmj.com/site/about/guidelines.xhtml

Authors' contributions

PK, AJP, BA and MB-D originated the trial idea and obtained funding. KvB and LB provided statistical expertise. KC, RP, SM and SH provided specific input into design of specific elements of the trial, particularly the principal interventions.

Funding statement

The TAME trial is funded by the Medical Research Council (MR/P024033/1). AJP and MB-D are funded by the Wellcome Trust (grants 093768/Z/15/Z and 107634/Z/15/Z, respectively).

Competing interests

The authors declare that they have no competing interests

Word count

3,891

For peer review only

For peer review only - http://bmjopen.bmj.com/site/about/guidelines.xhtml

References

1 Black RE, Victora CG, Walker SP, Bhutta ZA, Christian P, de Onis M, Ezzati M, Grantham-McGregor S, Katz J, Martorell R, Uauy R. Maternal and child undernutrition and overweight in low-income and middle-income countries. Lancet. 2013; 382: 427-51.

2 Zambia Demographic and Health Survey 2013-14; Zimbabwe Demographic and Health Survey 2015. Rockville, Maryland, USA: Central Statistical Office, Ministry of Health, and ICF International.

3 Olofin I, McDonald CM, Ezzati M, Flaxman S, Black RE, Fawzi WW, Caulfield LE, Danaei G. Associations of suboptimal growth with all-cause and cause-specific mortality in children under five years: a pooled analysis of ten prospective studies. PLoS One 2013 8: e64636.

4 Victora CG, Adair L, Fall C, Hallal PC, Martorell R, Richter L, Sachdev HS. Maternal and child undernutrition: consequences for adult health and human capital. Lancet 2008; 371: 340–357.

5 Lenters LM, Wazny K, Webb P, Ahmed T, Bhutta ZA. Treatment of severe and moderate acute malnutrition in low- and middle-income settings: a systematic review, meta-analysis and Delphi process. BMC Public Health. 2013; 13 Suppl 3: S23.

6 Kerac M, Bunn J, Chagaluka G, Bahwere P, Tomkins A, Collins S, Seal A. Follow-up of post-discharge growth and mortality after treatment for severe acute malnutrition (FuSAM study): a prospective cohort study. PLoS One 2014; 9: e96030.

7 Collins S, Dent N, Binns P, Bahwere P, Sadler K, Hallam A. Management of severe acute malnutrition in children. Lancet 2006; 368: 1992-2000.

8 Amadi B, Imasiku M, Sakala M, Banda R, Kelly P. Integration of HIV care into community management of acute childhood malnutrition permits good outcomes: retrospective analysis of three years of a programme in Lusaka. PLoS One 2016; 11: e0149218.

9 WHO Guideline: Updates on the management of severe acute malnutrition in infants and children. Geneva: World Health Organization; 2013.

10 Isanaka S, Langendorf C, Berthé F, Gnegne S, Li N, Ousmane N, Harouna S, Hassane H, Schaefer M, Adehossi E, Grais RF. Routine Amoxicillin for Uncomplicated Severe Acute Malnutrition in Children. N Engl J Med 2016; 374: 444-53.

11 Amadi BC, Kelly P, Mwiya M, Mulwazi E, Sianongo S, Changwe F, Thomson M, Hachungula J, Watuka A, Walker-Smith JA, Chintu C. Intestinal and systemic infection, HIV and mortality in Zambian children with persistent diarrhoea and malnutrition. J Ped Gastroenterol Nutr 2001; 32: 550-554.

12 Prendergast A, Kelly P. Interactions between intestinal pathogens, enteropathy and malnutrition in developing countries. Curr Op Infect Dis 2015; 29; 229-236.

For peer review only - http://bmjopen.bmj.com/site/about/guidelines.xhtml

13 Trehan I, Kelly P, Shaikh N, Manary MJ. New insights into Environmental Enteric Dysfunction. Arch Dis Child 2016; 101:741-4.

14 Kotloff KL, et al. Burden and aetiology of diarrhoeal disease in infants and young children in developing countries (the Global Enteric Multicenter Study, GEMS): a prospective, case-control study. Lancet 2013; 382: 209–22.

15 Platts-Mills JA, et al. Pathogen-specific burdens of community diarrhoea in developing countries: a multisite birth cohort study (MAL-ED). Lancet Glob Health 2015; 3: e564–75.

16 Scrimshaw, NS, et al. Monogr Ser World Health Organ, 1968. 57: 3-329.

17 Mata LJ. The Children of Santa Maria Cauqué: A Prospective Field Study of Health and Growth (International nutrition policy series) MIT Press: 1978.

18 Amadi B, Besa E, Zyambo K, Kaonga P, Louis-Auguste J, Chandwe K, Tarr PI, Denno DM, Nataro JP, Faubion W, Sailer A, Yeruva S, Brantner T, Murray JA, Prendergast AJ, Turner JR, Kelly P. Impaired barrier function and autoantibody generation in malnutrition enteropathy in Zambia. EBiomedicine 2017; 22: 191-199.

19 Kelly P, Besa E, Zyambo K, Louis-Auguste J, Lees J, Banda T, Soko R, Banda R, Amadi B, Watson A. Endomicroscopic and transcriptomic analysis of impaired barrier function and malabsorption in environmental enteropathy. PLoS Negl Trop Dis 2016; 10: e0004600.

20 Syed S, Dinallo V, Iqbal N, Di Iorio L, Di Fusco D, Guleria S, Amadi BC, Sadiq K, Moskaluk C, Ali A, Kelly P, Monteleone G. High SMAD7 and p-SMAD2,3 expression is associated with environmental enteropathy in children. *PLoS Negl Trop Dis* 2018; **12**: e0006224. doi: 10.1371/journal.pntd.0006224.

21 Attia S, Versloot CJ, Voskuijl W, van Vliet SJ, Di Giovanni V, Zhang L, Richardson S, Bourdon C, Netea MG, Berkley JA, van Rheenen PF, Bandsma RH. Mortality in children with complicated severe acute malnutrition is related to intestinal and systemic inflammation: an observational cohort study. Am J Clin Nutr 2016; 104:1441–9.

22 Picot J, et al. The effectiveness of interventions to treat severe acute malnutrition in young children: a systematic review. Health Tech Assessment 2012; 16: 19.

23 Bwakura-Dangarembizi M, Amadi B, Bourke CD, Robertson RC, Mwapenya B, Chandwe K, Kapoma C, Chifunda K, Majo F, Ngosa D, Chakara P, Chulu N, Masimba F, Mapurisa I, Besa E, Mutasa K, Mwakamui S, Runodamoto T, Humphrey JH, Ntozini R, Wells J, Manges AR, Swann J, Walker AS, Nathoo KJ, Kelly P, Prendergast AJ. Health Outcomes, Pathogenesis and Epidemiology of Severe Acute Malnutrition (HOPE-SAM): rationale and methods of a longitudinal observational study. *BMJ Open*; in press.

For peer review only - http://bmjopen.bmj.com/site/about/guidelines.xhtml

24 Marchbank T, Davison G, Oakes JR, Ghatei MA, Patterson M, Moyer MP, Playford RJ. The nutriceutical bovine colostrum truncates the increase in gut permeability caused by heavy exercise in athletes. Am J Physiol Gastrointest Liver Physiol 2011; 300: G477-84.

25 Bode L. The functional biology of human milk oligosaccharides. Early Hum Dev 2015; 91: 619-22.

26 Bode L, Salvestrini C, Park PW, Li JP, Esko JD, Yamaguchi Y, Murch S, Freeze HH. Heparan sulfate and syndecan-1 are essential in maintaining murine and human intestinal epithelial barrier function. J Clin Invest 2008; 118: 229-238.

27 Salvatore S, Heuschkel R, Tomlin S, Davies SE, Edwards S, Walker-Smith JA, French I, Murch SH. A pilot study of N-acetyl glucosamine, a nutritional substrate for glycosaminoglycan synthesis, in paediatric chronic inflammatory bowel disease. Aliment Pharmacol Ther 2000; 14: 1567-1579.

28 Jeppesen PB, Pertkiewicz M, Messing B, Iyer K, Seidner DL, O'Keefe SJ, Forbes A, Heinze H, Joelsson B. Teduglutide reduces need for parenteral support among patients with short bowel syndrome with intestinal failure. Gastroenterol 2012; 143: 1473-1481.

29 Carter BA, Cohran VC, Cole CR, Corkins MR, Dimmitt RA, Duggan C, Hill S, Horslen S, Lim JD, Mercer DF, Merritt RJ, Nichol PF, Sigurdsson L, Teitelbaum DH, Thompson J, Vanderpool C, Vaughan JF, Li B, Youssef NN, Venick RS, Kocoshis SA. Outcomes from a 12-week, open-label, multicenter clinical trial of teduglutide in pediatric short bowel syndrome. J Pediatr. 2017; 181: 102-111.e5. doi: 10.1016/j.jpeds.2016.10.027.

30 Veitch AM, Kelly P, Zulu I, Segal I, Farthing MJG. Tropical enteropathy: a T cell mediated crypt hyperplastic enteropathy. Eur J Gastroenterol Hepatol 2001;13:1175-81.

31 Jones KD, Hünten-Kirsch B, Laving AM, Munyi CW, Ngari M, Mikusa J, Mulongo MM, Odera D, Nassir HS, Timbwa M, Owino M, Fegan G, Murch SH, Sullivan PB, Warner JO, Berkley JA. Mesalazine in the initial management of severely acutely malnourished children with environmental enteric dysfunction: a pilot randomized controlled trial. BMC Med. 2014;12:133.

32 WHO Guideline: Updates on the management of severe acute malnutrition in infants and children. Geneva: World Health Organization; 2013.

33 Kosek M, Haque R, Lima A, Babji S, Shrestha S, Qureshi S, Amidou S, Mduma E, Lee G, Yori PP, Guerrant RL, Bhutta Z, Mason C, Kang G, Kabir M, Amour C, Bessong P, Turab A, Seidman J, Olortegui MP, Quetz J, Lang D, Gratz J, Miller M, Gottlieb M. Fecal markers of intestinal inflammation and permeability associated with the subsequent acquisition of linear growth deficits in infants. Am J Trop Med Hyg 2013; 88: 390-6. doi: 10.4269/ajtmh.2012.12-0549. E

For peer review only - http://bmjopen.bmj.com/site/about/guidelines.xhtml

34 Altman DG, Royston P. The cost of dichotomising continuous variables. BMJ 2006; 332: 1080.

35 Wason JMS, Seaman SR. Using continuous data on tumour measurements to improve inference in phase II cancer studies. Statistics in Medicine 2013; 32: 4639-4650.

36 Wason JM, Stecher L, Mander AP. Correcting for multiple-testing in multi-arm trials: is it necessary and is it done? Trials 2014; 15: 364.

37 Zulu J, Mwanza-Lisulo M, Besa E, Kaonga P, Chisenga CC, Chomba M, Simuyandi M, Banda R, Kelly P. Improving validity of informed consent for biomedical research in Zambia using a laboratory exposure intervention. PLOS One 2014; 9: e108305. doi: 10.1371/journal.pone.0108305.

38 Heikens GT, Bunn J, Amadi B, Manary M, Chhagan M, Berkley JA, Rollins N, Kelly P, Adamczick C, Maitland K, Tomkins A. Case management of HIV-infected, severely malnourished children in sub-Saharan Africa: challenges in the area if highest prevalence. Lancet 2008; 371: 1305-1307.

For peer review only

Table 1 Investigational medicinal products

Group	C	N	T	B	S
IMP	Colostrum	N-acetyl glucosamine	Teduglutide	Budesonide	Standard care
Presentation	Powder in capsules	Powder in capsules	Ampoules	Liquid	-
Number of patients	45	45	45	45	45
Dose:					
days 1-7	1.5g tds	300mg tds	0.05 mg/kg daily	1mg tds	-
days 8-11	1.5g tds	600mg tds	0.05 mg/kg daily	1mg bd	
days 12-14	1.5g tds	600mg tds	0.05 mg/kg daily	0.5mg bd	-
No of capsules or ampoules	1,470	2,205	630	1,806	-

All IMPs are to be given for 14 days

For peer review only

For peer review only - http://bmjopen.bmj.com/site/about/guidelines.xhtml

Table 2 Endpoints to be measured in the TAME trial

**Primary endpoint**	Units	How measured
Faecal α1-antitrypsin, myeloperoxidase and neopterin concentrations	mg/g, µg/ml, µmol/l	ELISA
**Secondary endpoints**		
Lactulose:rhamnose ratio		HPLC
Plasma intestinal fatty acid binding protein (FABP) concentration	ng/ml	ELISA
Plasma lipopolysaccharide (LPS)	EU/ml	Limulus amoebacyte lysate (LAL)
Plasma LPS binding protein (LBP)	ng/ml	ELISA
Plasma soluble CD14 and CD163	µg/ml	ELISA
Plasma C-reactive protein (CRP)	mg/l	ELISA
Plasma albumin	g/l	ELISA
Stool REG-1β	ng/g	ELISA
Mortality by day 14 and day 28		Clinical assessment
Change in anthropometric measurements (weight, mid upper arm circumference, weight-for-height) between baseline and day 14, and baseline and day 28		Weight and length measurements
Resolution of oedema between baseline and day 14		Clinical assessment
Adverse events between baseline and day 14		Clinical assessment
Serious adverse events between baseline and day 14		Clinical assessment
Days with diarrhoea (three or more loose or watery stools in 24 hours) between baseline and day 14		Clinical assessment
Days with fever (two or more recordings of core temperature of 37.8^{o}C or higher in		Clinical assessment

For peer review only - http://bmjopen.bmj.com/site/about/guidelines.xhtml

any 24 h period) between baseline and day 14		
Hormones: GLP-2, IGF-1 and IGFBP3	ng/ml	ELISA
Morphometric measures on biopsy specimens collected in Zambia only: villus height, crypt depth, villus width, epithelial surface perimeter	µm	Microscopy
Mucosal inflammatory scores	Ordinal scale	Microscopy
Mucosal leakiness (measured in Zambia only) by observation of plumes and luminal fluorescein during confocal laser endomicroscopy at day 14 (range 14-18 days)	Proportion	Confocal laser endomicroscopy videos

For peer review only

Figure legends

Figure 1 Flowchart of recruitment into the TAME trial

For peer review only

For peer review only - http://bmjopen.bmj.com/site/about/guidelines.xhtml

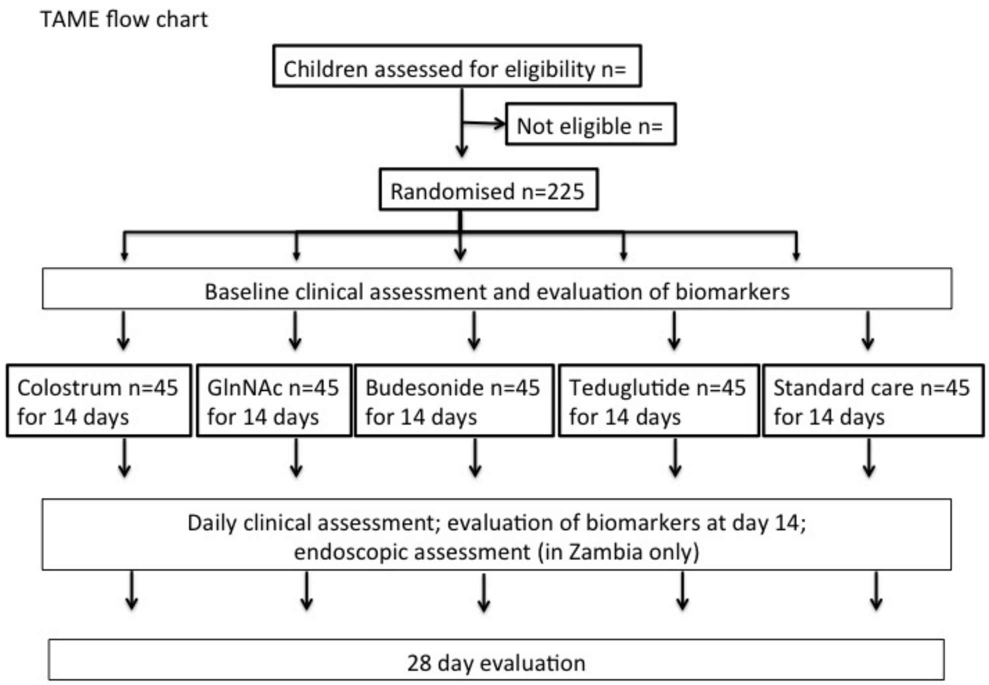

Figure 1 Trial flow diagram

254x190mm (72 x 72 DPI)

BMJ Open

The TAME trial: a multi-arm phase 2 randomised trial of four novel interventions for malnutrition enteropathy in Zambia and Zimbabwe; a study protocol

Journal:	*BMJ Open*
Manuscript ID	bmjopen-2018-027548.R1
Article Type:	Protocol
Date Submitted by the Author:	25-Jan-2019
Complete List of Authors:	Kelly, Paul; Barts and The London School of Medicine<br>Bell, Lauren; London School of Hygiene and Tropical Medicine<br>Amadi, Beatrice; 2Tropical Gastroenterology and Nutrition Group, University of Zambia, Lusaka, Zambia<br>Bwakura, Mutsa<br>VanBuskirk, Kelley<br>Chandwe, Kanta; Tropical Gastroenterology and Nutrition Group, University of Zambia, Lusaka, Zambia<br>Chipunza, Miyoba; Tropical Gastroenterology & Nutrition Group<br>Ngosa, Deophine; Tropical Gastroenterology and Nutrition Group, University of Zambia, Lusaka, Zambia<br>Chulu, Nivea; 2Tropical Gastroenterology and Nutrition Group, University of Zambia, Lusaka, Zambia<br>Hill, Susan; Great Ormond Street Hospital For Children NHS Trust,<br>Murch, Simon ; Warwick Medical School, University of Warwick, Division of Metabolic and Vascular Health<br>Playford, Raymond; University of Plymouth School of Biological Sciences<br>Prendergast, Andrew ; Queen Mary University of London,
**Primary Subject Heading**:	Nutrition and metabolism
Secondary Subject Heading:	Gastroenterology and hepatology, Global health, Paediatrics
Keywords:	Paediatric gastroenterology < GASTROENTEROLOGY, Tropical medicine < INFECTIOUS DISEASES, NUTRITION & DIETETICS, Paediatric gastroenterology < PAEDIATRICS, Clinical trials < THERAPEUTICS

SCHOLARONE™
Manuscripts

The TAME trial: a multi-arm phase 2 randomised trial of four novel interventions for malnutrition enteropathy in Zambia and Zimbabwe; a study protocol

Paul Kelly[1,2,3], Lauren Bell[3], Beatrice Amadi[2], Mutsa Bwakura-Dangarembizi[4,5], Kelley VanBuskirk[2], Kanta Chandwe[2], Miyoba Chipunza[2], Deophine Ngosa[2], Nivea Chulu[2], Susan Hill[6], Simon Murch[7], Raymond Playford[1], Andrew J Prendergast[1,5]

[1]Barts & The London School of Medicine, Queen Mary University of London, London, UK; [2]University of Zambia School of Medicine, Lusaka, Zambia; [3]London School of Hygiene and Tropical Medicine, London, UK; [4]Department of Paediatrics and Child Health, College of Health Sciences, University of Zimbabwe, Harare, Zimbabwe; [5]Zvitambo Institute for Maternal and Child Health Research, Harare, Zimbabwe; [6]Great Ormond Street Hospital, London, UK; [7]University Hospital Coventry & Warwickshire, Coventry, UK.

Corresponding author:

Professor Paul Kelly MD FRCP

Blizard Institute

Barts & The London School of Medicine

Turner Street

London E1 2AD

UK

tel: 020 7882 2643

email: m.p.kelly@qmul.ac.uk

For peer review only - http://bmjopen.bmj.com/site/about/guidelines.xhtml

ABSTRACT

Introduction

Severe acute malnutrition (SAM) in children in many countries still carries unacceptably high mortality, especially when complicated by secondary infection or metabolic derangements. New therapies are urgently needed and we have identified mucosal healing in the intestine as a potential target for novel treatment approaches.

Methods and analysis

The TAME trial (therapeutic approaches for malnutrition enteropathy) will evaluate four novel treatments in an efficient multi-arm single-blind phase II design. In three hospitals in Zambia and Zimbabwe, 225 children with SAM will be randomised to one of these treatments or to standard care, once their inpatient treatment has reached the point of transition from stabilisation to increased nutritional intake. The four interventions are budesonide, bovine colostrum or N-acetyl glucosamine given orally or via nasogastric tube, or teduglutide given by subcutaneous injection. The primary endpoint will be a composite score of faecal inflammatory markers, and a range of secondary endpoints include clinical and laboratory endpoints. Treatments will be given daily for 14 days, and evaluation of the major endpoints will be at 14-18 days, with a final clinical evaluation at 28 days. In a subset of children in Zambia, endoscopic biopsies will be used to evaluate the effect of interventions in detail.

Ethics and dissemination

The study has been approved by the University of Zambia Biomedical Research Ethics Committee (006-09-17, dated 9th July, 2018), and other local and international institutional review boards in Zambia, Zimbabwe and the UK. Caregivers will provide written informed consent for each participant. Findings will be disseminated through peer-reviewed journals, conference presentations and to caregivers at face-to-face meetings.

Registration

The trial is registered at https://clinicaltrials.gov/ct2/show/NCT03716115. Version 0.8.1 (31st October 2018) used in the preparation of this manuscript.

ARTICLE SUMMARY; strengths and limitations of the trial

Strengths

- Efficient multi-arm trial design
- Evaluation of potential efficacy of four novel interventions for a life-threatening disorder of great importance for global health
- Measurement of a broad range of biomarkers and measures of mucosal pathology

For peer review only - http://bmjopen.bmj.com/site/about/guidelines.xhtml

Potential limitations

- Only 14 days of treatment will be tested; although longer treatment may be effective, the greatest need is for treatments which confer benefit in the period of highest mortality
- There is no consensus on optimal markers of mucosal healing, hence the need for a wide range of endpoints.

For peer review only

For peer review only - http://bmjopen.bmj.com/site/about/guidelines.xhtml

BACKGROUND AND RATIONALE

Nutritional disorders are glaring examples of health inequalities between high- and low-income countries, and within low-income countries. Malnutrition underlies almost half of all child deaths globally and therefore contributes enormously to the unacceptably high under-5 mortality rates in these regions[1]. Chronic undernutrition, which usually manifests as stunting (poor linear growth), affects 40% and 27% of children in Zambia and Zimbabwe, respectively[2], and is associated with increased mortality[3], reduced neurodevelopmental potential and decreased long-term economic productivity[4]. Acute malnutrition usually manifests as wasting (loss of tissue), with or without oedema, and is the most conspicuous of all nutritional disorders. Severe acute malnutrition (SAM) carries the highest mortality[5], particularly if associated with medical complications. HIV has changed the epidemiology, pathogenesis and clinical presentation of SAM, and children with both conditions have a particularly high mortality[6].

Over the last two decades, three key developments in the approach to treatment have improved the outcomes of SAM: standardised management protocols, ready to use therapeutic food (RUTF), and community management of acute malnutrition (CMAM), which enables identification and management of children without medical complications of SAM[7,8]. However, severely malnourished children with medical complications still require hospitalisation,[9] and often fail to respond to treatment[10]. Inpatient mortality among children with complicated SAM remains up to 38%[5,11,12,13]. Even after discharge, children have a poor prognosis, with up to 42% mortality documented over the subsequent year[6]. In our experience, it is a subgroup of children with SAM and acute or persistent diarrhoea who pose the most difficult management challenges, although the vast majority of children with SAM and/or stunting have a degree of enteropathy[11,14,15].

Recent studies[16,17] have taught us a great deal about the contribution of enteric infections to dysfunction of the small intestine in malnourished children. A high pathogen burden causes damage to the mucosa which exacerbates nutritional impairment and leads to further susceptibility to infection and impaired epithelial regeneration, in a cyclical process first described in the 1970s[18,19]. This mucosal damage in SAM we refer to as *malnutrition enteropathy*, which is characterised by multiple epithelial breaches, microbial translocation from the gut lumen to the systemic circulation, and systemic inflammation[20]. Epithelial breaches are present in histological sections, seen in haematoxylin/eosin-stained sections and using immunofluorescence for claudin 4 and E-cadherin[20,21]. In parallel studies in adults, we demonstrated that these lesions occur *in vivo* using confocal laser endomicroscopy[21]. We have also identified a consistent pattern of blunted

For peer review only - http://bmjopen.bmj.com/site/about/guidelines.xhtml

epithelial repair, with reduced glucagon-like peptide 2 (GLP2) in serum, reduced trefoil factor 3 in duodenal aspirates[21], and a strong transcriptomic signature of impaired mucosal defence. We also observed two further immunological abnormalities: low-level false positive coeliac-like autoantibodies[20], and upregulation of SMAD7 somewhat similar to the pattern seen in Crohn's disease[22]. We have previously reported that markers of microbial translocation and systemic inflammation were dramatically increased in children with SAM in Zambia compared to healthy controls[20]. Together, these abnormalities indicate there is substantial structural and functional damage to the small intestine[12], and that this primary gut pathology is associated with systemic sequelae. Importantly, biomarkers of these processes have been associated with mortality among children hospitalized with complicated SAM[12].

Current treatment guidelines for SAM are not well supported by an evidence base, and there is a dearth of clinical trial data; in particular, there are no specific interventions to target enteropathy in SAM[5]. In a systematic review[23], only three trials were found which inform management of SAM and persistent diarrhoea, and no trials dealing with the HIV-infected child. We therefore believe that novel therapeutic approaches are urgently needed, and that a series of small phase 2 trials will help guide development of a new generation of treatments. These trials should focus on repairing damage to the small intestinal mucosa, as we now have substantial evidence that this plays a central role in the genesis of systemic inflammation, bacterial translocation and sepsis, though it is possible that it could be a consequence of inflammation. We propose evaluation of four new interventions: bovine colostrum, N-acetylglucosamine, teduglutide and budesonide.

OBJECTIVES

To determine if four new interventions (bovine colostrum, N-acetylglucosamine, teduglutide and budesonide) can ameliorate malnutrition enteropathy in children with severe acute malnutrition in Zambia or Zimbabwe. It is hoped that one or more of these interventions may be sufficiently promising to take through into phase 3 trials.

METHODS AND ANALYSIS

Trial design

There is emerging awareness that multi-arm Phase 2 clinical trials offer a more efficient approach to identification of new therapies than parallel conventional trial designs, in which one agent is evaluated against a control. Our recent work on the pathophysiology of malnutrition enteropathy suggests that mucosal healing may be central to reducing mortality, but we do not yet know the

For peer review only - http://bmjopen.bmj.com/site/about/guidelines.xhtml

For peer review only

efficacy of several plausible therapeutic approaches. In order to evaluate these four new potential therapies efficiently and quickly, they will be compared side-by-side using biomarkers of pathophysiology as endpoints. This design has the following advantages:

i. The four novel therapies will each be evaluated against standard care;

ii. Allocation to all five treatment groups will run in parallel, so that all children will be randomised to any of the five groups at any point in time to avoid biases due to seasonal variation;

iii. The trial endpoints will evaluate a range of biomarkers that capture different domains of malnutrition enteropathy, to allow a comprehensive non-invasive assessment of mucosal healing;

iv. A subgroup of children (those recruited in Lusaka) will have small intestinal endoscopy in a unit with a track record of safety in children.

v. Endoscopic biopsy will provide evaluation of healing and ensure that the selected therapy does not induce unwanted immune or other effects;

vi. The overall trial design provides an efficient way of identifying one or more candidates for a future Phase 3 trial.

Study setting and participant recruitment

The study will be conducted in hospitals in Lusaka, Zambia, and Harare, Zimbabwe, which have experience of several previous studies of severe acute malnutrition[11,20,24]. Children hospitalised with SAM will be eligible for inclusion if they are aged 6-59 months, of either sex, are an inpatient in one of the children's wards of the participating hospitals, are within 72 hours of initiation of transition (from F75 feed to F100 or RUTF), and clinically stable. SAM is defined using WHO definitions: weight-for-length/height Z-score of less than -3, or mid upper arm circumference of less than 11.5cm, and/or bilateral pedal oedema. During the Stabilization Phase of treatment of SAM, readiness for transition is determined by the ability to finish F75 feed by mouth and reduction in oedema (if patient was admitted with oedematous SAM). Such a child is deemed to be "clinically stable" and in general is alert, not requiring oxygen, not hypothermic, hypoglycaemic and is not in shock or dehydrated.

Children will be excluded if they have weight less than 5kg, a neurological disability or oro-facial abnormality which would explain or partly explain poor feeding, haemoglobin concentration 6 g/dl or less, contraindication to any of the trial treatments (e.g. allergy to cow's milk protein), or any

For peer review only - http://bmjopen.bmj.com/site/about/guidelines.xhtml

underlying condition, other than HIV, which in the opinion of the investigator would put the subject at undue risk of failing study completion or would interfere with analysis of study results. If the caregiver is unwilling to remain in hospital for the duration of the study treatment (14 days) they will not be recruited.

Interventions: investigational medicinal products

Colostrum is the first liquid secreted by the lactating breast, and breast-fed children take it for approximately the first three days of life. It is similar to breast milk, but with higher protein content. Bovine colostrum is available as a high-protein bovine colostrum powder (Neovite) for use as a health-promoting nutritional supplement. Colostrum contains nutrients, immunoglobulins and growth factors, including epidermal growth factor (EGF) and insulin-like growth factor 1 (IGF-1); it has been shown to reduce intestinal permeability in adults[25]. Bovine colostrum is potentially allergenic in theory, though there is no evidence that cow's milk products are harmful in children with SAM and the standard therapeutic feeds (F75 and F100 recommended by WHO) contain milk proteins.

N-acetyl glucosamine (GlcNAc) is a natural amino-sugar present on every cell surface. All breast-fed children consume GlcNAc in human milk throughout lactation[26]. Impaired glycosylation of glycosaminoglycans has been noted for many years in oedematous malnutrition, with reduced concentrations of glycosaminoglycans in blood, urine, kidney, brain and small intestine. Specific consequences of reduced heparan sulphate expression include gut epithelial leakiness with hypoalbuminaemia[27]. GlcNAc administration has been demonstrated to restore the intestinal epithelial charged barrier in Crohn's disease[28]. As with all sugars, GlcNAc may theoretically induce osmotic diarrhoea if not absorbed in the small intestine. Although this has not been observed clinically in animals or in older children, we will use a dose escalation schedule to minimise the chance of this happening.

Teduglutide is a long-acting form of GLP2 which has proven efficacy in intestinal failure, improving absorption and reducing the need for parenteral support[29]. GLP2 is a hormone secreted by L cells in the terminal ileum, which drives epithelial repair and mediates intestinal adaptation by increased cellular proliferation and villus hypertrophy. Teduglutide will be given by subcutaneous injection (0.05mg/kg/day) daily for 14 days. In a recently published description of tolerability of 12 weeks of teduglutide in children in the UK and the USA[30], vomiting was attributed to teduglutide in

For peer review only - http://bmjopen.bmj.com/site/about/guidelines.xhtml

10% of recipients and pyrexia in 14%. No children had treatment withdrawal because of adverse events. Three adverse events were considered of special interest (intestinal obstruction, fluid overload and biliary derangements) but none were observed.

Budesonide is a corticosteroid which reduces inflammation in the gut but is then rapidly broken down in the liver which minimises systemic effects. Budesonide is standard therapy for Crohn's disease, and can be used for refractory coeliac disease. Since malnutrition enteropathy is characterized by intestinal inflammation, with infiltration of activated T cells[31], an anti-inflammatory approach is rational. A prior trial of mesalazine in Kenya confirmed that an immuno-modulatory approach with mesalazine was safe in the setting of SAM[32], but targeting the small, rather than large, intestine with a more potent agent may be more effective. Budesonide may cause immunosuppression and other corticosteroid effects (oedema, hypertension, glucose intolerance, osteoporosis), but usually only after longer-term administration. It is the corticosteroid of choice for intestinal disorders because it causes fewer adverse events than prednisolone because of low systemic absorption and first-pass clearance by the liver. The dose will be tapered (1mg orally three times daily for 7 days, then 1mg twice daily for 4 days, then 0.5mg twice daily for 3 days) to mitigate any possible effects of adrenal suppression.

Trial procedures

The trial schema is shown in Figure 1. Children hospitalised with SAM will be enrolled from any of three hospitals (Lusaka Children's Hospital, Lusaka, Zambia; Parirenyatwa Hospital, Harare, Zimbabwe; Harare Central Hospital, Harare, Zimbabwe) once they have completed the stabilization phase of nutritional rehabilitation and are at the point of transition from F75 therapeutic milk (low-calorie feed) to ready-to-use therapeutic food (RUTF) or F100 milk (feeds richer in calories). Children will be randomised in an allocation ratio of (1:1:1:1:1) to either Colostrum, GlcNAc, Teduglutide, Budesonide or Standard Care and followed for 14 days in hospital (Table 1). All children will receive Standard Care according to WHO guidelines[33], and treatment with trial medications will commence on day 2 of transition.

Randomisation codes will be prepared in advance by the trial statistician, and treatment allocation will be revealed after enrolment by opening sealed envelopes held by the trial coordinators in each of the three study sites. Randomisation codes will be generated in permuted blocks and stratified by study site. Varying block sizes will be used; block size and distribution will not be

For peer review only - http://bmjopen.bmj.com/site/about/guidelines.xhtml

disclosed to the study clinical teams. There will be no attempt at blinding as the interventions are readily distinguishable (e.g. teduglutide is given as subcutaneous injection, so to mask all interventions would require administration of several placebo formulations). However, the primary endpoints are biomarkers so laboratory staff and data analysts will be blinded to randomised group.

Outcomes and endpoints

The primary endpoint for this trial will be measured on day 14 (allowable window 14 to 18 days) after initiating treatment by analysis of faecal biomarkers. Gut inflammation will be measured as a composite score of faecal myeloperoxidase, neopterin and alpha-1 antitrypsin, as described below. Secondary endpoints (Table 2) will be measured by daily clinical review (for clinical endpoints) during the intervention period and by collection of biological specimens on day 14 (allowable window 14 to 18 days) after initiating treatment. As endoscopic biopsies will be collected from trial participants in Lusaka, we anticipate that at least twenty biopsy scores will be available for each trial arm. Few trials have used histological endpoints in SAM, so this component of the trial is comparatively novel and therefore exploratory.

Sample size and data analysis

Kosek et al[34] examined established markers of intestinal inflammation which can be used to obtain population-based measures of intestinal injury and altered intestinal function. They developed an environmental enteropathy (EE) activity score which is a composite score of three stool markers for mucosal inflammation. The composite score is derived from measurements of faecal myeloperoxidase (MPO), neopterin (NEO) and alpha1-antitrypsin (AAT). These are stable compounds, relatively resistant to proteolysis and therefore suitable as biomarkers.

The weighted EE score is given in Equation 1:

Equation 1

EE score = 2 x (AAT category) + 2 x (MPO category) + 1 (1 x NEO category)

We will use the principle of a weighted enteropathy score as our primary outcome. We will not trichotomise the variables as Kosek et al[34] did, but keep the score as a continuous composite variable. This is because we are interested in gauging the potential magnitude of the effect these treatments have, and applying a cut-off value to a continuous composite score could mask or

For peer review only - http://bmjopen.bmj.com/site/about/guidelines.xhtml

discard information[35,36]. The modified Enteropathy score is given in Equation 2, with modification of weighting of units to bring measurements into a comparable arithmetical range:

Equation 2

EE biomarker score = 2x(AAT in mg/g) + 0.2x(MPO in µg/ml) + 1x(neopterin in µmol/l).

We will have a control arm, so can understand the effect of the treatment against a reference standard. The analysis will be per-protocol; no interim analysis is planned.

There are no available data from children with SAM to draw upon for an understanding of the baseline variance of the EE biomarker score. The primary outcome response from each patient is the EE biomarker score at 14 days (window 14-18 days) after treatment initiation, adjusted for baseline values. We have assumed that the EE biomarker score is normally distributed with a common standard deviation. To determine the sample size, we drew upon two methods to establish an effect size. We assumed the difference would be larger than the likely inherent imprecision in the measurements of each outcome, and we have defined the magnitude of the effect on a standardised scale. To detect a medium/large effect of Cohen's *d* effect size of 0.6, with 90% power and 90% confidence, and a conservative correlation between baseline and follow-up estimate of 0.5, we will need a sample size of 36 per group across 5 groups to analyse with the ANCOVA method (180 in total to be treated). We expect there to be approximately 10% loss to follow up due to deaths, and 10% loss to follow up due to other reasons, such as drug intolerance, withdrawal and missing specimens. Adjusting our sample size of 180 for 20% loss to follow-up, we therefore aim to randomise 225 patients in total (45 in each group).

We will use a mixed effect ANCOVA model to compare the environmental enteropathy activity score and secondary endpoints in each group against the control group, adjusting for several core covariates: sex (male/female); oedema (yes/no); HIV status (yes/no); diarrhoea (yes/no); breastfeeding (yes/no); baseline WLZ scores (continuous); baseline biomarker/histology scores (continuous) and trial site. Treatment effects will be deemed statistically significant if the p-value is less than or equal to 0.10 when compared to the control arm, for all 4 treatments. This less stringent cut-off has been chosen to reduce the likelihood of rejecting a potentially valuable treatment which might show benefit in a phase 3 trial. The DMC and TSC will contribute to a discussion at the end of the trial as to which, if any, of these treatment effects are most clinically significant.

For peer review only - http://bmjopen.bmj.com/site/about/guidelines.xhtml
For peer review only

We will not undertake any adjustments of the false positive (type I) error rate, as the aim of this trial is to inform the treatment development process for this population, which would lead to a Phase III trial if a degree of efficacy is observed. The general consensus is that adjustment for the type I error rate is not required in exploratory multi-arm multi-stage trials in Phase II within the treatment development framework[37].

If patients are discharged from hospital sooner than day 14, we will measure the primary endpoints as close as the discharge date as possible, and carry this observation forward as the primary outcome. Children who cannot submit a stool sample will be excluded from the primary analysis as the primary endpoint requires this.

Safety reporting

All adverse events will be reviewed for causality, expectedness and severity. Serious adverse events will be reported urgently to the Trial Management Group for evaluation, and all serious adverse reactions will be regarded as unexpected (i.e. SUSARs). There are two adverse events of special interest for teduglutide: fluid overload and intestinal obstruction, as these have been reported in adults on long-term teduglutide. Complicated SAM has an inpatient mortality rate of around 15%, so we anticipate up to 45 deaths in the trial; however, all deaths, regardless of causality, will be reported to the local ethics committees in Zimbabwe and Zambia according to local reporting policies. The Trial Management Committee (TMC), together with the DMC and trial steering committee (TSC), will review the pattern of any SAEs and SUSARs in real time, and is empowered to introduce urgent special measures if the profile of adverse events suggests this is required. The TSC and DMC are composed of statisticians and paediatricians, and independent of the TMC.

Patient and public involvement

There was no patient or public involvement in the design of this trial.

ETHICS AND DISSEMINATION

Consent

Ethical approval for the trial will be sought in each country. Trial nursing and medical staff will identify eligible children whose primary caregivers will then be approached to begin the process of

For peer review only - http://bmjopen.bmj.com/site/about/guidelines.xhtml

discussing the trial and the written, translated, information sheet in the most appropriate language. The consent process will include honest discussions of risks and benefits of the interventions, the concept of randomisation, and the purpose and intended use of samples collected, which is an important issue in this trial as the primary endpoints are all laboratory biomarkers. The consent process will continue throughout the trial, but all parents/guardians will sign to record that they have given informed consent to their child's participation, before any study procedures commence. Parents of children who cannot read will have the information sheet read out to them in the presence of family members before consent is accepted and recorded using a thumbprint. We have pioneered the use of participant visits to laboratories to facilitate valid consent[38]. This opportunity will be afforded to these parents and guardians. At least 24 hours will be available for consideration of consent; in practice, 2-3 days is the norm in Zambia and Zimbabwe because mothers/carers usually need to discuss enrolment with other family members. The window of time available for recruitment at and after transition allows for this consultation process.

Dissemination of results

Dissemination of results to participants' families will take place through caregiver results meetings. We will disseminate results within the academic institutions in which the trial will be conducted and to national health research authorities. Trial results will be published following CONSORT guidelines, and participant-level dataset made available through a data repository. Improvements in care will ultimately occur through change in evidence-based practice. By collaborating with colleagues on design of a future interventional trial based on our results, and by sharing specimens with other research groups, we intend to push forward the research agenda for children with SAM[39]. We will also use this trial as an opportunity to increase awareness in the UK of the complexity of malnutrition. The Centre of the Cell (www.centreofthecell.org) at Queen Mary University of London is a unique, state-of-the-art science education centre with extensive experience of translating complex medical research into innovative science communication tools. We are currently working with CoC to develop a game to explain the causes of undernutrition, including the role of the gut in nutrition, with endoscopy footage, animations and interactive gameplay . The game will be reformatted for tablets for use in Harare and Lusaka. The production of multiple formats of the tool will facilitate adults and children in the UK and Africa to access this resource and learn about current research on SAM.

For peer review only - http://bmjopen.bmj.com/site/about/guidelines.xhtml

Time frame and study status

The trial has been granted ethical approval by the University of Zambia Biomedical Research Ethics Committee (006-09-17, dated 9th July, 2018) and applications have been submitted for ethics and pharmaceutical regulatory approval in Zambia and Zimbabwe. Once recruitment begins, the trial is anticipated to take two years.

DISCUSSION

In the belief that reducing mortality from SAM will require novel therapeutic approaches, and recognising abundant evidence of impaired intestinal function, we have identified mucosal healing in the small intestine as a target for intervention. The TAME trial will employ a multi-arm design to identify one or more of four interventions for taking forward into phase 3 trials. The strengths of this trial include an efficient design, which will evaluate four novel interventions against standard care, and very extensive assessment of mucosal healing and biomarkers to provide the greatest chance of observing potential benefit. Limitations of the trial include the short duration of treatment, ongoing uncertainty about the optimal selection of biomarkers for accurate representation of intestinal absorptive and barrier function (calprotectin is not included in the primary endpoint, for example), and the complexity of data interpretation in this group of children with serious morbidity and high predicted mortality. To ease with identification of adverse events we considered it prudent to delay recruitment until the child has stabilised. However this also means that an important, possibly critical, window for therapy during the first few days of admission may be missed. Hopefully this can be addressed in future studies.

We have considered carefully the issue of duration of treatment. Inpatient treatment of SAM lasts usually between 1-2 weeks and it is in this narrow time window that most, though not all[6], of the mortality occurs. If novel therapies for reduction of mortality in SAM are to be useful at scale, they must confer benefit quickly, during the 1-2 week period immediately after admission. While it would be of great interest to evaluate more extended periods of therapy, the most urgent need is for new treatments in this short period of maximum morbidity and mortality. In future trials of any promising interventions, the timing of introduction of novel therapies would be an explicit research question, as they may also be of value earlier in treatment, or continued for a longer duration, including after discharge. However, for this phase II trial, we will only enrol children once they are clinically stable, at the transition phase, and provide treatment over 14 days during the typical period of hospitalisation.

For peer review only - http://bmjopen.bmj.com/site/about/guidelines.xhtml

Two of these agents (bovine colostrum and N-acetyl glucosamine) are nutraceuticals, food-derived and generally regarded as safe. While this is very reassuring about the low probability of adverse events, it is hard to quantify that probability with any accuracy. Regulatory agencies are more familiar with IMPs which have well-described adverse event profiles based on careful phase 1 trials, so paradoxically the level of scrutiny of any potential adverse events in the TAME trial will be higher than if full SmPCs were publically available, despite the expected low risk of significant AEs.

Teduglutide has not previously been used for treating enteropathy in malnutrition, but there is growing evidence of its benefit in intestinal failure[29]. Children with SAM have evidence of a hyperplastic enteropathy which is likely to lead to impaired absorption of nutrients. Teduglutide is a GLP2 analogue which can enhance intestinal adaptation and reduce requirements for parenteral nutrition in patient receiving it. It may overcome some of the impaired absorption in severe enteropathies, which may enhance nutrient uptake in SAM. However there are possible adverse effects, including increased salt and water retention which (although not reported in previous studies in children[30]) will be an adverse event of special interest in TAME. Intestinal obstruction has also been reported in adults as a consequence of mucosal hypertrophy; it too will be an adverse event of special interest.

In summary, we propose a phase 2 trial designed to target malnutrition enteropathy, which we believe is central to the pathogenesis of SAM, prevents recovery, and is not currently addressed by current interventions. Identification of promising novel treatment approaches would provide a strong rationale for a larger efficacy trial to reduce the unacceptably high morbidity, mortality and relapse among children with complicated SAM.

For peer review only

For peer review only - http://bmjopen.bmj.com/site/about/guidelines.xhtml

Authors' contributions

PK, AJP, BA and MB-D originated the trial idea and obtained funding. KvB and LB provided statistical expertise. MC, DN, and MC collected preliminary data for sample size calculations. KC, RP, SM and SH provided specific input into design of specific elements of the trial, particularly the principal interventions. All authors contributed to and approved the final manuscript.

Funding statement

The TAME trial is funded by the Medical Research Council (MR/P024033/1). AJP and MB-D are funded by the Wellcome Trust (grants 093768/Z/15/Z and 107634/Z/15/Z, respectively). These funding agencies have had no role in the preparation of this manuscript.

Competing interests

The authors declare that they have no competing interests

Word count

3,891

Trial Sponsor

Queen Mary, University of London

For peer review only

References

1 Black RE, Victora CG, Walker SP, Bhutta ZA, Christian P, de Onis M, Ezzati M, Grantham-McGregor S, Katz J, Martorell R, Uauy R. Maternal and child undernutrition and overweight in low-income and middle-income countries. Lancet. 2013; 382: 427-51.

2 Zambia Demographic and Health Survey 2013-14; Zimbabwe Demographic and Health Survey 2015. Rockville, Maryland, USA: Central Statistical Office, Ministry of Health, and ICF International.

3 Olofin I, McDonald CM, Ezzati M, Flaxman S, Black RE, Fawzi WW, Caulfield LE, Danaei G. Associations of suboptimal growth with all-cause and cause-specific mortality in children under five years: a pooled analysis of ten prospective studies. PLoS One 2013 8: e64636.

4 Victora CG, Adair L, Fall C, Hallal PC, Martorell R, Richter L, Sachdev HS. Maternal and child undernutrition: consequences for adult health and human capital. Lancet 2008; 371: 340–357.

5 Lenters LM, Wazny K, Webb P, Ahmed T, Bhutta ZA. Treatment of severe and moderate acute malnutrition in low- and middle-income settings: a systematic review, meta-analysis and Delphi process. BMC Public Health. 2013; 13 Suppl 3: S23.

6 Kerac M, Bunn J, Chagaluka G, Bahwere P, Tomkins A, Collins S, Seal A. Follow-up of post-discharge growth and mortality after treatment for severe acute malnutrition (FuSAM study): a prospective cohort study. PLoS One 2014; 9: e96030.

7 Collins S, Dent N, Binns P, Bahwere P, Sadler K, Hallam A. Management of severe acute malnutrition in children. Lancet 2006; 368: 1992-2000.

8 Amadi B, Imasiku M, Sakala M, Banda R, Kelly P. Integration of HIV care into community management of acute childhood malnutrition permits good outcomes: retrospective analysis of three years of a programme in Lusaka. PLoS One 2016; 11: e0149218.

9 WHO Guideline: Updates on the management of severe acute malnutrition in infants and children. Geneva: World Health Organization; 2013.

10 Isanaka S, Langendorf C, Berthé F, Gnegne S, Li N, Ousmane N, Harouna S, Hassane H, Schaefer M, Adehossi E, Grais RF. Routine Amoxicillin for Uncomplicated Severe Acute Malnutrition in Children. N Engl J Med 2016; 374: 444-53.

11 Amadi BC, Kelly P, Mwiya M, Mulwazi E, Sianongo S, Changwe F, Thomson M, Hachungula J, Watuka A, Walker-Smith JA, Chintu C. Intestinal and systemic infection, HIV and mortality in Zambian children with persistent diarrhoea and malnutrition. J Ped Gastroenterol Nutr 2001; 32: 550-554.

12 Attia S, Versloot CJ, Voskuijl W, van Vliet SJ, Di Giovanni V, Zhang L, Richardson S, Bourdon C, Netea MG, Berkley JA, van Rheenen PF, Bandsma RH. Mortality in children with complicated severe acute malnutrition is related to intestinal and systemic inflammation: an observational

For peer review only

cohort study. Am J Clin Nutr 2016; 104:1441–9.

13 Bartels RH, Bourdon C, Potani I, Mhango B, van den Brink DA, Mponda JS, Muller Kobold AC, Bandsma RH, Boele van Hensbroek M, Voskuijl WP. Pancreatic Enzyme Replacement Therapy in Children with Severe Acute Malnutrition: A Randomized Controlled Trial. J Pediatr. 2017; 190: 85-92.e2.

14 Prendergast A, Kelly P. Interactions between intestinal pathogens, enteropathy and malnutrition in developing countries. Curr Op Infect Dis 2015; 29; 229-236.

15 Trehan I, Kelly P, Shaikh N, Manary MJ. New insights into Environmental Enteric Dysfunction. Arch Dis Child 2016; 101:741-4.

16 Kotloff KL, et al. Burden and aetiology of diarrhoeal disease in infants and young children in developing countries (the Global Enteric Multicenter Study, GEMS): a prospective, case-control study. Lancet 2013; 382: 209–22.

17 Platts-Mills JA, et al. Pathogen-specific burdens of community diarrhoea in developing countries: a multisite birth cohort study (MAL-ED). Lancet Glob Health 2015; 3: e564–75.

18 Scrimshaw, NS, et al. Monogr Ser World Health Organ, 1968. 57: 3-329.

19 Mata LJ. The Children of Santa Maria Cauqué: A Prospective Field Study of Health and Growth (International nutrition policy series) MIT Press: 1978.

20 Amadi B, Besa E, Zyambo K, Kaonga P, Louis-Auguste J, Chandwe K, Tarr PI, Denno DM, Nataro JP, Faubion W, Sailer A, Yeruva S, Brantner T, Murray JA, Prendergast AJ, Turner JR, Kelly P. Impaired barrier function and autoantibody generation in malnutrition enteropathy in Zambia. EBiomedicine 2017; 22: 191-199.

21 Kelly P, Besa E, Zyambo K, Louis-Auguste J, Lees J, Banda T, Soko R, Banda R, Amadi B, Watson A. Endomicroscopic and transcriptomic analysis of impaired barrier function and malabsorption in environmental enteropathy. PLoS Negl Trop Dis 2016; 10: e0004600.

22 Syed S, Dinallo V, Iqbal N, Di Iorio L, Di Fusco D, Guleria S, Amadi BC, Sadiq K, Moskaluk C, Ali A, Kelly P, Monteleone G. High SMAD7 and p-SMAD2,3 expression is associated with environmental enteropathy in children. *PLoS Negl Trop Dis* 2018; **12**: e0006224. doi: 10.1371/journal.pntd.0006224.

23 Picot J, et al. The effectiveness of interventions to treat severe acute malnutrition in young children: a systematic review. Health Tech Assessment 2012; 16: 19.

24 Bwakura-Dangarembizi M, Amadi B, Bourke CD, Robertson RC, Mwapenya B, Chandwe K, Kapoma C, Chifunda K, Majo F, Ngosa D, Chakara P, Chulu N, Masimba F, Mapurisa I, Besa E, Mutasa K, Mwakamui S, Runodamoto T, Humphrey JH, Ntozini R, Wells J, Manges AR, Swann J, Walker AS, Nathoo KJ, Kelly P, Prendergast AJ. Health Outcomes, Pathogenesis and Epidemiology of Severe

For peer review only - http://bmjopen.bmj.com/site/about/guidelines.xhtml

Acute Malnutrition (HOPE-SAM): rationale and methods of a longitudinal observational study. *BMJ Open*; in press.

25 Marchbank T, Davison G, Oakes JR, Ghatei MA, Patterson M, Moyer MP, Playford RJ. The nutriceutical bovine colostrum truncates the increase in gut permeability caused by heavy exercise in athletes. Am J Physiol Gastrointest Liver Physiol 2011; 300: G477-84.

26 Bode L. The functional biology of human milk oligosaccharides. Early Hum Dev 2015; 91: 619-22.

27 Bode L, Salvestrini C, Park PW, Li JP, Esko JD, Yamaguchi Y, Murch S, Freeze HH. Heparan sulfate and syndecan-1 are essential in maintaining murine and human intestinal epithelial barrier function. J Clin Invest 2008; 118: 229-238.

28 Salvatore S, Heuschkel R, Tomlin S, Davies SE, Edwards S, Walker-Smith JA, French I, Murch SH. A pilot study of N-acetyl glucosamine, a nutritional substrate for glycosaminoglycan synthesis, in paediatric chronic inflammatory bowel disease. Aliment Pharmacol Ther 2000; 14: 1567-1579.

29 Jeppesen PB, Pertkiewicz M, Messing B, Iyer K, Seidner DL, O'Keefe SJ, Forbes A, Heinze H, Joelsson B. Teduglutide reduces need for parenteral support among patients with short bowel syndrome with intestinal failure. Gastroenterol 2012; 143: 1473-1481.

30 Carter BA, Cohran VC, Cole CR, Corkins MR, Dimmitt RA, Duggan C, Hill S, Horslen S, Lim JD, Mercer DF, Merritt RJ, Nichol PF, Sigurdsson L, Teitelbaum DH, Thompson J, Vanderpool C, Vaughan JF, Li B, Youssef NN, Venick RS, Kocoshis SA. Outcomes from a 12-week, open-label, multicenter clinical trial of teduglutide in pediatric short bowel syndrome. J Pediatr. 2017; 181: 102-111.e5. doi: 10.1016/j.jpeds.2016.10.027.

31 Veitch AM, Kelly P, Zulu I, Segal I, Farthing MJG. Tropical enteropathy: a T cell mediated crypt hyperplastic enteropathy. Eur J Gastroenterol Hepatol 2001;13:1175-81.

32 Jones KD, Hünten-Kirsch B, Laving AM, Munyi CW, Ngari M, Mikusa J, Mulongo MM, Odera D, Nassir HS, Timbwa M, Owino M, Fegan G, Murch SH, Sullivan PB, Warner JO, Berkley JA. Mesalazine in the initial management of severely acutely malnourished children with environmental enteric dysfunction: a pilot randomized controlled trial. BMC Med. 2014;12:133.

33 WHO Guideline: Updates on the management of severe acute malnutrition in infants and children. Geneva: World Health Organization; 2013.

34 Kosek M, Haque R, Lima A, Babji S, Shrestha S, Qureshi S, Amidou S, Mduma E, Lee G, Yori PP, Guerrant RL, Bhutta Z, Mason C, Kang G, Kabir M, Amour C, Bessong P, Turab A, Seidman J, Olortegui MP, Quetz J, Lang D, Gratz J, Miller M, Gottlieb M. Fecal markers of intestinal inflammation and permeability associated with the subsequent acquisition of linear growth deficits in infants. Am J Trop Med Hyg 2013; 88: 390-6. doi: 10.4269/ajtmh.2012.12-0549. E

For peer review only - http://bmjopen.bmj.com/site/about/guidelines.xhtml

35 Altman DG, Royston P. The cost of dichotomising continuous variables. BMJ 2006; 332: 1080.

36 Wason JMS, Seaman SR. Using continuous data on tumour measurements to improve inference in phase II cancer studies. Statistics in Medicine 2013; 32: 4639-4650.

37 Wason JM, Stecher L, Mander AP. Correcting for multiple-testing in multi-arm trials: is it necessary and is it done? Trials 2014; 15: 364.

38 Zulu J, Mwanza-Lisulo M, Besa E, Kaonga P, Chisenga CC, Chomba M, Simuyandi M, Banda R, Kelly P. Improving validity of informed consent for biomedical research in Zambia using a laboratory exposure intervention. PLOS One 2014; 9: e108305. doi: 10.1371/journal.pone.0108305.

39 Heikens GT, Bunn J, Amadi B, Manary M, Chhagan M, Berkley JA, Rollins N, Kelly P, Adamczick C, Maitland K, Tomkins A. Case management of HIV-infected, severely malnourished children in sub-Saharan Africa: challenges in the area if highest prevalence. Lancet 2008; 371: 1305-1307.

For peer review only

For peer review only - http://bmjopen.bmj.com/site/about/guidelines.xhtml

Table 1 Investigational medicinal products

**Group**	**C**	**N**	**T**	**B**	**S**
IMP	Colostrum	N-acetyl glucosamine	Teduglutide	Budesonide	Standard care
Presentation	Powder in capsules	Powder in capsules	Ampoules	Liquid	-
Number of patients	45	45	45	45	45
Dose:					
days 1-7	1.5g tds	300mg tds	0.05 mg/kg daily	1mg tds	-
days 8-11	1.5g tds	600mg tds	0.05 mg/kg daily	1mg bd	
days 12-14	1.5g tds	600mg tds	0.05 mg/kg daily	0.5mg bd	-
No of capsules or ampoules	1,470	2,205	630	1,806	-

All IMPs are to be given for 14 days

For peer review only

Table 2 Endpoints to be measured in the TAME trial

**Primary endpoint**	Units	How measured
Faecal α1-antitrypsin, myeloperoxidase and neopterin concentrations	mg/g, μg/ml, μmol/l	ELISA
**Secondary endpoints**		
Lactulose:rhamnose ratio		HPLC
Plasma intestinal fatty acid binding protein (FABP) concentration	ng/ml	ELISA
Plasma lipopolysaccharide (LPS)	EU/ml	Limulus amoebacyte lysate (LAL)
Plasma LPS binding protein (LBP)	ng/ml	ELISA
Plasma soluble CD14 and CD163	μg/ml	ELISA
Plasma C-reactive protein (CRP)	mg/l	ELISA
Plasma albumin	g/l	ELISA
Stool REG-1β	ng/g	ELISA
Mortality by day 14 and day 28		Clinical assessment
Change in anthropometric measurements (weight, mid upper arm circumference, weight-for-height, MUAC) between baseline and day 14, and baseline and day 28		Weight and length measurements
Resolution of oedema between baseline and day 14		Clinical assessment
Adverse events between baseline and day 14		Clinical assessment
Serious adverse events between baseline and day 14		Clinical assessment
Days with diarrhoea (three or more loose or watery stools in 24 hours) between baseline and day 14		Clinical assessment
Days with fever (two or more recordings of core temperature of 37.8^{o}C or higher in any 24 h period) between baseline and day		Clinical assessment

For peer review only

For peer review only - http://bmjopen.bmj.com/site/about/guidelines.xhtml

14		
Hormones: GLP-2, IGF-1 and IGFBP3	ng/ml	ELISA
Morphometric measures on biopsy specimens collected in Zambia only: villus height, crypt depth, villus width, epithelial surface perimeter	µm	Microscopy
Mucosal inflammatory scores	Ordinal scale	Microscopy
Mucosal leakiness (measured in Zambia only) by observation of plumes and luminal fluorescein during confocal laser endomicroscopy at day 14 (range 14-18 days)	Proportion	Confocal laser endomicroscopy videos

MUAC, mid upper arm circumference; GLP-2, glucagon-like peptide 2; IGF, insulin-like growth factor; IGFBP, IGF binding protein.

For peer review only

Figure legends

Figure 1 Flowchart of recruitment into the TAME trial

For peer review only

For peer review only - http://bmjopen.bmj.com/site/about/guidelines.xhtml

TAME flow chart

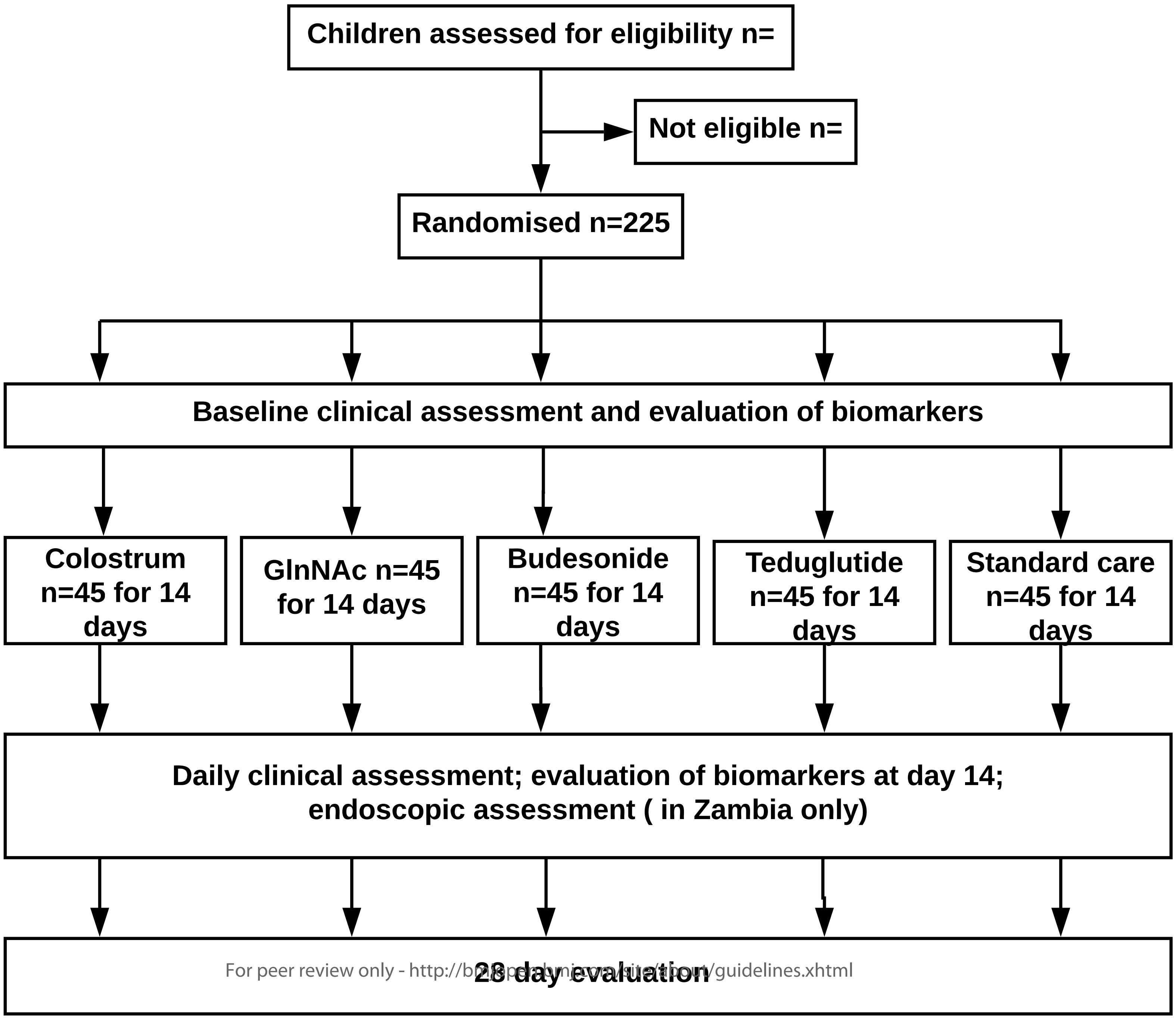

For peer review only - http://bmjopen.bmj.com/site/about/guidelines.xhtml

Therapeutic approaches to malnutrition enteropathy (TAME): phase II trials of four novel interventions in children in Zambia and Zimbabwe

Zambia

Principal Investigator for Zambia: Dr Beatrice Amadi
Chief Investigator: Professor Paul Kelly

Participant Information Sheet

We are researchers from the University of Zambia School of Medicine and Lusaka Children's Hospital. We would like to invite you and your child to be part of this research project. You should only agree that your child could take part if you want to; it is entirely up to you. If you choose your child not to take part, there will be no disadvantages. Choosing not to take part will not affect your child's treatment in any way, now or in the future. Please read or listen to the following information carefully before you decide that your child can take part. If you decide to take part, you will be asked to sign a consent form to record your agreement. You are free to withdraw at any time during your child's participation without giving a reason.

<u>Why we are doing this trial</u>

The purpose of this trial is to develop extra treatments to help in the full recovery of children with Severe Acute Malnutrition (SAM). We know that children with SAM are at increased risk of dying. We know that malnutrition causes damage to the gut (intestine), which can allow it to become 'leaky'. We believe that if this does not heal properly, children do not recover from malnutrition. We want to try giving new medicines to heal the damaged intestine.

<u>Why has my child been chosen?</u>

We have identified that your child has severe acute malnutrition with complications. We want to include your child in a trial of some new treatments.

<u>What the study involves</u>

There are four new treatments which we want to compare against the treatments we currently use. Current treatments closely follow the guidelines of the World Health Organisation, including special feeds and fluids, and antibiotics. We do not know if these new treatments are better than the treatments we already have so we need to compare them in a randomised clinical trial. This means that the choice of treatments is made randomly, by chance (like the flip of a coin). For one out of five children, the treatment will remain exactly the same care as we give now. For four out of five children, we will give all the current treatments plus a new drug. A set of envelopes has been prepared with numbers on. If you agree that your child is to be included in the trial, a study number will be given. Once that number has been given we will open the envelope with that number and see which new treatment should be given, or if your child should not be given a new treatment. It is very important that you understand these two principles of a randomised trial:

1. We genuinely do not know which treatment is best, or if they are better or worse than what we do already.

For peer review only - http://bmjopen.bmj.com/site/about/guidelines.xhtml

2. Children will be allocated a treatment by chance.

Once the envelope has been opened, we will follow the treatment course indicated on the contents of the envelope. We will not choose the treatment for your child.

The treatments

There are four possible treatments, but your child will only receive one of these at most. We do not know which one until the envelope is opened. All of these medicines need to be given for 2 weeks in hospital, not at home:

1. **Colostrum**. When mothers give birth, the first type of milk that comes from the breast is colostrum, which is a yellowy colour. This is full of protective substances for the baby and we think it also may help to heal the gut. We have obtained colostrum from cows as one of the new treatments. It is as safe as the cow's milk we all drink in our tea, but there is the small possibility that it may be too rich for babies with severe acute malnutrition and may cause or worsen diarrhoea. We will watch out for such effects. It is given by mouth or down the gastric tube.
2. **N-acetyl glucosamine (GlucNac)**. This is a sort of starch which we hope will build up the protective mucus which lines our intestines. There is the small possibility that it may be too rich for children with severe acute malnutrition and may cause or worsen diarrhoea. We will watch out for such effects. It is given by mouth or down the gastric tube.
3. **Teduglutide (TDG)**. This is a modified version of a natural hormone the body produces, which helps the gut to repair itself after injury. It has been used in children with very severe gut failure, and has helped them to absorb more nutrients from the milk they get. A recent trial in the USA and the UK shows that it is safe, but it can cause vomiting and fever in about one in ten children who receive it for 3 months. We think it is unlikely that this will occur when the treatment is given for 14 days, but we will watch out for that. It is given as an injection under the skin, once every day.
4. **Budesonide**. This is a steroid, used to help heal the gut in children with other sorts of gut disease. Steroids can have side effects, including fluid accumulation and high levels of sugar in the blood and weakened resistance to infection, but usually only when they are given for longer periods. Budesonide is designed to treat gut inflammation without being absorbed, but we will watch out for any effects. It is given by mouth or down the gastric tube.

The investigations your child will have

When your child enters the study, he/she will have a full medical assessment and examination. All these tests are completely safe. We will also ask for an additional blood sample, a stool sample, and a saliva sample, then we do a simple test which involves drinking a small amount of a sugary liquid and collecting two urine samples. To reduce the pain of taking blood, we can use a jelly on your child's skin which very much reduces the discomfort. We will then take another set of blood samples after one week; and blood, urine, saliva and stool samples after two weeks. The total volume of blood we will collect for research is limited to a safe quantity (2ml/kg body weight in total during the trial). We will carry out HIV testing as part of the tests on blood samples; the results will be completely confidential even if it is negative. All children will be monitored daily by our team of doctors and nurses for the full two weeks, and then we will ask you to come back

For peer review only - http://bmjopen.bmj.com/site/about/guidelines.xhtml

on the 28th day so that we can give your child a final growth check and ask a few questions.
In addition, between days 14 and 18 we will carry out an endoscopy. This means examining the inside of the stomach and intestine with a small flexible tube, through which we can collect tiny pieces of the lining of the intestine, each about the size of the head of a match. Although this sounds rather worrying, in fact this is quite safe because we have good facilities and a great deal of experience. The endoscopy requires giving anaesthetic injections; this is done by an anaesthetist who monitors the children very carefully.

If you would like, we would be very happy to show you the endoscopy unit and equipment at UTH so you have a better idea of what is involved. We can also show you the laboratories so you can see what we do with the samples we take, and how we analyse them.

How many children will be in the study?
The study is being conducted in Zambia and Zimbabwe. There will be 225 children in total in the study.

What we do with your child's samples
We only use the samples you provide for research. Any samples that we do not need for research will be destroyed. We have no need to use the samples you provide for any other purpose, including giving them to people who are not involved in the research. It is also against the law to do so.

We do nearly all of the research analysis in the laboratories at UTH. Some of the tests will need to be done by our collaborators in the UK or USA, so some of your child's samples may be sent there. These collaborating laboratories are not allowed to do anything with the samples that isn't to do with research, including selling them or giving them to people who aren't involved in the research. We are very strict on this and Prof Kelly can give you absolute reassurance that the samples are only used for this research. Once the research is finished they will be destroyed.

We do many different measurements on the samples you provide, and if you are interested we can demonstrate and explain some of these to you, including how the samples are stored.

What we do with the information you provide
During the research we collect information about you and your child. We store the information on computers and remove your name and your child's name and any other ways of being identified, so that there is no way of telling who the information refers to. Any and all information you give us is confidential. This means that we are not allowed to tell anyone about you or your child, even if they are friends or family.

How this study might benefit you

For peer review only - http://bmjopen.bmj.com/site/about/guidelines.xhtml

We hope that the new treatment will speed up your child's recovery from SAM. However, because these treatments are new, we do not know at this stage whether they will work and we cannot therefore guarantee any benefits for your child. Your child will continue to receive the best possible care for malnutrition as well as any new treatments we give.

We hope that our learning from this trial will benefit children with SAM in the future.

Are there any risks to participating in this study?
This is a research project, and these treatments have never before been used in children in Zambia with severe acute malnutrition. We have already explained the possible risks of these treatments, and that we have designed the doses used to minimise these risks. We have considered this carefully and done our best, but the possibility remains that some children might experience adverse reactions to these treatments. We can promise you that we will watch out carefully for such reactions and that we will provide the best possible care in the event that a reaction does occur.

What if I don't want my child to take part in the study or I want to withdraw later?
Participation in this study is voluntary. It is completely up to you whether or not your child participates, or if you join the trial but decide to withdraw later. If you decide for your child not to participate, it will not affect the treatment your child receives now or in the future. Whatever your decision, it will not affect your relationship with the staff caring for you.

Insurance and what to do if you have questions or concerns
In the unlikely event that your child is harmed as a result of this trial, Queen Mary University of London has agreed that you will be compensated, provided that, on the balance of probabilities, an injury was caused as a direct result of the intervention or procedures your child received during the course of the study. These special compensation arrangements apply where an injury is caused to your child that would not have occurred if s/he were not in the study. These arrangements do not affect your right to pursue a claim through legal action.

It is up to you to decide whether or not to take part. If you do decide to take part you will be given this information sheet to keep and be asked to sign a consent record form.

If you have any questions or concerns about the study or how it is conducted, you can contact Professor Kelly's and Dr Amadi's team in the hospital at any point

Contact details are:

Professor Paul Kelly
Endoscopy Unit, Clinic 5,
University Teaching Hospital
Lusaka
Tel 0211 252269/0955 295493
or Dr Beatrice Amadi
Ward A07
Lusaka Children's Hospital

For peer review only - http://bmjopen.bmj.com/site/about/guidelines.xhtml

Lusaka
tel: 0966 752739
If you are not happy to do this, or are not happy with the advice you get from Professor Kelly's and Dr Amadi's team, you can contact:

The University of Zambia Biomedical Research Ethics Committee,
University of Zambia School of Medicine,
Ridgeway Campus,
PO Box 50110,
Lusaka.
tel: 0211 250753 and speak to Lorna

A COPY OF THE INFORMATION SHEET AND CONSENT RECORD WILL BE GIVEN TO YOU FOR SAFE KEEPING, AND A COPY WILL BE RETAINED BY THE STUDY TEAM

For peer review only

For peer review only - http://bmjopen.bmj.com/site/about/guidelines.xhtml

Please complete this form after you have read or listened to the Information Sheet and/or listened to an explanation about the research.

Therapeutic approaches to malnutrition enteropathy (TAME)
UNZABREC reference: tbc

1. I have read or listened to the Participant Information Sheet for this study (version 0.7, dated 28th March, 2018).
2. The research trial has been explained to me. I understand what the research involves and what is required of me.
3. I understand that my child will have a blood test for HIV.
4. I understand that samples obtained from my child may be sent abroad for research purposes only, and that after use the samples will either be returned to Zambia for storage or destroyed.
5. I understand that there is no obligation for me to allow my child to participate in the research study, and that I do not have to give a reason for not participating.
6. I understand that if I decide at any time during the research that I no longer wish my child to participate, I can notify the researchers involved and be withdrawn from it immediately, without giving a reason.
7. I consent to the processing of our personal information for the purposes of this research study. I understand that such information will be treated as strictly confidential at all times and that any identifiable information about me or my child will be available only to members of the study team.

Participant's statement:
I ____ have read or listened to statements 1-7 above and I agree to allow my child ______________________________ to take part in the study.

Signed: Date:

Witnessed: Date:

Investigator's Statement:
I ____ confirm that I have carefully explained the nature, demands and any foreseeable risks of the proposed research to the family.

Signed: Date:

For peer review only - http://bmjopen.bmj.com/site/about/guidelines.xhtml

Therapeutic approaches to malnutrition enteropathy (TAME): phase II trials of four novel interventions in children in Zambia and Zimbabwe

ZIMBABWE

Principal Investigator for Zimbabwe: Dr Mutsa Bwakura-Dangarembizi
Chief Investigator: Professor Paul Kelly

Participant Information Sheet

We are researchers from the University of Zimbabwe College of Health Sciences, Harare Central Hospital and Parirenyatwa Hospital. We would like to invite you and your child to be part of this research project. You should only agree that your child could take part if you want to; it is entirely up to you. If you choose your child not to take part, there will be no disadvantages. Choosing not to take part will not affect your child's treatment in any way, now or in the future. Please read or listen to the following information carefully before you decide that your child can take part. If you decide to take part, you will be asked to sign a consent form to record your agreement. You are free to withdraw at any time during your child's participation without giving a reason.

Why we are doing this trial

The purpose of this trial is to develop extra treatments to help in the full recovery of children with Severe Acute Malnutrition (SAM). We know that children with SAM are at increased risk of dying. We know that malnutrition causes damage to the gut (intestine), which can allow it to become 'leaky'. We believe that if this does not heal properly, children do not recover from malnutrition. We want to try giving new medicines to heal the damaged intestine.

Why has my child been chosen?

We have identified that your child has severe acute malnutrition with complications. We want to include your child in a trial of some new treatments.

What the study involves

There are four new treatments which we want to compare against the treatments we currently use. Current treatments closely follow the guidelines of the World Health Organisation, including special feeds and fluids, and antibiotics. We do not know if these new treatments are better than the treatments we already have so we need to compare them in a randomised clinical trial. This means that the choice of treatments is made randomly, by chance (like the flip of a coin). For one out of five children, the treatment will remain exactly the same care as we give now. For four out of five children, we will give all the current treatments plus a new drug. A set of envelopes has been prepared with numbers on. If you agree that your child is to be included in the trial, a study number will be given. Once that number has been given we will open the envelope with that number and see which new treatment should be given, or if your child should not be given a new treatment. It is very important that you understand these two principles of a randomised trial:

1. We genuinely do not know which treatment is best, or if they are better or worse than what we do already.

For peer review only - http://bmjopen.bmj.com/site/about/guidelines.xhtml

2. Children will be allocated a treatment by chance.

Once the envelope has been opened, we will follow the treatment course indicated on the contents of the envelope. We will not choose the treatment for your child.

The treatments

There are four possible treatments, but your child will only receive one of these at most. We do not know which one until the envelope is opened. All of these medicines need to be given for 2 weeks in hospital, not at home:

1. **Colostrum**. When mothers give birth, the first type of milk that comes from the breast is colostrum, which is a yellowy colour. This is full of protective substances for the baby and we think it also may help to heal the gut. We have obtained colostrum from cows as one of the new treatments. It is as safe as the cow's milk we all drink in our tea, but there is the small possibility that it may be too rich for babies with severe acute malnutrition and may cause or worsen diarrhoea. We will watch out for such effects. It is given by mouth or down the gastric tube.
2. **N-acetyl glucosamine (GlucNac)**. This is a sort of starch which we hope will build up the protective mucus which lines our intestines. There is the small possibility that it may be too rich for children with severe acute malnutrition and may cause or worsen diarrhoea. We will watch out for such effects. It is given by mouth or down the gastric tube.
3. **Teduglutide (TDG)**. This is a modified version of a natural hormone the body produces, which helps the gut to repair itself after injury. It has been used in children with very severe gut failure, and has helped them to absorb more nutrients from the milk they get. A recent trial in the USA and the UK shows that it is safe, but it can cause vomiting and fever in about one in ten children who receive it for 3 months. We think it is unlikely that this will occur when the treatment is given for 14 days, but we will watch out for that. It is given as an injection under the skin, once every day.
4. **Budesonide**. This is a steroid, used to help heal the gut in children with other sorts of gut disease. Steroids can have side effects, including fluid accumulation and high levels of sugar in the blood and weakened resistance to infection, but usually only when they are given for longer periods. Budesonide is designed to treat gut inflammation without being absorbed, but we will watch out for any effects. It is given by mouth or down the gastric tube.

The investigations your child will have

When your child enters the study, he/she will have a full medical assessment and examination. All these tests are completely safe. We will also ask for an additional blood sample, a stool sample, and a saliva sample, then we do a simple test which involves drinking a small amount of a sugary liquid and collecting two urine samples. To reduce the pain of taking blood, we can use a jelly on your child's skin which very much reduces the discomfort. We will then take another set of blood samples after one week; and blood, urine, saliva and stool samples after two weeks. The total volume of blood we will collect for research is limited to a safe quantity (2ml/kg body weight in total during the trial). We will carry out HIV testing as part of the tests on blood samples; the results will be completely confidential even if it is negative. All children will be monitored daily by our team of doctors and nurses for the full two weeks, and then we will ask you to come back

For peer review only - http://bmjopen.bmj.com/site/about/guidelines.xhtml

on the 28th day so that we can give your child a final growth check and ask a few questions.
If you would like, we would be very happy to show you the laboratories so you can see what we do with the samples we take, and how we analyse them.

How many children will be in the study?

The study is being conducted in Zambia and Zimbabwe. There will be 225 children in total in the study.

What we do with your child's samples

We only use the samples you provide for research. Any samples that we do not need for research will be destroyed. We have no need to use the samples you provide for any other purpose, including giving them to people who are not involved in the research. It is also against the law to do so.

We do nearly all of the research analysis in the laboratories in Harare. Some of the tests will need to be done by our collaborators in the UK or USA, so some of your child's samples may be sent there. These collaborating laboratories are not allowed to do anything with the samples that isn't to do with research, including selling them or giving them to people who aren't involved in the research. We are very strict on this and Prof Kelly can give you absolute reassurance that the samples are only used for this research. Once the research is finished they will be destroyed.

We do many different measurements on the samples you provide, and if you are interested we can demonstrate and explain some of these to you, including how the samples are stored.

What we do with the information you provide

During the research we collect information about you and your child. We store the information on computers and remove your name and your child's name and any other ways of being identified, so that there is no way of telling who the information refers to. Any and all information you give us is confidential. This means that we are not allowed to tell anyone about you or your child, even if they are friends or family.

How this study might benefit you

We hope that the new treatment will speed up your child's recovery from SAM. However, because these treatments are new, we do not know at this stage whether they will work and we cannot therefore guarantee any benefits for your child. Your child will continue to receive the best possible care for malnutrition as well as any new treatments we give.

We hope that our learning from this trial will benefit children with SAM in the future.

Are there any risks to participating in this study?

This is a research project, and these treatments have never before been used in children in Zimbabwe with severe acute malnutrition. We have already explained the possible risks of these treatments, and that we have designed the doses used to minimise these risks. We have considered this carefully and done our best, but the possibility remains that some

For peer review only - http://bmjopen.bmj.com/site/about/guidelines.xhtml

children might experience adverse reactions to these treatments. We can promise you that we will watch out carefully for such reactions and that we will provide the best possible care in the event that a reaction does occur.

What if I don't want my child to take part in the study or I want to withdraw later?
Participation in this study is voluntary. It is completely up to you whether or not your child participates, or if you join the trial but decide to withdraw later. If you decide for your child not to participate, it will not affect the treatment your child receives now or in the future. Whatever your decision, it will not affect your relationship with the staff caring for you.

Insurance and what to do if you have questions or concerns
In the unlikely event that your child is harmed as a result of this trial, Queen Mary University of London has agreed that you will be compensated, provided that, on the balance of probabilities, an injury was caused as a direct result of the intervention or procedures your child received during the course of the study. These special compensation arrangements apply where an injury is caused to your child that would not have occurred if s/he were not in the study. These arrangements do not affect your right to pursue a claim through legal action.

It is up to you to decide whether or not to take part. If you do decide to take part you will be given this information sheet to keep and be asked to sign a consent record form.

If you have any questions or concerns about the study or how it is conducted, you can contact Dr Bwakura-Dangarembizi, the Principal Investigator, on 0772 601 735.

For questions about your child's rights as a research subject, contact:

Medical Research Council of Zimbabwe
Cnr Mazowe /Josiah Tongogara Avenue
Harare
04 791792/791193
Mobile 0772 433 166

A COPY OF THE INFORMATION SHEET AND CONSENT RECORD WILL BE GIVEN TO YOU FOR SAFE KEEPING, AND A COPY WILL BE RETAINED BY THE STUDY TEAM

For peer review only - http://bmjopen.bmj.com/site/about/guidelines.xhtml

Please complete this form after you have read or listened to the Information Sheet and/or listened to an explanation about the research.

Therapeutic approaches to malnutrition enteropathy (TAME)
MRCZ reference: tbc

1. I have read or listened to the Participant Information Sheet for this study (version 0.7, dated 28th March, 2018).
2. The research trial has been explained to me. I understand what the research involves and what is required of me.
3. I understand that my child will have a blood test for HIV.
4. I understand that samples obtained from my child may be sent abroad for research purposes only, and that after use the samples will either be returned to Zambia for storage or destroyed.
5. I understand that there is no obligation for me to allow my child to participate in the research study, and that I do not have to give a reason for not participating.
6. I understand that if I decide at any time during the research that I no longer wish my child to participate, I can notify the researchers involved and be withdrawn from it immediately, without giving a reason.
7. I consent to the processing of our personal information for the purposes of this research study. I understand that such information will be treated as strictly confidential at all times and that any identifiable information about me or my child will be available only to members of the study team.

Participant's statement:
I ____ have read or listened to statements 1-7 above and I agree to allow my child ______________________________ to take part in the study.

Signed: Date:

Witnessed: Date:

Investigator's Statement:
I ____ confirm that I have carefully explained the nature, demands and any foreseeable risks of the proposed research to the family.

Signed: Date:

For peer review only - http://bmjopen.bmj.com/site/about/guidelines.xhtml

SPIRIT
STANDARD PROTOCOL ITEMS: RECOMMENDATIONS FOR INTERVENTIONAL TRIALS

SPIRIT 2013 Checklist: Recommended items to address in a clinical trial protocol and related documents*

Section/item	Item No	Description
**Administrative information**		
Title	1	Descriptive title identifying the study design, population, interventions, and, if applicable, trial acronym TITLE PAGE
Trial registration	2a	Trial identifier and registry name. If not yet registered, name of intended registry PAGE 2
	2b	All items from the World Health Organization Trial Registration Data Set LINK ON PAGE 2 AND THROUGHOUT DOCUMENT
Protocol version	3	Date and version identifier PAGE 2
Funding	4	Sources and types of financial, material, and other support P15
Roles and responsibilities	5a	Names, affiliations, and roles of protocol contributors P15
	5b	Name and contact information for the trial sponsor p15
	5c	Role of study sponsor and funders, if any, in study design; collection, management, analysis, and interpretation of data; writing of the report; and the decision to submit the report for publication, including whether they will have ultimate authority over any of these activities P15
	5d	Composition, roles, and responsibilities of the coordinating centre, steering committee, endpoint adjudication committee, data management team, and other individuals or groups overseeing the trial, if applicable (see Item 21a for data monitoring committee) P11
**Introduction**		
Background and rationale	6a	Description of research question and justification for undertaking the trial, including summary of relevant studies (published and unpublished) examining benefits and harms for each intervention PAGES 4-5
	6b	Explanation for choice of comparators P7
Objectives	7	Specific objectives or hypotheses P5

For peer review only - http://bmjopen.bmj.com/site/about/guidelines.xhtml

Trial design	8	Description of trial design including type of trial (eg, parallel group, crossover, factorial, single group), allocation ratio, and framework (eg, superiority, equivalence, noninferiority, exploratory) P5-6

Methods: Participants, interventions, and outcomes

Study setting	9	Description of study settings (eg, community clinic, academic hospital) and list of countries where data will be collected. Reference to where list of study sites can be obtained P6
Eligibility criteria	10	Inclusion and exclusion criteria for participants. If applicable, eligibility criteria for study centres and individuals who will perform the interventions (eg, surgeons, psychotherapists) P6-7
Interventions	11a	Interventions for each group with sufficient detail to allow replication, including how and when they will be administered P7-8
	11b	Criteria for discontinuing or modifying allocated interventions for a given trial participant (eg, drug dose change in response to harms, participant request, or improving/worsening disease) P11
	11c	Strategies to improve adherence to intervention protocols, and any procedures for monitoring adherence (eg, drug tablet return, laboratory tests) N/A, AS INPATIENT STUDY
	11d	Relevant concomitant care and interventions that are permitted or prohibited during the trial N/A
Outcomes	12	Primary, secondary, and other outcomes, including the specific measurement variable (eg, systolic blood pressure), analysis metric (eg, change from baseline, final value, time to event), method of aggregation (eg, median, proportion), and time point for each outcome. Explanation of the clinical relevance of chosen efficacy and harm outcomes is strongly recommended P9-11
Participant timeline	13	Time schedule of enrolment, interventions (including any run-ins and washouts), assessments, and visits for participants. A schematic diagram is highly recommended (see Figure) FIGURE 1
Sample size	14	Estimated number of participants needed to achieve study objectives and how it was determined, including clinical and statistical assumptions supporting any sample size calculations P10
Recruitment	15	Strategies for achieving adequate participant enrolment to reach target sample size NOT DESCRIBED

Methods: Assignment of interventions (for controlled trials)

Allocation:

Sequence generation	16a	Method of generating the allocation sequence (eg, computer-generated random numbers), and list of any factors for stratification. To reduce predictability of a random sequence, details of any planned restriction (eg, blocking) should be provided in a separate document that is unavailable to those who enrol participants or assign interventions P8
Allocation concealment mechanism	16b	Mechanism of implementing the allocation sequence (eg, central telephone; sequentially numbered, opaque, sealed envelopes), describing any steps to conceal the sequence until interventions are assigned P8
Implementation	16c	Who will generate the allocation sequence, who will enrol participants, and who will assign participants to interventions P8
Blinding (masking)	17a	Who will be blinded after assignment to interventions (eg, trial participants, care providers, outcome assessors, data analysts), and how P9
	17b	If blinded, circumstances under which unblinding is permissible, and procedure for revealing a participant’s allocated intervention during the trial P9

Methods: Data collection, management, and analysis

Data collection methods	18a	Plans for assessment and collection of outcome, baseline, and other trial data, including any related processes to promote data quality (eg, duplicate measurements, training of assessors) and a description of study instruments (eg, questionnaires, laboratory tests) along with their reliability and validity, if known. Reference to where data collection forms can be found, if not in the protocol P11
	18b	Plans to promote participant retention and complete follow-up, including list of any outcome data to be collected for participants who discontinue or deviate from intervention protocols N/A AS INPATIENT TRIAL
Data management	19	Plans for data entry, coding, security, and storage, including any related processes to promote data quality (eg, double data entry; range checks for data values). Reference to where details of data management procedures can be found, if not in the protocol DESCRIBED IN DETAIL IN PROTOCOL BUT NOT IN THIS MANUSCRIPT
Statistical methods	20a	Statistical methods for analysing primary and secondary outcomes. Reference to where other details of the statistical analysis plan can be found, if not in the protocol P10
	20b	Methods for any additional analyses (eg, subgroup and adjusted analyses) P10

	20c	Definition of analysis population relating to protocol non-adherence (eg, as randomised analysis), and any statistical methods to handle missing data (eg, multiple imputation) P10
**Methods: Monitoring**		
Data monitoring	21a	Composition of data monitoring committee (DMC); summary of its role and reporting structure; statement of whether it is independent from the sponsor and competing interests; and reference to where further details about its charter can be found, if not in the protocol. Alternatively, an explanation of why a DMC is not needed P11, BUT FULLER DECRIPTION GIVEN IN THE PROTOCOL AND DMC CHARTER
	21b	Description of any interim analyses and stopping guidelines, including who will have access to these interim results and make the final decision to terminate the trial p10
Harms	22	Plans for collecting, assessing, reporting, and managing solicited and spontaneously reported adverse events and other unintended effects of trial interventions or trial conduct p11
Auditing	23	Frequency and procedures for auditing trial conduct, if any, and whether the process will be independent from investigators and the sponsor DESCRIBED IN PROTOCOL BUT NOT IN THE MANUSCRIPT
**Ethics and dissemination**		
Research ethics approval	24	Plans for seeking research ethics committee/institutional review board (REC/IRB) approval P12-13
Protocol amendments	25	Plans for communicating important protocol modifications (eg, changes to eligibility criteria, outcomes, analyses) to relevant parties (eg, investigators, REC/IRBs, trial participants, trial registries, journals, regulators) P12
Consent or assent	26a	Who will obtain informed consent or assent from potential trial participants or authorised surrogates, and how (see Item 32) P11-12
	26b	Additional consent provisions for collection and use of participant data and biological specimens in ancillary studies, if applicable N/A
Confidentiality	27	How personal information about potential and enrolled participants will be collected, shared, and maintained in order to protect confidentiality before, during, and after the trial DESCRIBED IN THE PROTOCOL BUT NOT IN THE MANUSCRIPT
Declaration of interests	28	Financial and other competing interests for principal investigators for the overall trial and each study site P15

For peer review only

Access to data	29	Statement of who will have access to the final trial dataset, and disclosure of contractual agreements that limit such access for investigators THE TMC WILL HAVE ACCESS; NO SUCH CONTRACTUAL ARRANGEMENTS APPLY
Ancillary and post-trial care	30	Provisions, if any, for ancillary and post-trial care, and for compensation to those who suffer harm from trial participation DESCRIBED IN THE PROTOCOL BUT NOT IN THE MANUSCRIPT
Dissemination policy	31a	Plans for investigators and sponsor to communicate trial results to participants, healthcare professionals, the public, and other relevant groups (eg, via publication, reporting in results databases, or other data sharing arrangements), including any publication restrictions P12
	31b	Authorship eligibility guidelines and any intended use of professional writers N/A
	31c	Plans, if any, for granting public access to the full protocol, participant-level dataset, and statistical code P12
**Appendices**		
Informed consent materials	32	Model consent form and other related documentation given to participants and authorised surrogates INCLUDED IN SUBMISSION
Biological specimens	33	Plans for collection, laboratory evaluation, and storage of biological specimens for genetic or molecular analysis in the current trial and for future use in ancillary studies, if applicable N/A

*It is strongly recommended that this checklist be read in conjunction with the SPIRIT 2013 Explanation & Elaboration for important clarification on the items. Amendments to the protocol should be tracked and dated. The SPIRIT checklist is copyrighted by the SPIRIT Group under the Creative Commons "Attribution-NonCommercial-NoDerivs 3.0 Unported" license.

For peer review only

BMJ Open

The TAME trial: a multi-arm phase 2 randomised trial of four novel interventions for malnutrition enteropathy in Zambia and Zimbabwe; a study protocol

Journal:	*BMJ Open*
Manuscript ID	bmjopen-2018-027548.R2
Article Type:	Protocol
Date Submitted by the Author:	21-Mar-2019
Complete List of Authors:	Kelly, Paul; Barts and The London School of Medicine<br>Bell, Lauren; London School of Hygiene and Tropical Medicine<br>Amadi, Beatrice; 2Tropical Gastroenterology and Nutrition Group, University of Zambia, Lusaka, Zambia<br>Bwakura, Mutsa<br>VanBuskirk, Kelley<br>Chandwe, Kanta; Tropical Gastroenterology and Nutrition Group, University of Zambia, Lusaka, Zambia<br>Chipunza, Miyoba; Tropical Gastroenterology & Nutrition Group<br>Ngosa, Deophine; Tropical Gastroenterology and Nutrition Group, University of Zambia, Lusaka, Zambia<br>Chulu, Nivea; 2Tropical Gastroenterology and Nutrition Group, University of Zambia, Lusaka, Zambia<br>Hill, Susan; Great Ormond Street Hospital For Children NHS Trust,<br>Murch, Simon ; Warwick Medical School, University of Warwick, Division of Metabolic and Vascular Health<br>Playford, Raymond; University of Plymouth School of Biological Sciences<br>Prendergast, Andrew ; Queen Mary University of London,
**Primary Subject Heading**:	Nutrition and metabolism
Secondary Subject Heading:	Gastroenterology and hepatology, Global health, Paediatrics
Keywords:	Paediatric gastroenterology < GASTROENTEROLOGY, Tropical medicine < INFECTIOUS DISEASES, NUTRITION & DIETETICS, Paediatric gastroenterology < PAEDIATRICS, Clinical trials < THERAPEUTICS

SCHOLARONE™
Manuscripts

The TAME trial: a multi-arm phase 2 randomised trial of four novel interventions for malnutrition enteropathy in Zambia and Zimbabwe; a study protocol

Paul Kelly[1,2,3], Lauren Bell[3], Beatrice Amadi[2], Mutsa Bwakura-Dangarembizi[4,5], Kelley VanBuskirk[2], Kanta Chandwe[2], Miyoba Chipunza[2], Deophine Ngosa[2], Nivea Chulu[2], Susan Hill[6], Simon Murch[7], Raymond Playford[1], Andrew J Prendergast[1,5]

[1]Barts & The London School of Medicine, Queen Mary University of London, London, UK; [2]University of Zambia School of Medicine, Lusaka, Zambia; [3]London School of Hygiene and Tropical Medicine, London, UK; [4]Department of Paediatrics and Child Health, College of Health Sciences, University of Zimbabwe, Harare, Zimbabwe; [5]Zvitambo Institute for Maternal and Child Health Research, Harare, Zimbabwe; [6]Great Ormond Street Hospital, London, UK; [7]University Hospital Coventry & Warwickshire, Coventry, UK.

Corresponding author:

Professor Paul Kelly MD FRCP

Blizard Institute

Barts & The London School of Medicine

Turner Street

London E1 2AD

UK

tel: 020 7882 2643

email: m.p.kelly@qmul.ac.uk

For peer review only - http://bmjopen.bmj.com/site/about/guidelines.xhtml

ABSTRACT

Introduction

Severe acute malnutrition (SAM) in children in many countries still carries unacceptably high mortality, especially when complicated by secondary infection or metabolic derangements. New therapies are urgently needed and we have identified mucosal healing in the intestine as a potential target for novel treatment approaches.

Methods and analysis

The TAME trial (therapeutic approaches for malnutrition enteropathy) will evaluate four novel treatments in an efficient multi-arm single-blind phase II design. In three hospitals in Zambia and Zimbabwe, 225 children with SAM will be randomised to one of these treatments or to standard care, once their inpatient treatment has reached the point of transition from stabilisation to increased nutritional intake. The four interventions are budesonide, bovine colostrum or N-acetyl glucosamine given orally or via nasogastric tube, or teduglutide given by subcutaneous injection. The primary endpoint will be a composite score of faecal inflammatory markers, and a range of secondary endpoints include clinical and laboratory endpoints. Treatments will be given daily for 14 days, and evaluation of the major endpoints will be at 14-18 days, with a final clinical evaluation at 28 days. In a subset of children in Zambia, endoscopic biopsies will be used to evaluate the effect of interventions in detail.

Ethics and dissemination

The study has been approved by the University of Zambia Biomedical Research Ethics Committee (006-09-17, dated 9th July, 2018), and the Joint Research Ethics Committee of the University of Zimbabwe (24th July, 2019). Caregivers will provide written informed consent for each participant. Findings will be disseminated through peer-reviewed journals, conference presentations and to caregivers at face-to-face meetings.

Registration

The trial is registered at https://clinicaltrials.gov/ct2/show/NCT03716115. Version 2.0 (7th October 2019) used in the preparation of this manuscript.

ARTICLE SUMMARY; strengths and limitations of the trial

Strengths

- Efficient multi-arm trial design
- Evaluation of potential efficacy of four novel interventions for a life-threatening disorder of great importance for global health
- Measurement of a broad range of biomarkers and measures of mucosal pathology

For peer review only - http://bmjopen.bmj.com/site/about/guidelines.xhtml

Potential limitations

- Only 14 days of treatment will be tested; although longer treatment may be effective, the greatest need is for treatments which confer benefit in the period of highest mortality
- There is no consensus on optimal markers of mucosal healing, hence the need for a wide range of endpoints.

For peer review only

For peer review only - http://bmjopen.bmj.com/site/about/guidelines.xhtml

BACKGROUND AND RATIONALE

Nutritional disorders are glaring examples of health inequalities between high- and low-income countries, and within low-income countries. Malnutrition underlies almost half of all child deaths globally and therefore contributes enormously to the unacceptably high under-5 mortality rates in these regions[1]. Chronic undernutrition, which usually manifests as stunting (poor linear growth), affects 40% and 27% of children in Zambia and Zimbabwe, respectively[2], and is associated with increased mortality[3], reduced neurodevelopmental potential and decreased long-term economic productivity[4]. Acute malnutrition usually manifests as wasting (loss of tissue), with or without oedema, and is the most conspicuous of all nutritional disorders. Severe acute malnutrition (SAM) carries the highest mortality[5], particularly if associated with medical complications. HIV has changed the epidemiology, pathogenesis and clinical presentation of SAM, and children with both conditions have a particularly high mortality[6].

Over the last two decades, three key developments in the approach to treatment have improved the outcomes of SAM: standardised management protocols, ready to use therapeutic food (RUTF), and community management of acute malnutrition (CMAM), which enables identification and management of children without medical complications of SAM[7,8]. However, severely malnourished children with medical complications still require hospitalisation,[9] and often fail to respond to treatment[10]. Inpatient mortality among children with complicated SAM remains up to 38%[5,11,12,13]. Even after discharge, children have a poor prognosis, with up to 42% mortality documented over the subsequent year[6]. In our experience, it is a subgroup of children with SAM and acute or persistent diarrhoea who pose the most difficult management challenges, although the vast majority of children with SAM and/or stunting have a degree of enteropathy[11,14,15].

Recent studies[16,17] have taught us a great deal about the contribution of enteric infections to dysfunction of the small intestine in malnourished children. A high pathogen burden causes damage to the mucosa which exacerbates nutritional impairment and leads to further susceptibility to infection and impaired epithelial regeneration, in a cyclical process first described in the 1970s[18,19]. This mucosal damage in SAM we refer to as *malnutrition enteropathy*, which is characterised by multiple epithelial breaches, microbial translocation from the gut lumen to the systemic circulation, and systemic inflammation[20]. Epithelial breaches are present in histological sections, seen in haematoxylin/eosin-stained sections and using immunofluorescence for claudin 4 and E-cadherin[20,21]. In parallel studies in adults, we demonstrated that these lesions occur *in vivo* using confocal laser endomicroscopy[21]. We have also identified a consistent pattern of blunted

For peer review only - http://bmjopen.bmj.com/site/about/guidelines.xhtml

epithelial repair, with reduced glucagon-like peptide 2 (GLP2) in serum, reduced trefoil factor 3 in duodenal aspirates[21], and a strong transcriptomic signature of impaired mucosal defence. We also observed two further immunological abnormalities: low-level false positive coeliac-like autoantibodies[20], and upregulation of SMAD7 somewhat similar to the pattern seen in Crohn's disease[22]. We have previously reported that markers of microbial translocation and systemic inflammation were dramatically increased in children with SAM in Zambia compared to healthy controls[20]. Together, these abnormalities indicate there is substantial structural and functional damage to the small intestine[12], and that this primary gut pathology is associated with systemic sequelae. Importantly, biomarkers of these processes have been associated with mortality among children hospitalized with complicated SAM[12].

Current treatment guidelines for SAM are not well supported by an evidence base, and there is a dearth of clinical trial data; in particular, there are no specific interventions to target enteropathy in SAM[5]. In a systematic review[23], only three trials were found which inform management of SAM and persistent diarrhoea, and no trials dealing with the HIV-infected child. We therefore believe that novel therapeutic approaches are urgently needed, and that a series of small phase 2 trials will help guide development of a new generation of treatments. These trials should focus on repairing damage to the small intestinal mucosa, as we now have substantial evidence that this plays a central role in the genesis of systemic inflammation, bacterial translocation and sepsis, though it is possible that it could be a consequence of inflammation. We propose evaluation of four new interventions: bovine colostrum, N-acetylglucosamine, teduglutide and budesonide.

OBJECTIVES

To determine if four new interventions (bovine colostrum, N-acetylglucosamine, teduglutide and budesonide) can ameliorate malnutrition enteropathy in children with severe acute malnutrition in Zambia or Zimbabwe. It is hoped that one or more of these interventions may be sufficiently promising to take through into phase 3 trials.

METHODS AND ANALYSIS

Trial design

There is emerging awareness that multi-arm Phase 2 clinical trials offer a more efficient approach to identification of new therapies than parallel conventional trial designs, in which one agent is evaluated against a control. Our recent work on the pathophysiology of malnutrition enteropathy suggests that mucosal healing may be central to reducing mortality, but we do not yet know the

For peer review only

efficacy of several plausible therapeutic approaches. In order to evaluate these four new potential therapies efficiently and quickly, they will be compared side-by-side using biomarkers of pathophysiology as endpoints. This design has the following advantages:

i. The four novel therapies will each be evaluated against standard care;

ii. Allocation to all five treatment groups will run in parallel, so that all children will be randomised to any of the five groups at any point in time to avoid biases due to seasonal variation;

iii. The trial endpoints will evaluate a range of biomarkers that capture different domains of malnutrition enteropathy, to allow a comprehensive non-invasive assessment of mucosal healing;

iv. A subgroup of children (those recruited in Lusaka) will have small intestinal endoscopy in a unit with a track record of safety in children.

v. Endoscopic biopsy will provide evaluation of healing and ensure that the selected therapy does not induce unwanted immune or other effects;

vi. The overall trial design provides an efficient way of identifying one or more candidates for a future Phase 3 trial.

Study setting and participant recruitment

The study will be conducted in hospitals in Lusaka, Zambia, and Harare, Zimbabwe, which have experience of several previous studies of severe acute malnutrition[11,20,24]. Children hospitalised with SAM will be eligible for inclusion if they are aged 6-59 months, of either sex, are an inpatient in one of the children's wards of the participating hospitals, have initiated transition (from F75 feed to F100 or RUTF), and clinically stable. SAM is defined using WHO definitions: weight-for-length/height Z-score of less than -3, or mid upper arm circumference of less than 11.5cm, and/or bilateral pedal oedema. During the Stabilization Phase of treatment of SAM, readiness for transition is determined by the ability to finish F75 feed by mouth and reduction in oedema (if patient was admitted with oedematous SAM). Such a child is deemed to be "clinically stable" and in general is alert, not requiring oxygen, not hypothermic, hypoglycaemic and is not in shock or dehydrated.

Children will be excluded if they have weight less than 5kg, a neurological disability or oro-facial abnormality which would explain or partly explain poor feeding, haemoglobin concentration 6 g/dl or less, contraindication to any of the trial treatments (e.g. allergy to cow's milk protein), or any

For peer review only - http://bmjopen.bmj.com/site/about/guidelines.xhtml

underlying condition, other than HIV, which in the opinion of the investigator would put the subject at undue risk of failing study completion or would interfere with analysis of study results. If the caregiver is unwilling to remain in hospital for the duration of the study treatment (14 days) they will not be recruited.

Interventions: investigational therapies

Colostrum is the first liquid secreted by the lactating breast, and breast-fed children take it for approximately the first three days of life. It is similar to breast milk, but with higher protein content. Bovine colostrum is available as a high-protein bovine colostrum powder (Neovite) for use as a health-promoting nutritional supplement. Colostrum contains nutrients, immunoglobulins and growth factors, including epidermal growth factor (EGF) and insulin-like growth factor 1 (IGF-1); it has been shown to reduce intestinal permeability in adults[25]. Bovine colostrum is potentially allergenic in theory, though there is no evidence that cow's milk products are harmful in children with SAM and the standard therapeutic feeds (F75 and F100 recommended by WHO) contain milk proteins.

N-acetyl glucosamine (GlcNAc) is a natural amino-sugar present on every cell surface. All breast-fed children consume GlcNAc in human milk throughout lactation[26]. Impaired glycosylation of glycosaminoglycans has been noted for many years in oedematous malnutrition, with reduced concentrations of glycosaminoglycans in blood, urine, kidney, brain and small intestine. Specific consequences of reduced heparan sulphate expression include gut epithelial leakiness with hypoalbuminaemia[27]. GlcNAc administration has been demonstrated to restore the intestinal epithelial charged barrier in Crohn's disease[28]. As with all sugars, GlcNAc may theoretically induce osmotic diarrhoea if not absorbed in the small intestine. Although this has not been observed clinically in animals or in older children, we will use a dose escalation schedule to minimise the chance of this happening.

Teduglutide is a long-acting form of GLP2 which has proven efficacy in intestinal failure, improving absorption and reducing the need for parenteral support[29]. GLP2 is a hormone secreted by L cells in the terminal ileum, which drives epithelial repair and mediates intestinal adaptation by increased cellular proliferation and villus hypertrophy. Teduglutide will be given by subcutaneous injection (0.05mg/kg/day) daily for 14 days. In a recently published description of tolerability of 12 weeks of teduglutide in children in the UK and the USA[30], vomiting was attributed to teduglutide in

For peer review only - http://bmjopen.bmj.com/site/about/guidelines.xhtml

10% of recipients and pyrexia in 14%. No children had treatment withdrawal because of adverse events. Three adverse events were considered of special interest (intestinal obstruction, fluid overload and biliary derangements) but none were observed.

Budesonide is a corticosteroid which reduces inflammation in the gut but is then rapidly broken down in the liver which minimises systemic effects. Budesonide is standard therapy for Crohn's disease, and can be used for refractory coeliac disease. Since malnutrition enteropathy is characterized by intestinal inflammation, with infiltration of activated T cells[31], an anti-inflammatory approach is rational. A prior trial of mesalazine in Kenya confirmed that an immuno-modulatory approach with mesalazine was safe in the setting of SAM[32], but targeting the small, rather than large, intestine with a more potent agent may be more effective. Budesonide may cause immunosuppression and other corticosteroid effects (oedema, hypertension, glucose intolerance, osteoporosis), but usually only after longer-term administration. It is the corticosteroid of choice for intestinal disorders because it causes fewer adverse events than prednisolone because of low systemic absorption and first-pass clearance by the liver. The dose will be tapered (1mg orally three times daily for 7 days, then 1mg twice daily for 4 days, then 0.5mg twice daily for 3 days) to mitigate any possible effects of adrenal suppression.

Trial procedures

The trial schema is shown in Figure 1. Children hospitalised with SAM will be enrolled from any of three hospitals (Lusaka Children's Hospital, Lusaka, Zambia; Parirenyatwa Hospital, Harare, Zimbabwe; Harare Central Hospital, Harare, Zimbabwe) once they have completed the stabilization phase of nutritional rehabilitation and are at the point of transition from F75 therapeutic milk (low-calorie feed) to ready-to-use therapeutic food (RUTF) or F100 milk (feeds richer in calories). Children will be randomised in an allocation ratio of (1:1:1:1:1) to either Colostrum, GlcNAc, Teduglutide, Budesonide or Standard Care and followed for 14 days in hospital (Table 1). All children will receive Standard Care according to WHO guidelines[33], and treatment with trial medications will commence after transition.

Randomisation codes will be prepared in advance by the trial statistician, and treatment allocation will be revealed after enrolment by opening sealed envelopes held by the trial coordinators in each of the three study sites. Randomisation codes will be generated in permuted blocks and stratified by study site. Varying block sizes will be used; block size and distribution will not be

For peer review only - http://bmjopen.bmj.com/site/about/guidelines.xhtml

disclosed to the study clinical teams. There will be no attempt at blinding as the interventions are readily distinguishable (e.g. teduglutide is given as subcutaneous injection, so to mask all interventions would require administration of several placebo formulations). However, the primary endpoints are biomarkers so laboratory staff and data analysts will be blinded to randomised group.

Outcomes and endpoints

The primary endpoint for this trial will be measured on day 15 (allowable window 15 to 19 days) after initiating treatment by analysis of faecal biomarkers. Gut inflammation will be measured as a composite score of faecal myeloperoxidase, neopterin and alpha-1 antitrypsin, as described below. Secondary endpoints (Table 2) will be measured by daily clinical review (for clinical endpoints) during the intervention period and by collection of biological specimens on day 15 (allowable window 15 to 19 days) after initiating treatment. As endoscopic biopsies will be collected from trial participants in Lusaka, we anticipate that at least twenty biopsy scores will be available for each trial arm. Few trials have used histological endpoints in SAM, so this component of the trial is comparatively novel and therefore exploratory.

Sample size and data analysis

Kosek et al[34] examined established markers of intestinal inflammation which can be used to obtain population-based measures of intestinal injury and altered intestinal function. They developed an environmental enteropathy (EE) activity score which is a composite score of three stool markers for mucosal inflammation. The composite score is derived from measurements of faecal myeloperoxidase (MPO), neopterin (NEO) and alpha1-antitrypsin (AAT). These are stable compounds, relatively resistant to proteolysis and therefore suitable as biomarkers.

The weighted EE score is given in Equation 1:

Equation 1

EE score = 2 x (AAT category) + 2 x (MPO category) + 1 (1 x NEO category)

We will use the principle of a weighted enteropathy score as our primary outcome. We will not trichotomise the variables as Kosek et al[34] did, but keep the score as a continuous composite variable. This is because we are interested in gauging the potential magnitude of the effect these treatments have, and applying a cut-off value to a continuous composite score could mask or

For peer review only - http://bmjopen.bmj.com/site/about/guidelines.xhtml

discard information[35,36]. The modified Enteropathy score is given in Equation 2, with modification of weighting of units to bring measurements into a comparable arithmetical range:

Equation 2

EE biomarker score = 2x(AAT in mg/g) + 0.2x(MPO in µg/ml) + 1x(neopterin in µmol/l).

We will have a control arm, so can understand the effect of the treatment against a reference standard. The analysis will be per-protocol; no interim analysis is planned.

There are no available data from children with SAM to draw upon for an understanding of the baseline variance of the EE biomarker score. The primary outcome response from each patient is the EE biomarker score at 15 days (window 15-19 days) after treatment initiation, adjusted for baseline values. We have assumed that the EE biomarker score is normally distributed with a common standard deviation. To determine the sample size, we drew upon two methods to establish an effect size. We assumed the difference would be larger than the likely inherent imprecision in the measurements of each outcome, and we have defined the magnitude of the effect on a standardised scale. To detect a medium/large effect of Cohen's *d* effect size of 0.6, with 90% power and 90% confidence, and a conservative correlation between baseline and follow-up estimate of 0.5, we will need a sample size of 36 per group across 5 groups to analyse with the ANCOVA method (180 in total to be treated). We expect there to be approximately 10% loss to follow up due to deaths, and 10% loss to follow up due to other reasons, such as drug intolerance, withdrawal and missing specimens. Adjusting our sample size of 180 for 20% loss to follow-up, we therefore aim to randomise 225 patients in total (45 in each group).

We will use a mixed effect ANCOVA model to compare the environmental enteropathy activity score and secondary endpoints in each group against the control group, adjusting for several core covariates: sex (male/female); oedema (yes/no); HIV status (yes/no); diarrhoea (yes/no); breastfeeding (yes/no); baseline WLZ scores (continuous); baseline biomarker/histology scores (continuous) and trial site. Treatment effects will be deemed statistically significant if the p-value is less than or equal to 0.10 when compared to the control arm, for all 4 treatments. This less stringent cut-off has been chosen to reduce the likelihood of rejecting a potentially valuable treatment which might show benefit in a phase 3 trial. All trial committee members (see below) will contribute to a discussion at the end of the trial as to which, if any, of these treatment effects are most clinically significant.

For peer review only - http://bmjopen.bmj.com/site/about/guidelines.xhtml

We will not undertake any adjustments of the false positive (type I) error rate, as the aim of this trial is to inform the treatment development process for this population, which would lead to a Phase III trial if a degree of efficacy is observed. The general consensus is that adjustment for the type I error rate is not required in exploratory multi-arm multi-stage trials in Phase II within the treatment development framework[37].

If patients are discharged from hospital sooner than day 14, we will measure the primary endpoints as close as the discharge date as possible, and carry this observation forward as the primary outcome. Children who cannot submit a stool sample will be excluded from the primary analysis as the primary endpoint requires this.

Safety reporting

All adverse events will be reviewed for causality, expectedness and severity. Serious adverse events will be reported urgently to the Trial Management Group for evaluation, and all serious adverse reactions will be regarded as unexpected (i.e. SUSARs). There are two adverse events of special interest for teduglutide: fluid overload and intestinal obstruction, as these have been reported in adults on long-term teduglutide. Complicated SAM has an inpatient mortality rate of around 15%, so we anticipate up to 45 deaths in the trial; however, all deaths, regardless of causality, will be reported to the local ethics committees in Zimbabwe and Zambia according to local reporting policies. The trial will be supervised by three committees: the Trial Management Group (TMG), the Data Monitoring and Ethics Committee (DMEC, which will meet every 6 months) and the Trial Steering Committee (TSC). The TMG and TSC will review the pattern of any SAEs and SUSARs in real time, and are empowered to introduce urgent special measures if the profile of adverse events suggests this is required. The TSC and DMEC are composed of statisticians and paediatricians, and independent of the TMG.

Patient and public involvement

There was no patient or public involvement in the design of this trial.

ETHICS AND DISSEMINATION

Consent

Ethical approval for the trial has already been obtained (as of 18th March, 2019) from the

For peer review only - http://bmjopen.bmj.com/site/about/guidelines.xhtml

University of Zambia Biomedical Research Ethics Committee (reference 006-09-17) and the Joint research Ethics Committee of the University of Zimbabwe and the College of Health Sciences (JREC/66/19). Approvals have also been obtained from the Zambian Medicines Regulatory Authority (CT/082/18) and the Medicines Control Authority of Zimbabwe (CT/176/2019). Trial nursing and medical staff will identify eligible children whose primary caregivers will then be approached to begin the process of discussing the trial and the written, translated, information sheet (see Supplementary material) in the most appropriate language. The consent process will include honest discussions of risks and benefits of the interventions, the concept of randomisation, and the purpose and intended use of samples collected, which is an important issue in this trial as the primary endpoints are all laboratory biomarkers. The consent process will continue throughout the trial, but all parents/guardians will sign to record that they have given informed consent to their child's participation, before any study procedures commence. Parents of children who cannot read will have the information sheet read out to them in the presence of family members before consent is accepted and recorded using a thumbprint. We have pioneered the use of participant visits to laboratories to facilitate valid consent[38]. This opportunity will be afforded to these parents and guardians. At least 24 hours will be available for consideration of consent; in practice, 2-3 days is the norm in Zambia and Zimbabwe because mothers/carers usually need to discuss enrolment with other family members. The window of time available for recruitment at and after transition allows for this consultation process.

Dissemination of results

Dissemination of results to participants' families will take place through caregiver results meetings. We will disseminate results within the academic institutions in which the trial will be conducted and to national health research authorities. Trial results will be published following CONSORT guidelines, and participant-level dataset made available through a data repository. Improvements in care will ultimately occur through change in evidence-based practice. By collaborating with colleagues on design of a future interventional trial based on our results, and by sharing specimens with other research groups, we intend to push forward the research agenda for children with SAM[39]. We will also use this trial as an opportunity to increase awareness in the UK of the complexity of malnutrition. The Centre of the Cell (www.centreofthecell.org) at Queen Mary University of London is a unique, state-of-the-art science education centre with extensive experience of translating complex medical research into innovative science communication tools. We are currently working with CoC to develop a game to explain the causes of undernutrition, including the role of the gut in nutrition, with endoscopy footage, animations and interactive

For peer review only - http://bmjopen.bmj.com/site/about/guidelines.xhtml

gameplay . The game will be reformatted for tablets for use in Harare and Lusaka. The production of multiple formats of the tool will facilitate adults and children in the UK and Africa to access this resource and learn about current research on SAM.

Time frame and study status

The trial has been granted ethical approval by all required regulatory authorities in Zambia and Zimbabwe. The trial should be completed in 2021.

DISCUSSION

In the belief that reducing mortality from SAM will require novel therapeutic approaches, and recognising abundant evidence of impaired intestinal function, we have identified mucosal healing in the small intestine as a target for intervention. The TAME trial will employ a multi-arm design to identify one or more of four interventions for taking forward into phase 3 trials. The strengths of this trial include an efficient design, which will evaluate four novel interventions against standard care, and very extensive assessment of mucosal healing and biomarkers to provide the greatest chance of observing potential benefit. Limitations of the trial include the short duration of treatment, ongoing uncertainty about the optimal selection of biomarkers for accurate representation of intestinal absorptive and barrier function (calprotectin is not included in the primary endpoint, for example), and the complexity of data interpretation in this group of children with serious morbidity and high predicted mortality. To ease with identification of adverse events we considered it prudent to delay recruitment until the child has stabilised. However this also means that an important, possibly critical, window for therapy during the first few days of admission may be missed. Hopefully this can be addressed in future studies.

We have considered carefully the issue of duration of treatment. Inpatient treatment of SAM lasts usually between 1-2 weeks and it is in this narrow time window that most, though not all[6], of the mortality occurs. If novel therapies for reduction of mortality in SAM are to be useful at scale, they must confer benefit quickly, during the 1-2 week period immediately after admission. While it would be of great interest to evaluate more extended periods of therapy, the most urgent need is for new treatments in this short period of maximum morbidity and mortality. In future trials of any promising interventions, the timing of introduction of novel therapies would be an explicit research question, as they may also be of value earlier in treatment, or continued for a longer duration, including after discharge. However, for this phase II trial, we will only enrol children once

For peer review only - http://bmjopen.bmj.com/site/about/guidelines.xhtml
For peer review only

they are clinically stable, at the transition phase, and provide treatment over 14 days during the typical period of hospitalisation.

Two of these agents (bovine colostrum and N-acetyl glucosamine) are nutraceuticals, food-derived and generally regarded as safe. While this is very reassuring about the low probability of adverse events, it is hard to quantify that probability with any accuracy. Regulatory agencies are more familiar with IMPs which have well-described adverse event profiles based on careful phase 1 trials, so paradoxically the level of scrutiny of any potential adverse events in the TAME trial will be higher than if full SmPCs were publically available, despite the expected low risk of significant AEs.

Teduglutide has not previously been used for treating enteropathy in malnutrition, but there is growing evidence of its benefit in intestinal failure[29]. Children with SAM have evidence of a hyperplastic enteropathy which is likely to lead to impaired absorption of nutrients. Teduglutide is a GLP2 analogue which can enhance intestinal adaptation and reduce requirements for parenteral nutrition in patient receiving it. It may overcome some of the impaired absorption in severe enteropathies, which may enhance nutrient uptake in SAM. However there are possible adverse effects, including increased salt and water retention which (although not reported in previous studies in children[30]) will be an adverse event of special interest in TAME. Intestinal obstruction has also been reported in adults as a consequence of mucosal hypertrophy; it too will be an adverse event of special interest.

In summary, we propose a phase 2 trial designed to target malnutrition enteropathy, which we believe is central to the pathogenesis of SAM, prevents recovery, and is not currently addressed by current interventions. Identification of promising novel treatment approaches would provide a strong rationale for a larger efficacy trial to reduce the unacceptably high morbidity, mortality and relapse among children with complicated SAM.

For peer review only - http://bmjopen.bmj.com/site/about/guidelines.xhtml

Authors' contributions

PK, AJP, BA and MB-D originated the trial idea and obtained funding. KvB and LB provided statistical expertise. MC, DN, NC, and MC collected preliminary data for sample size calculations. KC, RP, SM and SH provided specific input into design of specific elements of the trial, particularly the principal interventions. All authors contributed to and approved the final manuscript.

Funding statement

The TAME trial is funded by the Medical Research Council (MR/P024033/1). AJP and MB-D are funded by the Wellcome Trust (grants 093768/Z/15/Z and 107634/Z/15/Z, respectively). These funding agencies have had no role in the preparation of this manuscript.

Competing interests

The authors declare that they have no competing interests

Word count

4,231

Trial Sponsor

Queen Mary University of London

For peer review only

For peer review only - http://bmjopen.bmj.com/site/about/guidelines.xhtml

References

1 Black RE, Victora CG, Walker SP, Bhutta ZA, Christian P, de Onis M, Ezzati M, Grantham-McGregor S, Katz J, Martorell R, Uauy R. Maternal and child undernutrition and overweight in low-income and middle-income countries. Lancet. 2013; 382: 427-51.

2 Zambia Demographic and Health Survey 2013-14; Zimbabwe Demographic and Health Survey 2015. Rockville, Maryland, USA: Central Statistical Office, Ministry of Health, and ICF International.

3 Olofin I, McDonald CM, Ezzati M, Flaxman S, Black RE, Fawzi WW, Caulfield LE, Danaei G. Associations of suboptimal growth with all-cause and cause-specific mortality in children under five years: a pooled analysis of ten prospective studies. PLoS One 2013 8: e64636.

4 Victora CG, Adair L, Fall C, Hallal PC, Martorell R, Richter L, Sachdev HS. Maternal and child undernutrition: consequences for adult health and human capital. Lancet 2008; 371: 340–357.

5 Lenters LM, Wazny K, Webb P, Ahmed T, Bhutta ZA. Treatment of severe and moderate acute malnutrition in low- and middle-income settings: a systematic review, meta-analysis and Delphi process. BMC Public Health. 2013; 13 Suppl 3: S23.

6 Kerac M, Bunn J, Chagaluka G, Bahwere P, Tomkins A, Collins S, Seal A. Follow-up of post-discharge growth and mortality after treatment for severe acute malnutrition (FuSAM study): a prospective cohort study. PLoS One 2014; 9: e96030.

7 Collins S, Dent N, Binns P, Bahwere P, Sadler K, Hallam A. Management of severe acute malnutrition in children. Lancet 2006; 368: 1992-2000.

8 Amadi B, Imasiku M, Sakala M, Banda R, Kelly P. Integration of HIV care into community management of acute childhood malnutrition permits good outcomes: retrospective analysis of three years of a programme in Lusaka. PLoS One 2016; 11: e0149218.

9 WHO Guideline: Updates on the management of severe acute malnutrition in infants and children. Geneva: World Health Organization; 2013.

10 Isanaka S, Langendorf C, Berthé F, Gnegne S, Li N, Ousmane N, Harouna S, Hassane H, Schaefer M, Adehossi E, Grais RF. Routine Amoxicillin for Uncomplicated Severe Acute Malnutrition in Children. N Engl J Med 2016; 374: 444-53.

11 Amadi BC, Kelly P, Mwiya M, Mulwazi E, Sianongo S, Changwe F, Thomson M, Hachungula J, Watuka A, Walker-Smith JA, Chintu C. Intestinal and systemic infection, HIV and mortality in Zambian children with persistent diarrhoea and malnutrition. J Ped Gastroenterol Nutr 2001; 32: 550-554.

12 Attia S, Versloot CJ, Voskuijl W, van Vliet SJ, Di Giovanni V, Zhang L, Richardson S, Bourdon C, Netea MG, Berkley JA, van Rheenen PF, Bandsma RH. Mortality in children with complicated severe acute malnutrition is related to intestinal and systemic inflammation: an observational

For peer review only - http://bmjopen.bmj.com/site/about/guidelines.xhtml

cohort study. Am J Clin Nutr 2016; 104:1441–9.

13 Bartels RH, Bourdon C, Potani I, Mhango B, van den Brink DA, Mponda JS, Muller Kobold AC, Bandsma RH, Boele van Hensbroek M, Voskuijl WP. Pancreatic Enzyme Replacement Therapy in Children with Severe Acute Malnutrition: A Randomized Controlled Trial. J Pediatr. 2017; 190: 85-92.e2.

14 Prendergast A, Kelly P. Interactions between intestinal pathogens, enteropathy and malnutrition in developing countries. Curr Op Infect Dis 2015; 29; 229-236.

15 Trehan I, Kelly P, Shaikh N, Manary MJ. New insights into Environmental Enteric Dysfunction. Arch Dis Child 2016; 101:741-4.

16 Kotloff KL, et al. Burden and aetiology of diarrhoeal disease in infants and young children in developing countries (the Global Enteric Multicenter Study, GEMS): a prospective, case-control study. Lancet 2013; 382: 209–22.

17 Platts-Mills JA, et al. Pathogen-specific burdens of community diarrhoea in developing countries: a multisite birth cohort study (MAL-ED). Lancet Glob Health 2015; 3: e564–75.

18 Scrimshaw, NS, et al. Monogr Ser World Health Organ, 1968. 57: 3-329.

19 Mata LJ. The Children of Santa Maria Cauqué: A Prospective Field Study of Health and Growth (International nutrition policy series) MIT Press: 1978.

20 Amadi B, Besa E, Zyambo K, Kaonga P, Louis-Auguste J, Chandwe K, Tarr PI, Denno DM, Nataro JP, Faubion W, Sailer A, Yeruva S, Brantner T, Murray JA, Prendergast AJ, Turner JR, Kelly P. Impaired barrier function and autoantibody generation in malnutrition enteropathy in Zambia. EBiomedicine 2017; 22: 191-199.

21 Kelly P, Besa E, Zyambo K, Louis-Auguste J, Lees J, Banda T, Soko R, Banda R, Amadi B, Watson A. Endomicroscopic and transcriptomic analysis of impaired barrier function and malabsorption in environmental enteropathy. PLoS Negl Trop Dis 2016; 10: e0004600.

22 Syed S, Dinallo V, Iqbal N, Di Iorio L, Di Fusco D, Guleria S, Amadi BC, Sadiq K, Moskaluk C, Ali A, Kelly P, Monteleone G. High SMAD7 and p-SMAD2,3 expression is associated with environmental enteropathy in children. *PLoS Negl Trop Dis* 2018; **12**: e0006224. doi: 10.1371/journal.pntd.0006224.

23 Picot J, et al. The effectiveness of interventions to treat severe acute malnutrition in young children: a systematic review. Health Tech Assessment 2012; 16: 19.

24 Bwakura-Dangarembizi M, Amadi B, Bourke CD, Robertson RC, Mwapenya B, Chandwe K, Kapoma C, Chifunda K, Majo F, Ngosa D, Chakara P, Chulu N, Masimba F, Mapurisa I, Besa E, Mutasa K, Mwakamui S, Runodamoto T, Humphrey JH, Ntozini R, Wells J, Manges AR, Swann J, Walker AS, Nathoo KJ, Kelly P, Prendergast AJ. Health Outcomes, Pathogenesis and Epidemiology of Severe

For peer review only - http://bmjopen.bmj.com/site/about/guidelines.xhtml

Acute Malnutrition (HOPE-SAM): rationale and methods of a longitudinal observational study. *BMJ Open*; in press.

25 Marchbank T, Davison G, Oakes JR, Ghatei MA, Patterson M, Moyer MP, Playford RJ. The nutriceutical bovine colostrum truncates the increase in gut permeability caused by heavy exercise in athletes. Am J Physiol Gastrointest Liver Physiol 2011; 300: G477-84.

26 Bode L. The functional biology of human milk oligosaccharides. Early Hum Dev 2015; 91: 619-22.

27 Bode L, Salvestrini C, Park PW, Li JP, Esko JD, Yamaguchi Y, Murch S, Freeze HH. Heparan sulfate and syndecan-1 are essential in maintaining murine and human intestinal epithelial barrier function. J Clin Invest 2008; 118: 229-238.

28 Salvatore S, Heuschkel R, Tomlin S, Davies SE, Edwards S, Walker-Smith JA, French I, Murch SH. A pilot study of N-acetyl glucosamine, a nutritional substrate for glycosaminoglycan synthesis, in paediatric chronic inflammatory bowel disease. Aliment Pharmacol Ther 2000; 14: 1567-1579.

29 Jeppesen PB, Pertkiewicz M, Messing B, Iyer K, Seidner DL, O'Keefe SJ, Forbes A, Heinze H, Joelsson B. Teduglutide reduces need for parenteral support among patients with short bowel syndrome with intestinal failure. Gastroenterol 2012; 143: 1473-1481.

30 Carter BA, Cohran VC, Cole CR, Corkins MR, Dimmitt RA, Duggan C, Hill S, Horslen S, Lim JD, Mercer DF, Merritt RJ, Nichol PF, Sigurdsson L, Teitelbaum DH, Thompson J, Vanderpool C, Vaughan JF, Li B, Youssef NN, Venick RS, Kocoshis SA. Outcomes from a 12-week, open-label, multicenter clinical trial of teduglutide in pediatric short bowel syndrome. J Pediatr. 2017; 181: 102-111.e5. doi: 10.1016/j.jpeds.2016.10.027.

31 Veitch AM, Kelly P, Zulu I, Segal I, Farthing MJG. Tropical enteropathy: a T cell mediated crypt hyperplastic enteropathy. Eur J Gastroenterol Hepatol 2001;13:1175-81.

32 Jones KD, Hünten-Kirsch B, Laving AM, Munyi CW, Ngari M, Mikusa J, Mulongo MM, Odera D, Nassir HS, Timbwa M, Owino M, Fegan G, Murch SH, Sullivan PB, Warner JO, Berkley JA. Mesalazine in the initial management of severely acutely malnourished children with environmental enteric dysfunction: a pilot randomized controlled trial. BMC Med. 2014;12:133.

33 WHO Guideline: Updates on the management of severe acute malnutrition in infants and children. Geneva: World Health Organization; 2013.

34 Kosek M, Haque R, Lima A, Babji S, Shrestha S, Qureshi S, Amidou S, Mduma E, Lee G, Yori PP, Guerrant RL, Bhutta Z, Mason C, Kang G, Kabir M, Amour C, Bessong P, Turab A, Seidman J, Olortegui MP, Quetz J, Lang D, Gratz J, Miller M, Gottlieb M. Fecal markers of intestinal inflammation and permeability associated with the subsequent acquisition of linear growth deficits in infants. Am J Trop Med Hyg 2013; 88: 390-6. doi: 10.4269/ajtmh.2012.12-0549. E

For peer review only - http://bmjopen.bmj.com/site/about/guidelines.xhtml

35 Altman DG, Royston P. The cost of dichotomising continuous variables. BMJ 2006; 332: 1080.

36 Wason JMS, Seaman SR. Using continuous data on tumour measurements to improve inference in phase II cancer studies. Statistics in Medicine 2013; 32: 4639-4650.

37 Wason JM, Stecher L, Mander AP. Correcting for multiple-testing in multi-arm trials: is it necessary and is it done? Trials 2014; 15: 364.

38 Zulu J, Mwanza-Lisulo M, Besa E, Kaonga P, Chisenga CC, Chomba M, Simuyandi M, Banda R, Kelly P. Improving validity of informed consent for biomedical research in Zambia using a laboratory exposure intervention. PLOS One 2014; 9: e108305. doi: 10.1371/journal.pone.0108305.

39 Heikens GT, Bunn J, Amadi B, Manary M, Chhagan M, Berkley JA, Rollins N, Kelly P, Adamczick C, Maitland K, Tomkins A. Case management of HIV-infected, severely malnourished children in sub-Saharan Africa: challenges in the area if highest prevalence. Lancet 2008; 371: 1305-1307.

For peer review only

For peer review only - http://bmjopen.bmj.com/site/about/guidelines.xhtml

Table 1 Trial interventions in the five randomisation groups

**Group**	**C**	**N**	**T**	**B**	**S**
IMP	Colostrum	N-acetyl glucosamine	Teduglutide	Budesonide	Standard care
Presentation	Powder in capsules	Powder in capsules	Ampoules	Liquid	-
Number of patients	45	45	45	45	45
Dose:					
days 1-7	1.5g tds	300mg tds	0.05 mg/kg daily	1mg tds	-
days 8-11	1.5g tds	600mg tds	0.05 mg/kg daily	1mg bd	
days 12-14	1.5g tds	600mg tds	0.05 mg/kg daily	0.5mg bd	-

All IMPs are to be given for 14 days

For peer review only

For peer review only - http://bmjopen.bmj.com/site/about/guidelines.xhtml

Table 2 Endpoints to be measured in the TAME trial

**Primary endpoint**	Units	How measured
Faecal α1-antitrypsin, myeloperoxidase and neopterin concentrations	mg/g, µg/ml, µmol/l	ELISA
**Secondary endpoints**		
Lactulose:rhamnose ratio (Zambia only)		HPLC
Plasma intestinal fatty acid binding protein (FABP) concentration	ng/ml	ELISA
Plasma lipopolysaccharide (LPS)	EU/ml	Limulus amoebacyte lysate (LAL)
Plasma LPS binding protein (LBP)	ng/ml	ELISA
Plasma soluble CD14 and CD163	µg/ml	ELISA
Plasma C-reactive protein (CRP)	mg/l	ELISA
Plasma albumin	g/l	ELISA
Mortality by day 14 and day 28		Clinical assessment
Change in anthropometric measurements (weight, mid upper arm circumference, weight-for-height, MUAC) between baseline and day 14, and baseline and day 28		Weight and length measurements
Resolution of oedema between baseline and day 14		Clinical assessment
Adverse events between baseline and day 14		Clinical assessment
Serious adverse events between baseline and day 14		Clinical assessment
Days with diarrhoea (three or more loose or watery stools in 24 hours) between baseline and day 14		Clinical assessment
Days with fever (two or more recordings of core temperature of 37.8°C or higher in any 24 h period) between baseline and day 14		Clinical assessment

For peer review only - http://bmjopen.bmj.com/site/about/guidelines.xhtml

Hormones: GLP-2, IGF-1 and IGFBP3	ng/ml	ELISA
Morphometric measures on biopsy specimens collected in Zambia only: villus height, crypt depth, villus width, epithelial surface perimeter	µm	Microscopy
Mucosal inflammatory scores	Ordinal scale	Microscopy

MUAC, mid upper arm circumference; GLP-2, glucagon-like peptide 2; IGF, insulin-like growth factor; IGFBP, IGF binding protein.

For peer review only

Figure legends

Figure 1 Flowchart of recruitment into the TAME trial

For peer review only

For peer review only - http://bmjopen.bmj.com/site/about/guidelines.xhtml

TAME flow chart

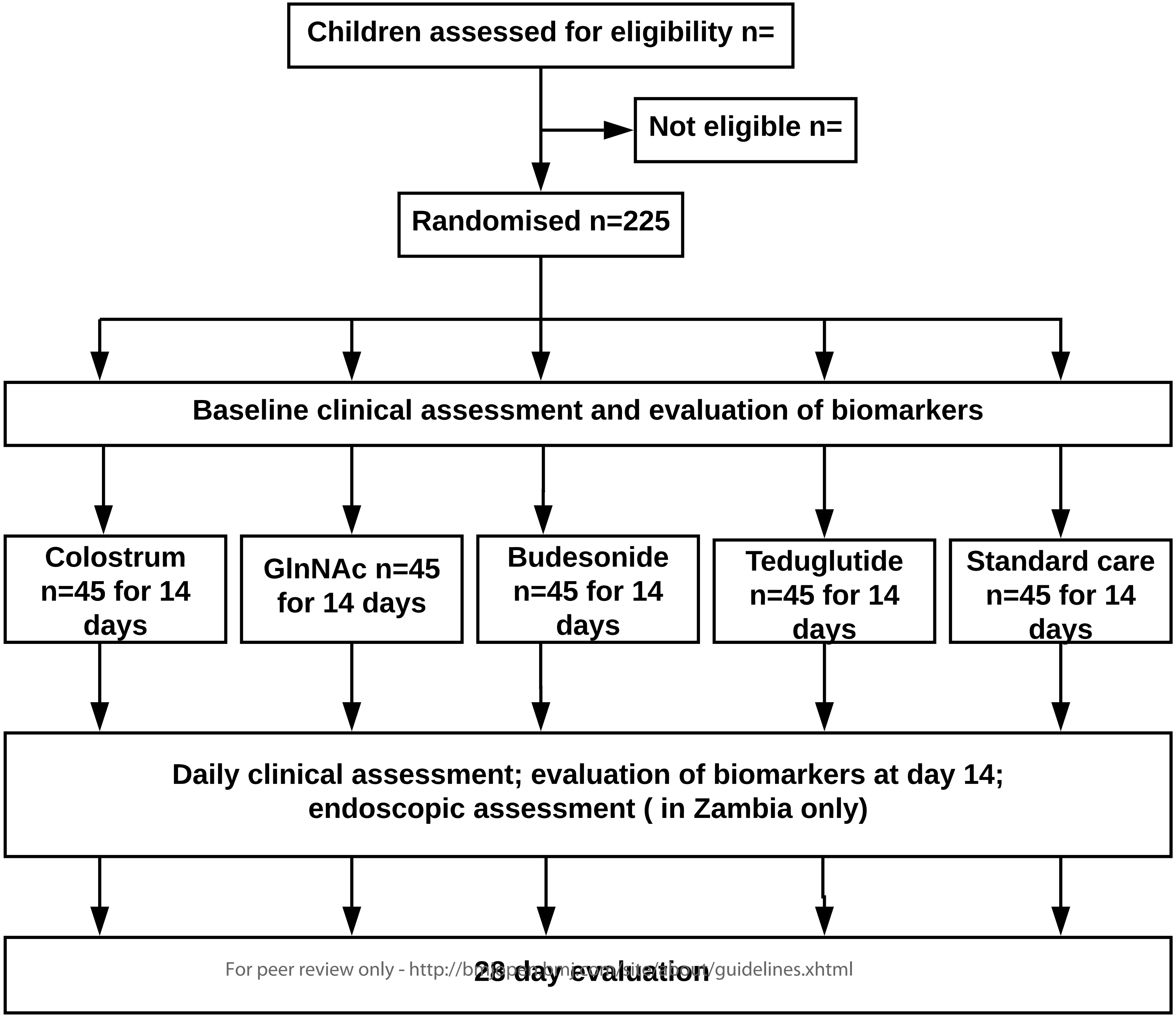

For peer review only - http://bmjopen.bmj.com/site/about/guidelines.xhtml

Therapeutic approaches to malnutrition enteropathy (TAME): phase II trials of four novel interventions in children in Zambia and Zimbabwe

Zambia

Principal Investigator for Zambia: Dr Beatrice Amadi
Chief Investigator: Professor Paul Kelly

Participant Information Sheet

We are researchers from the University of Zambia School of Medicine and Lusaka Children's Hospital. We would like to invite you and your child to be part of this research project. You should only agree that your child could take part if you want to; it is entirely up to you. If you choose your child not to take part, there will be no disadvantages. Choosing not to take part will not affect your child's treatment in any way, now or in the future. Please read or listen to the following information carefully before you decide that your child can take part. If you decide to take part, you will be asked to sign a consent form to record your agreement. You are free to withdraw at any time during your child's participation without giving a reason.

<u>Why we are doing this trial</u>

The purpose of this trial is to develop extra treatments to help in the full recovery of children with Severe Acute Malnutrition (SAM). We know that children with SAM are at increased risk of dying. We know that malnutrition causes damage to the gut (intestine), which can allow it to become 'leaky'. We believe that if this does not heal properly, children do not recover from malnutrition. We want to try giving new medicines to heal the damaged intestine.

<u>Why has my child been chosen?</u>

We have identified that your child has severe acute malnutrition with complications. We want to include your child in a trial of some new treatments.

<u>What the study involves</u>

There are four new treatments which we want to compare against the treatments we currently use. Current treatments closely follow the guidelines of the World Health Organisation, including special feeds and fluids, and antibiotics. We do not know if these new treatments are better than the treatments we already have so we need to compare them in a randomised clinical trial. This means that the choice of treatments is made randomly, by chance (like the flip of a coin). For one out of five children, the treatment will remain exactly the same care as we give now. For four out of five children, we will give all the current treatments plus a new drug. A set of envelopes has been prepared with numbers on. If you agree that your child is to be included in the trial, a study number will be given. Once that number has been given we will open the envelope with that number and see which new treatment should be given, or if your child should not be given a new treatment. It is very important that you understand these two principles of a randomised trial:

1. We genuinely do not know which treatment is best, or if they are better or worse than what we do already.

For peer review only - http://bmjopen.bmj.com/site/about/guidelines.xhtml

2. Children will be allocated a treatment by chance.

Once the envelope has been opened, we will follow the treatment course indicated on the contents of the envelope. We will not choose the treatment for your child.

The treatments

There are four possible treatments, but your child will only receive one of these at most. We do not know which one until the envelope is opened. All of these medicines need to be given for 2 weeks in hospital, not at home:

1. **Colostrum**. When mothers give birth, the first type of milk that comes from the breast is colostrum, which is a yellowy colour. This is full of protective substances for the baby and we think it also may help to heal the gut. We have obtained colostrum from cows as one of the new treatments. It is as safe as the cow's milk we all drink in our tea, but there is the small possibility that it may be too rich for babies with severe acute malnutrition and may cause or worsen diarrhoea. We will watch out for such effects. It is given by mouth or down the gastric tube.
2. **N-acetyl glucosamine (GlucNac)**. This is a sort of starch which we hope will build up the protective mucus which lines our intestines. There is the small possibility that it may be too rich for children with severe acute malnutrition and may cause or worsen diarrhoea. We will watch out for such effects. It is given by mouth or down the gastric tube.
3. **Teduglutide (TDG)**. This is a modified version of a natural hormone the body produces, which helps the gut to repair itself after injury. It has been used in children with very severe gut failure, and has helped them to absorb more nutrients from the milk they get. A recent trial in the USA and the UK shows that it is safe, but it can cause vomiting and fever in about one in ten children who receive it for 3 months. We think it is unlikely that this will occur when the treatment is given for 14 days, but we will watch out for that. It is given as an injection under the skin, once every day.
4. **Budesonide**. This is a steroid, used to help heal the gut in children with other sorts of gut disease. Steroids can have side effects, including fluid accumulation and high levels of sugar in the blood and weakened resistance to infection, but usually only when they are given for longer periods. Budesonide is designed to treat gut inflammation without being absorbed, but we will watch out for any effects. It is given by mouth or down the gastric tube.

The investigations your child will have

When your child enters the study, he/she will have a full medical assessment and examination. All these tests are completely safe. We will also ask for an additional blood sample, a stool sample, and a saliva sample, then we do a simple test which involves drinking a small amount of a sugary liquid and collecting two urine samples. To reduce the pain of taking blood, we can use a jelly on your child's skin which very much reduces the discomfort. We will then take another set of blood samples after one week; and blood, urine, saliva and stool samples after two weeks. The total volume of blood we will collect for research is limited to a safe quantity (2ml/kg body weight in total during the trial). We will carry out HIV testing as part of the tests on blood samples; the results will be completely confidential even if it is negative. All children will be monitored daily by our team of doctors and nurses for the full two weeks, and then we will ask you to come back

For peer review only - http://bmjopen.bmj.com/site/about/guidelines.xhtml

on the 28th day so that we can give your child a final growth check and ask a few questions.

In addition, between days 14 and 18 we will carry out an endoscopy. This means examining the inside of the stomach and intestine with a small flexible tube, through which we can collect tiny pieces of the lining of the intestine, each about the size of the head of a match. Although this sounds rather worrying, in fact this is quite safe because we have good facilities and a great deal of experience. The endoscopy requires giving anaesthetic injections; this is done by an anaesthetist who monitors the children very carefully.

If you would like, we would be very happy to show you the endoscopy unit and equipment at UTH so you have a better idea of what is involved. We can also show you the laboratories so you can see what we do with the samples we take, and how we analyse them.

How many children will be in the study?

The study is being conducted in Zambia and Zimbabwe. There will be 225 children in total in the study.

What we do with your child's samples

We only use the samples you provide for research. Any samples that we do not need for research will be destroyed. We have no need to use the samples you provide for any other purpose, including giving them to people who are not involved in the research. It is also against the law to do so.

We do nearly all of the research analysis in the laboratories at UTH. Some of the tests will need to be done by our collaborators in the UK or USA, so some of your child's samples may be sent there. These collaborating laboratories are not allowed to do anything with the samples that isn't to do with research, including selling them or giving them to people who aren't involved in the research. We are very strict on this and Prof Kelly can give you absolute reassurance that the samples are only used for this research. Once the research is finished they will be destroyed.

We do many different measurements on the samples you provide, and if you are interested we can demonstrate and explain some of these to you, including how the samples are stored.

What we do with the information you provide

During the research we collect information about you and your child. We store the information on computers and remove your name and your child's name and any other ways of being identified, so that there is no way of telling who the information refers to. Any and all information you give us is confidential. This means that we are not allowed to tell anyone about you or your child, even if they are friends or family.

How this study might benefit you

For peer review only - http://bmjopen.bmj.com/site/about/guidelines.xhtml
For peer review only

We hope that the new treatment will speed up your child's recovery from SAM. However, because these treatments are new, we do not know at this stage whether they will work and we cannot therefore guarantee any benefits for your child. Your child will continue to receive the best possible care for malnutrition as well as any new treatments we give.

We hope that our learning from this trial will benefit children with SAM in the future.

Are there any risks to participating in this study?

This is a research project, and these treatments have never before been used in children in Zambia with severe acute malnutrition. We have already explained the possible risks of these treatments, and that we have designed the doses used to minimise these risks. We have considered this carefully and done our best, but the possibility remains that some children might experience adverse reactions to these treatments. We can promise you that we will watch out carefully for such reactions and that we will provide the best possible care in the event that a reaction does occur.

What if I don't want my child to take part in the study or I want to withdraw later?

Participation in this study is voluntary. It is completely up to you whether or not your child participates, or if you join the trial but decide to withdraw later. If you decide for your child not to participate, it will not affect the treatment your child receives now or in the future. Whatever your decision, it will not affect your relationship with the staff caring for you.

Insurance and what to do if you have questions or concerns

In the unlikely event that your child is harmed as a result of this trial, Queen Mary University of London has agreed that you will be compensated, provided that, on the balance of probabilities, an injury was caused as a direct result of the intervention or procedures your child received during the course of the study. These special compensation arrangements apply where an injury is caused to your child that would not have occurred if s/he were not in the study. These arrangements do not affect your right to pursue a claim through legal action.

It is up to you to decide whether or not to take part. If you do decide to take part you will be given this information sheet to keep and be asked to sign a consent record form.

If you have any questions or concerns about the study or how it is conducted, you can contact Professor Kelly's and Dr Amadi's team in the hospital at any point

Contact details are:

Professor Paul Kelly
Endoscopy Unit, Clinic 5,
University Teaching Hospital
Lusaka
Tel 0211 252269/0955 295493
or Dr Beatrice Amadi
Ward A07
Lusaka Children's Hospital

For peer review only - http://bmjopen.bmj.com/site/about/guidelines.xhtml

Lusaka
tel: 0966 752739
If you are not happy to do this, or are not happy with the advice you get from Professor Kelly's and Dr Amadi's team, you can contact:

The University of Zambia Biomedical Research Ethics Committee,
University of Zambia School of Medicine,
Ridgeway Campus,
PO Box 50110,
Lusaka.
tel: 0211 250753 and speak to Lorna

A COPY OF THE INFORMATION SHEET AND CONSENT RECORD WILL BE GIVEN TO YOU FOR SAFE KEEPING, AND A COPY WILL BE RETAINED BY THE STUDY TEAM

For peer review only

For peer review only - http://bmjopen.bmj.com/site/about/guidelines.xhtml

Please complete this form after you have read or listened to the Information Sheet and/or listened to an explanation about the research.

Therapeutic approaches to malnutrition enteropathy (TAME)
UNZABREC reference: tbc

1. I have read or listened to the Participant Information Sheet for this study (version 0.7, dated 28th March, 2018).
2. The research trial has been explained to me. I understand what the research involves and what is required of me.
3. I understand that my child will have a blood test for HIV.
4. I understand that samples obtained from my child may be sent abroad for research purposes only, and that after use the samples will either be returned to Zambia for storage or destroyed.
5. I understand that there is no obligation for me to allow my child to participate in the research study, and that I do not have to give a reason for not participating.
6. I understand that if I decide at any time during the research that I no longer wish my child to participate, I can notify the researchers involved and be withdrawn from it immediately, without giving a reason.
7. I consent to the processing of our personal information for the purposes of this research study. I understand that such information will be treated as strictly confidential at all times and that any identifiable information about me or my child will be available only to members of the study team.

Participant's statement:
I ____ have read or listened to statements 1-7 above and I agree to allow my child ______________________________ to take part in the study.

Signed: Date:

Witnessed: Date:

Investigator's Statement:
I ____ confirm that I have carefully explained the nature, demands and any foreseeable risks of the proposed research to the family.

Signed: Date:

For peer review only - http://bmjopen.bmj.com/site/about/guidelines.xhtml

Therapeutic approaches to malnutrition enteropathy (TAME): phase II trials of four novel interventions in children in Zambia and Zimbabwe

ZIMBABWE

Principal Investigator for Zimbabwe: Dr Mutsa Bwakura-Dangarembizi
Chief Investigator: Professor Paul Kelly

Participant Information Sheet
We are researchers from the University of Zimbabwe College of Health Sciences, Harare Central Hospital and Parirenyatwa Hospital. We would like to invite you and your child to be part of this research project. You should only agree that your child could take part if you want to; it is entirely up to you. If you choose your child not to take part, there will be no disadvantages. Choosing not to take part will not affect your child's treatment in any way, now or in the future. Please read or listen to the following information carefully before you decide that your child can take part. If you decide to take part, you will be asked to sign a consent form to record your agreement. You are free to withdraw at any time during your child's participation without giving a reason.

<u>Why we are doing this trial</u>
The purpose of this trial is to develop extra treatments to help in the full recovery of children with Severe Acute Malnutrition (SAM). We know that children with SAM are at increased risk of dying. We know that malnutrition causes damage to the gut (intestine), which can allow it to become 'leaky'. We believe that if this does not heal properly, children do not recover from malnutrition. We want to try giving new medicines to heal the damaged intestine.

<u>Why has my child been chosen?</u>
We have identified that your child has severe acute malnutrition with complications. We want to include your child in a trial of some new treatments.

<u>What the study involves</u>
There are four new treatments which we want to compare against the treatments we currently use. Current treatments closely follow the guidelines of the World Health Organisation, including special feeds and fluids, and antibiotics. We do not know if these new treatments are better than the treatments we already have so we need to compare them in a randomised clinical trial. This means that the choice of treatments is made randomly, by chance (like the flip of a coin). For one out of five children, the treatment will remain exactly the same care as we give now. For four out of five children, we will give all the current treatments plus a new drug. A set of envelopes has been prepared with numbers on. If you agree that your child is to be included in the trial, a study number will be given. Once that number has been given we will open the envelope with that number and see which new treatment should be given, or if your child should not be given a new treatment. It is very important that you understand these two principles of a randomised trial:

1. We genuinely do not know which treatment is best, or if they are better or worse than what we do already.

For peer review only - http://bmjopen.bmj.com/site/about/guidelines.xhtml

2. Children will be allocated a treatment by chance.

Once the envelope has been opened, we will follow the treatment course indicated on the contents of the envelope. We will not choose the treatment for your child.

<u>The treatments</u>

There are four possible treatments, but your child will only receive one of these at most. We do not know which one until the envelope is opened. All of these medicines need to be given for 2 weeks in hospital, not at home:

1. **Colostrum**. When mothers give birth, the first type of milk that comes from the breast is colostrum, which is a yellowy colour. This is full of protective substances for the baby and we think it also may help to heal the gut. We have obtained colostrum from cows as one of the new treatments. It is as safe as the cow's milk we all drink in our tea, but there is the small possibility that it may be too rich for babies with severe acute malnutrition and may cause or worsen diarrhoea. We will watch out for such effects. It is given by mouth or down the gastric tube.
2. **N-acetyl glucosamine (GlucNac)**. This is a sort of starch which we hope will build up the protective mucus which lines our intestines. There is the small possibility that it may be too rich for children with severe acute malnutrition and may cause or worsen diarrhoea. We will watch out for such effects. It is given by mouth or down the gastric tube.
3. **Teduglutide (TDG)**. This is a modified version of a natural hormone the body produces, which helps the gut to repair itself after injury. It has been used in children with very severe gut failure, and has helped them to absorb more nutrients from the milk they get. A recent trial in the USA and the UK shows that it is safe, but it can cause vomiting and fever in about one in ten children who receive it for 3 months. We think it is unlikely that this will occur when the treatment is given for 14 days, but we will watch out for that. It is given as an injection under the skin, once every day.
4. **Budesonide**. This is a steroid, used to help heal the gut in children with other sorts of gut disease. Steroids can have side effects, including fluid accumulation and high levels of sugar in the blood and weakened resistance to infection, but usually only when they are given for longer periods. Budesonide is designed to treat gut inflammation without being absorbed, but we will watch out for any effects. It is given by mouth or down the gastric tube.

<u>The investigations your child will have</u>

When your child enters the study, he/she will have a full medical assessment and examination. All these tests are completely safe. We will also ask for an additional blood sample, a stool sample, and a saliva sample, then we do a simple test which involves drinking a small amount of a sugary liquid and collecting two urine samples. To reduce the pain of taking blood, we can use a jelly on your child's skin which very much reduces the discomfort. We will then take another set of blood samples after one week; and blood, urine, saliva and stool samples after two weeks. The total volume of blood we will collect for research is limited to a safe quantity (2ml/kg body weight in total during the trial). We will carry out HIV testing as part of the tests on blood samples; the results will be completely confidential even if it is negative. All children will be monitored daily by our team of doctors and nurses for the full two weeks, and then we will ask you to come back

For peer review only - http://bmjopen.bmj.com/site/about/guidelines.xhtml

on the 28th day so that we can give your child a final growth check and ask a few questions.
If you would like, we would be very happy to show you the laboratories so you can see what we do with the samples we take, and how we analyse them.

How many children will be in the study?

The study is being conducted in Zambia and Zimbabwe. There will be 225 children in total in the study.

What we do with your child's samples

We only use the samples you provide for research. Any samples that we do not need for research will be destroyed. We have no need to use the samples you provide for any other purpose, including giving them to people who are not involved in the research. It is also against the law to do so.

We do nearly all of the research analysis in the laboratories in Harare. Some of the tests will need to be done by our collaborators in the UK or USA, so some of your child's samples may be sent there. These collaborating laboratories are not allowed to do anything with the samples that isn't to do with research, including selling them or giving them to people who aren't involved in the research. We are very strict on this and Prof Kelly can give you absolute reassurance that the samples are only used for this research. Once the research is finished they will be destroyed.

We do many different measurements on the samples you provide, and if you are interested we can demonstrate and explain some of these to you, including how the samples are stored.

What we do with the information you provide

During the research we collect information about you and your child. We store the information on computers and remove your name and your child's name and any other ways of being identified, so that there is no way of telling who the information refers to. Any and all information you give us is confidential. This means that we are not allowed to tell anyone about you or your child, even if they are friends or family.

How this study might benefit you

We hope that the new treatment will speed up your child's recovery from SAM. However, because these treatments are new, we do not know at this stage whether they will work and we cannot therefore guarantee any benefits for your child. Your child will continue to receive the best possible care for malnutrition as well as any new treatments we give.

We hope that our learning from this trial will benefit children with SAM in the future.

Are there any risks to participating in this study?

This is a research project, and these treatments have never before been used in children in Zimbabwe with severe acute malnutrition. We have already explained the possible risks of these treatments, and that we have designed the doses used to minimise these risks. We have considered this carefully and done our best, but the possibility remains that some

For peer review only - http://bmjopen.bmj.com/site/about/guidelines.xhtml

children might experience adverse reactions to these treatments. We can promise you that we will watch out carefully for such reactions and that we will provide the best possible care in the event that a reaction does occur.

What if I don't want my child to take part in the study or I want to withdraw later?
Participation in this study is voluntary. It is completely up to you whether or not your child participates, or if you join the trial but decide to withdraw later. If you decide for your child not to participate, it will not affect the treatment your child receives now or in the future. Whatever your decision, it will not affect your relationship with the staff caring for you.

Insurance and what to do if you have questions or concerns
In the unlikely event that your child is harmed as a result of this trial, Queen Mary University of London has agreed that you will be compensated, provided that, on the balance of probabilities, an injury was caused as a direct result of the intervention or procedures your child received during the course of the study. These special compensation arrangements apply where an injury is caused to your child that would not have occurred if s/he were not in the study. These arrangements do not affect your right to pursue a claim through legal action.

It is up to you to decide whether or not to take part. If you do decide to take part you will be given this information sheet to keep and be asked to sign a consent record form.

If you have any questions or concerns about the study or how it is conducted, you can contact Dr Bwakura-Dangarembizi, the Principal Investigator, on 0772 601 735.

For questions about your child's rights as a research subject, contact:

Medical Research Council of Zimbabwe
Cnr Mazowe /Josiah Tongogara Avenue
Harare
04 791792/791193
Mobile 0772 433 166

A COPY OF THE INFORMATION SHEET AND CONSENT RECORD WILL BE GIVEN TO YOU FOR SAFE KEEPING, AND A COPY WILL BE RETAINED BY THE STUDY TEAM

For peer review only - http://bmjopen.bmj.com/site/about/guidelines.xhtml

Please complete this form after you have read or listened to the Information Sheet and/or listened to an explanation about the research.

Therapeutic approaches to malnutrition enteropathy (TAME)
MRCZ reference: tbc

1. I have read or listened to the Participant Information Sheet for this study (version 0.7, dated 28th March, 2018).
2. The research trial has been explained to me. I understand what the research involves and what is required of me.
3. I understand that my child will have a blood test for HIV.
4. I understand that samples obtained from my child may be sent abroad for research purposes only, and that after use the samples will either be returned to Zambia for storage or destroyed.
5. I understand that there is no obligation for me to allow my child to participate in the research study, and that I do not have to give a reason for not participating.
6. I understand that if I decide at any time during the research that I no longer wish my child to participate, I can notify the researchers involved and be withdrawn from it immediately, without giving a reason.
7. I consent to the processing of our personal information for the purposes of this research study. I understand that such information will be treated as strictly confidential at all times and that any identifiable information about me or my child will be available only to members of the study team.

Participant's statement:
I ____ have read or listened to statements 1-7 above and I agree to allow my child ______________________________ to take part in the study.

Signed: Date:

Witnessed: Date:

Investigator's Statement:
I ____ confirm that I have carefully explained the nature, demands and any foreseeable risks of the proposed research to the family.

Signed: Date:

For peer review only - http://bmjopen.bmj.com/site/about/guidelines.xhtml

SPIRIT

STANDARD PROTOCOL ITEMS: RECOMMENDATIONS FOR INTERVENTIONAL TRIALS

SPIRIT 2013 Checklist: Recommended items to address in a clinical trial protocol and related documents*

Section/item	Item No	Description
**Administrative information**		
Title	1	Descriptive title identifying the study design, population, interventions, and, if applicable, trial acronym TITLE PAGE
Trial registration	2a	Trial identifier and registry name. If not yet registered, name of intended registry PAGE 2
	2b	All items from the World Health Organization Trial Registration Data Set LINK ON PAGE 2 AND THROUGHOUT DOCUMENT
Protocol version	3	Date and version identifier PAGE 2
Funding	4	Sources and types of financial, material, and other support P15
Roles and responsibilities	5a	Names, affiliations, and roles of protocol contributors P15
	5b	Name and contact information for the trial sponsor p15
	5c	Role of study sponsor and funders, if any, in study design; collection, management, analysis, and interpretation of data; writing of the report; and the decision to submit the report for publication, including whether they will have ultimate authority over any of these activities P15
	5d	Composition, roles, and responsibilities of the coordinating centre, steering committee, endpoint adjudication committee, data management team, and other individuals or groups overseeing the trial, if applicable (see Item 21a for data monitoring committee) P11
**Introduction**		
Background and rationale	6a	Description of research question and justification for undertaking the trial, including summary of relevant studies (published and unpublished) examining benefits and harms for each intervention PAGES 4-5
	6b	Explanation for choice of comparators P7
Objectives	7	Specific objectives or hypotheses P5

For peer review only

Trial design	8	Description of trial design including type of trial (eg, parallel group, crossover, factorial, single group), allocation ratio, and framework (eg, superiority, equivalence, noninferiority, exploratory) P5-6

Methods: Participants, interventions, and outcomes

Study setting	9	Description of study settings (eg, community clinic, academic hospital) and list of countries where data will be collected. Reference to where list of study sites can be obtained P6
Eligibility criteria	10	Inclusion and exclusion criteria for participants. If applicable, eligibility criteria for study centres and individuals who will perform the interventions (eg, surgeons, psychotherapists) P6-7
Interventions	11a	Interventions for each group with sufficient detail to allow replication, including how and when they will be administered P7-8
	11b	Criteria for discontinuing or modifying allocated interventions for a given trial participant (eg, drug dose change in response to harms, participant request, or improving/worsening disease) P11
	11c	Strategies to improve adherence to intervention protocols, and any procedures for monitoring adherence (eg, drug tablet return, laboratory tests) N/A, AS INPATIENT STUDY
	11d	Relevant concomitant care and interventions that are permitted or prohibited during the trial N/A
Outcomes	12	Primary, secondary, and other outcomes, including the specific measurement variable (eg, systolic blood pressure), analysis metric (eg, change from baseline, final value, time to event), method of aggregation (eg, median, proportion), and time point for each outcome. Explanation of the clinical relevance of chosen efficacy and harm outcomes is strongly recommended P9-11
Participant timeline	13	Time schedule of enrolment, interventions (including any run-ins and washouts), assessments, and visits for participants. A schematic diagram is highly recommended (see Figure) FIGURE 1
Sample size	14	Estimated number of participants needed to achieve study objectives and how it was determined, including clinical and statistical assumptions supporting any sample size calculations P10
Recruitment	15	Strategies for achieving adequate participant enrolment to reach target sample size NOT DESCRIBED

Methods: Assignment of interventions (for controlled trials)

Allocation:

Sequence generation	16a	Method of generating the allocation sequence (eg, computer-generated random numbers), and list of any factors for stratification. To reduce predictability of a random sequence, details of any planned restriction (eg, blocking) should be provided in a separate document that is unavailable to those who enrol participants or assign interventions P8
Allocation concealment mechanism	16b	Mechanism of implementing the allocation sequence (eg, central telephone; sequentially numbered, opaque, sealed envelopes), describing any steps to conceal the sequence until interventions are assigned P8
Implementation	16c	Who will generate the allocation sequence, who will enrol participants, and who will assign participants to interventions P8
Blinding (masking)	17a	Who will be blinded after assignment to interventions (eg, trial participants, care providers, outcome assessors, data analysts), and how P9
	17b	If blinded, circumstances under which unblinding is permissible, and procedure for revealing a participant's allocated intervention during the trial P9

Methods: Data collection, management, and analysis

Data collection methods	18a	Plans for assessment and collection of outcome, baseline, and other trial data, including any related processes to promote data quality (eg, duplicate measurements, training of assessors) and a description of study instruments (eg, questionnaires, laboratory tests) along with their reliability and validity, if known. Reference to where data collection forms can be found, if not in the protocol P11
	18b	Plans to promote participant retention and complete follow-up, including list of any outcome data to be collected for participants who discontinue or deviate from intervention protocols N/A AS INPATIENT TRIAL
Data management	19	Plans for data entry, coding, security, and storage, including any related processes to promote data quality (eg, double data entry; range checks for data values). Reference to where details of data management procedures can be found, if not in the protocol DESCRIBED IN DETAIL IN PROTOCOL BUT NOT IN THIS MANUSCRIPT
Statistical methods	20a	Statistical methods for analysing primary and secondary outcomes. Reference to where other details of the statistical analysis plan can be found, if not in the protocol P10
	20b	Methods for any additional analyses (eg, subgroup and adjusted analyses) P10

	20c	Definition of analysis population relating to protocol non-adherence (eg, as randomised analysis), and any statistical methods to handle missing data (eg, multiple imputation) P10
**Methods: Monitoring**		
Data monitoring	21a	Composition of data monitoring committee (DMC); summary of its role and reporting structure; statement of whether it is independent from the sponsor and competing interests; and reference to where further details about its charter can be found, if not in the protocol. Alternatively, an explanation of why a DMC is not needed P11, BUT FULLER DECRIPTION GIVEN IN THE PROTOCOL AND DMC CHARTER
	21b	Description of any interim analyses and stopping guidelines, including who will have access to these interim results and make the final decision to terminate the trial p10
Harms	22	Plans for collecting, assessing, reporting, and managing solicited and spontaneously reported adverse events and other unintended effects of trial interventions or trial conduct p11
Auditing	23	Frequency and procedures for auditing trial conduct, if any, and whether the process will be independent from investigators and the sponsor DESCRIBED IN PROTOCOL BUT NOT IN THE MANUSCRIPT
**Ethics and dissemination**		
Research ethics approval	24	Plans for seeking research ethics committee/institutional review board (REC/IRB) approval P12-13
Protocol amendments	25	Plans for communicating important protocol modifications (eg, changes to eligibility criteria, outcomes, analyses) to relevant parties (eg, investigators, REC/IRBs, trial participants, trial registries, journals, regulators) P12
Consent or assent	26a	Who will obtain informed consent or assent from potential trial participants or authorised surrogates, and how (see Item 32) P11-12
	26b	Additional consent provisions for collection and use of participant data and biological specimens in ancillary studies, if applicable N/A
Confidentiality	27	How personal information about potential and enrolled participants will be collected, shared, and maintained in order to protect confidentiality before, during, and after the trial DESCRIBED IN THE PROTOCOL BUT NOT IN THE MANUSCRIPT
Declaration of interests	28	Financial and other competing interests for principal investigators for the overall trial and each study site P15

review only

Access to data	29	Statement of who will have access to the final trial dataset, and disclosure of contractual agreements that limit such access for investigators THE TMC WILL HAVE ACCESS; NO SUCH CONTRACTUAL ARRANGEMENTS APPLY
Ancillary and post-trial care	30	Provisions, if any, for ancillary and post-trial care, and for compensation to those who suffer harm from trial participation DESCRIBED IN THE PROTOCOL BUT NOT IN THE MANUSCRIPT
Dissemination policy	31a	Plans for investigators and sponsor to communicate trial results to participants, healthcare professionals, the public, and other relevant groups (eg, via publication, reporting in results databases, or other data sharing arrangements), including any publication restrictions P12
	31b	Authorship eligibility guidelines and any intended use of professional writers N/A
	31c	Plans, if any, for granting public access to the full protocol, participant-level dataset, and statistical code P12
**Appendices**		
Informed consent materials	32	Model consent form and other related documentation given to participants and authorised surrogates INCLUDED IN SUBMISSION
Biological specimens	33	Plans for collection, laboratory evaluation, and storage of biological specimens for genetic or molecular analysis in the current trial and for future use in ancillary studies, if applicable N/A

*It is strongly recommended that this checklist be read in conjunction with the SPIRIT 2013 Explanation & Elaboration for important clarification on the items. Amendments to the protocol should be tracked and dated. The SPIRIT checklist is copyrighted by the SPIRIT Group under the Creative Commons "Attribution-NonCommercial-NoDerivs 3.0 Unported" license.

For peer review only
